# Nucleotide-amino acid π-stacking interactions initiate photo cross-linking in RNA-protein complexes

**DOI:** 10.1038/s41467-022-30284-w

**Published:** 2022-05-17

**Authors:** Anna Knörlein, Chris P. Sarnowski, Tebbe de Vries, Moritz Stoltz, Michael Götze, Ruedi Aebersold, Frédéric H.-T. Allain, Alexander Leitner, Jonathan Hall

**Affiliations:** 1 Department of Chemistry and Applied Biosciences, Institute of Pharmaceutical Sciences, ETH Zurich, Zurich, Switzerland; 2 Department of Biology, Institute of Molecular Systems Biology, ETH Zurich, Zurich, Switzerland; 3 Department of Biology, Institute of Biochemistry, ETH Zurich, Zurich, Switzerland; 4 Faculty of Science, University of Zurich, Zurich, Switzerland; 5 Present Address: Eawag, Swiss Federal Institute of Aquatic Science and Technology, Dübendorf, Switzerland; 6 Present Address: Department of Biology, Chemistry and Pharmacy, Institute of Chemistry and Biochemistry, Free University Berlin, Berlin, Germany

**Keywords:** RNA-binding proteins, RNA

## Abstract

Photo-induced cross-linking is a mainstay technique to characterize RNA-protein interactions. However, UV-induced cross-linking between RNA and proteins at “zero-distance” is poorly understood. Here, we investigate cross-linking of the RBFOX alternative splicing factor with its hepta-ribonucleotide binding element as a model system. We examine the influence of nucleobase, nucleotide position and amino acid composition using CLIR-MS technology (crosslinking-of-isotope-labelled-RNA-and-tandem-mass-spectrometry), that locates cross-links on RNA and protein with site-specific resolution. Surprisingly, cross-linking occurs only at nucleotides that are π-stacked to phenylalanines. Notably, this π-stacking interaction is also necessary for the amino-acids flanking phenylalanines to partake in UV-cross-linking. We confirmed these observations in several published datasets where cross-linking sites could be mapped to a high resolution structure. We hypothesize that π-stacking to aromatic amino acids activates cross-linking in RNA-protein complexes, whereafter nucleotide and peptide radicals recombine. These findings will facilitate interpretation of cross-linking data from structural studies and from genome-wide datasets generated using CLIP (cross-linking-and-immunoprecipitation) methods.

## Abstract (web summary)

Although UV-induced cross-linking is a widely used method to study RNA-protein complexes, the cross-linking reactions are poorly understood. Here, the authors show that π-stacking interactions between nucleobases and aromatic amino acids play a key role in the cross-linking process.

## Introduction

The human genome encodes more than 1500 RNA binding proteins (RBPs) that regulate key processes, including translation, localisation, stability and splicing^1–3^. In order to understand fully the structure-function relationship of an RBP, it is necessary to identify to which RNAs it binds in vivo, and how non-covalent interactions occur in the binding site. RNA-protein binding occurs at conserved RNA binding domains, such as RNA recognition motifs (RRM), heterogeneous nuclear ribonucleoprotein (hnRNP) K-homology domains and zinc finger (ZnF) domains^4,5^. These domains recognize short, usually single-stranded regions of 3–8 nucleotides (nt) known collectively as consensus RNA binding elements (RBE)^6,7^ that often contain degenerate positions. Additional binding affinity and selectivity can be generated via supplementary contacts between the RNA and the protein^5,8^; for example, the RBP FUS has a bipartite binding mode comprising its ZnF domain and its RRM^9^. RNA-protein binding has also been observed with proteins that lack canonical RNA binding domains (RBDs)^1^. Taken together, these features render difficult the prediction of an RBP’s substrates based only on a computational search for its consensus RBE. Indeed, recent studies of the RBFOX protein family showed that only one-half of the isolated RNA targets contain the RBFOX consensus binding motif and that other motifs presumably are responsible for some of its splicing activities^10,11^.

Many state-of-the-art methods to identify RNA-protein interactions in vivo employ RNA-protein cross-linking induced by UV light^12–15^. For example, by combining UV cross-linking with mass spectrometry approaches, proteins bound to given RNAs can be identified^16–21^. Conversely, UV cross-linking and immunoprecipitation (CLIP) protocols are commonly used to identify RNA-binding sites for given proteins on a transcriptome-wide scale^22–26^. Technical advances constantly improve these techniques^17,27,28^, however, a long-standing challenge in structure/mechanism-oriented studies is to identify the points of cross-linking on both the RNA and the protein with site-specific resolution. Recently, we (RA, AL, FA) introduced cross-linking of segmentally isotope-labelled RNA and tandem mass spectrometry (CLIR-MS), which identifies the sites of amino acid/ribonucleotide cross-links in a single protocol^28^.

The photo-induced reaction between amino acids and ribonucleotides occurs between free radical species at “zero distance”^29–31^. Reactions involve mainly uridines and guanosines^18,32,33^, but most amino acids can participate^17,18^. Nevertheless, cross-links typically only occur at specific positions in the RNA-RBP motif, for which there is currently no mechanistic rationale^34^. Moreover, it has proven difficult to investigate and identify factors that promote cross-linking, largely because i) the RNA-protein binding site environment, which is critical for cross-linking chemistry, cannot be simulated in simple solvents, and ii) the chemistry usually produces complex product mixtures that are difficult to characterize on a background of protein and nucleic acid UV damage^35^.

Here, we investigate the structural requirements for the cross-linking of an RNA to its RBP partner. We use the RRM domain of the RBFOX family (FOX_RRM_) and its RNA consensus binding motif U_1_G_2_C_3_A_4_U_5_G_6_U_7_ (FOX_RBE_) as a model system, exploiting the high affinity of the complex forms and its well-characterized NMR structure^36^. We introduce ^13^C-labelled ribonucleotides into the FOX_RBE_ heptanucleotide and use CLIR-MS to identify RNA-protein cross-links with site-specific resolution. Cross-linking on the protein clusters at amino acids around two phenylalanines, consistent with previous findings^37^. However, with few exceptions, it only occurs on the RNA at U_1_, G_2_ and G_6_. We then employ site-specific mutagenesis to probe systematically the influence of nucleobase, nucleotide position and amino acid composition on the cross-linking profile. This reveals that cross-linking only occurs with guanosine or uridine at three of the seven nucleotide positions, and only when bases are stacked to aromatic amino acid side chains. Remarkably, this primary stacking interaction is required for neighbouring amino acids to participate in cross-linking. We identify and confirm the importance of this structural feature in selected published examples from other groups as well with an unbiased analysis of three large datasets, suggesting that it is of primary importance for zero-length cross-linking in native RNA-protein binding sites. Moreover, we expect that this finding will facilitate the interpretation of RNA-protein cross-linking data, especially for non-canonical binding motifs. It will also help guide the design of future cross-linking experiments and will aid the development of new tools for de novo motif discovery (see ref. ^33^).

## Results

### Optimization of CLIR-MS to identify RNA-protein cross-links with site-specific resolution

**Fig. 1:**
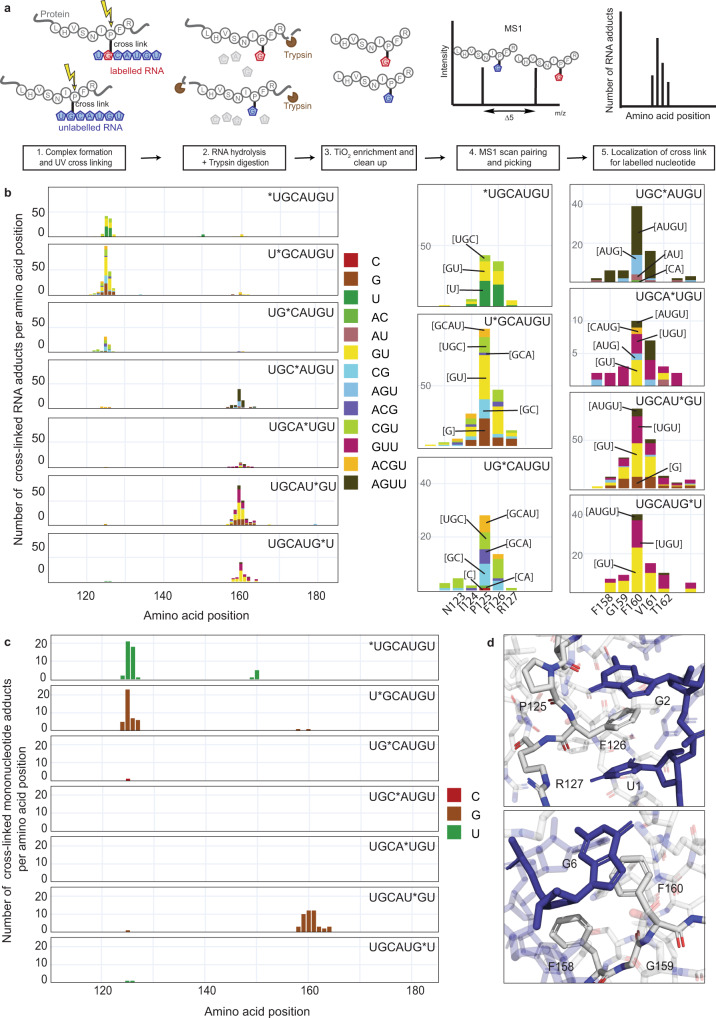
Photo-induced cross-linking of FOX_RBE_ and FOX_RRM_ occurs only at U_1_, G_2_ and G_6_. **a** Individual steps of the CLIR-MS protocol. **b** CLIR-MS analysis of site-specifically isotope-labelled FOX_RBE_ and FOX_RRM._ Plots show the number of RNA adducts at each amino acid position. Phenylalanines are at positions 126, 158 and 160. The xQuest software was used to search for cross-linked mono-, di-, tri- and tetra-nucleotide adducts that are present in FOX_RBE_ which are colour coded (see also Supporting Data 1) (*N indicates a ^13^C-labelled nucleotide). The main cross-linking clusters of each FOX_RBE_ variant are shown enlarged on the right side. **c** Data of b) filtered for mononucleotides showing the cross-links to U_1_, G_2_ and G_6_ (see Supporting Data 1). **d** Structure of FOX_RRM_/FOX_RBE_^36^ (PDB ID: 2ERR) showing F126 and U_1_/G_2_ (upper panel), and F160 and G_6_ (lower panel) around which the cross-linking is clustered. Structures were visualized with PyMOL (PyMOL Molecular Graphics System, Version 2.5 Schrödinger, LLC).

The original CLIR-MS protocol (Fig. 1a) employs RNAs with contiguous regions of differentially isotope-labelled nucleotides in the cross-linking step^28^. After partial RNA and protein digestion, short peptide-oligonucleotide conjugates are identified as matched signal pairs in the precursor ion mass spectrum, which localizes the cross-linked nucleotide to the labelled RNA segment. Overlapping partial sequences then facilitates the localization of the cross-link on the RNA. A drawback of the original implementation of CLIR-MS is the inherent requirement for enzymatic ^13^C/^15^N-labelled RNA synthesis (i.e. in vitro transcription). This does not allow site-specific nucleotide labelling which is needed to unambiguously assign the reactive nucleotide. A second limitation is the nuclease digestion step, which typically produces short oligonucleotides (i.e., 1–4 nt) and is probably less efficient on nucleotides that are structurally changed by cross-linking^17^. In this study, we implemented chemical solutions to help circumvent these problems; we employed ^13^C-labelled phosphoramidites (Fig. S1a) during solid-phase RNA synthesis to incorporate labelled nucleotides site-specifically^38^; and we switched from RNase digestion to alkaline hydrolysis of RNA, while exercising care not to degrade the protein or the nucleobases. Consequently, the mass analysis of the product mixtures yielded a greater fraction of peptide-mononucleotide adducts, allowing us to better identify nucleotides that are cross-linked (Fig. S1b).

### U_1_, G_2_ and G_6_ in FOX_RBE_ cross-link to amino acids centred on phenylalanines in the FOX_RRM_

We employed a systematic approach in an effort to identify key structural requirements for cross-linking of RBFOX to RNA. We first used ^13^C-labelled versions of FOX_RBE_ to identify all points of reaction between the RNA and the protein. We then synthesized mutated variants of FOX_RBE_ to determine how cross-linking varies with respect to i) the nucleobase, ii) its positions in the RBE, and iii) how it is affected by amino acid composition in the binding site. We were mindful of the fact that mutating sites in the RNA and the protein might alter the mode of (or even abolish) RNA-protein binding, and therefore for each mutant we measured the binding affinity to FOX_RRM_ using surface plasmon resonance spectroscopy (SPR).

We synthesized the seven ^13^C-labelled versions of FOX_RBE_ and confirmed the correct incorporation of the label by liquid-chromatography mass spectrometry (LC-MS) (Fig. S2**)**. We incubated each version of FOX_RBE_ with FOX_RRM_ and performed the CLIR-MS protocol. The mass analysis identified short oligonucleotide fragments cross-linked to peptides in clusters close to F126 and F160 (Fig. 1b). Each oligonucleotide signal in the spectrum of Fig. 1b was detected because it contains a ^13^C labelled ribose. However, other than for mononucleotides, the actual site of cross-linking in the fragment could not be called; for example, the tetranucleotide fragment containing A, C, G and U might have cross-linked at any of the four bases (A, G, C or U). We noted that all (>99%) of the detected fragments contained at least one uridine or guanosine, consistent with literature reports^15,18,32,33,39^ that uracil and guanine mainly participate in cross-linking. Here, the use of alkaline hydrolysis for RNA digestion proved advantageous, since it digests a larger fraction of the RNA to mononucleotides (see Fig. S1b, c), thereby identifying unambiguously the cross-linking sites. Hence, focusing only on the mononucleotide species in the spectra of Fig. 1b, revealed that cross-linking in the FOX_RBE_ involved almost uniquely U_1_, G_2_ and G_6_ (Fig. 1c). The absolute numbers of cross-links were in a similar range for the three nucleotides, although numbers of cross-links cannot be confidently compared between different experiments using the current CLIR-MS protocols.

The cross-linking of G_2_ and G_6_ was consistent with published CLIP data^33,40^ (Fig. 1c). G_2_ and G_6_ are critical to the binding of FOX_RBE_ to FOX_RRM_, and their exchange for A_2_ or A_6_ respectively, greatly reduced protein-binding and cross-linking between the amino acid clusters 126 and 160 and the mutated sites (Fig. S3). Although cross-linking from U_1_ was detected in the CLIR-MS experiments, it was hardly observed at U_5_ (*vide infra*) or U_7_, consistent with our hypothesis that strict structural parameters govern the photo-induced reactions between FOX_RRM_ and FOX_RBE_. Isolated cross-links were also observed in some of the spectra of Fig. 1b, c. Although low numbers of cross-links must be considered with caution, their locations suggested that in several cases they were not artifacts. In particular, the cross-links at F160 seen with *UGCAUGU and U*GCAUGU (Fig. 1b, top two panels) are consistent with transient (low affinity) binding of U_1_G_2_ in the binding pocket occupied mainly by U_5_G_6_. Likewise, cross-links around F126 in the lower panels of Fig. 1b,c may derive from similarly transient contacts with U_5_G_6_U_7_. One cross-link from P125 to cytidine is visible in Fig. 1c. A_4_ did not cross-link to FOX_RRM_ (Fig. 1c). Sites of cross-linking at the protein were centered at two phenylalanines (F126 and F160), with a distribution of 1-3 amino acids flanking these positions^37^. This was confirmed from analysis of the MS/MS spectra in which fragment ions localize the RNA adducts unambiguously on the peptide backbone (Fig. S4).

The cross-links of U_1_, G_2_ and G_6_ aligned well with the NMR structure of FOX_RRM_ bound to FOX_RBE_^36^ (Fig. 1d) (PDB ID: 2ERR).The largest number of spectra corresponded to U_1_ reacting with P125 and F126, and to a lesser extent with I124 and R127 (Fig. 1c). Similarly, G_2_ cross-linked to I124, P125, F126 and R127. Of note, U_1_ and G_2_ each stack to one face of F126. Hydrogen bonds are also present between the bases of U_1_ and G_2_, and between R127 and I124, respectively. G_6_ reacted with F160 (to which it also stacks), as well as with neighbouring amino acids at positions 158-164; F158 contacts the ribose of G_6_. Notably, several close RNA-protein contacts that are visible in the NMR structure (i.e. C_3_ interacting with F126 (but not stacking), G_6_ stacking with R194 and U_5_ stacking to H120)^36^, did not produce extensive cross-linking.

The current understanding of RNA-protein cross-linking is that close contact between nucleotides and amino acids is the main pre-requisite for a cross-linking event^41,42^. However, only three from the seven nucleotides of FOX_RBE_ engaged in efficient cross-linking, despite close contacts between all nucleotides and amino acids in the binding site. Hence, we investigated two obvious parameters that could influence cross-linking: i) the chemical reactivities of the nucleobases and the amino acids, and ii) the relative positioning of the reactive pair. By mutating selected nucleotides and amino acids in the binding pocket, we created a cross-linking structure-activity relationship for the FOX_RRM_-FOX_RBE_ interaction.

### Only uridine cross-links to FOX_RRM_ from position 1 of FOX_RBE_

**Fig. 2:**
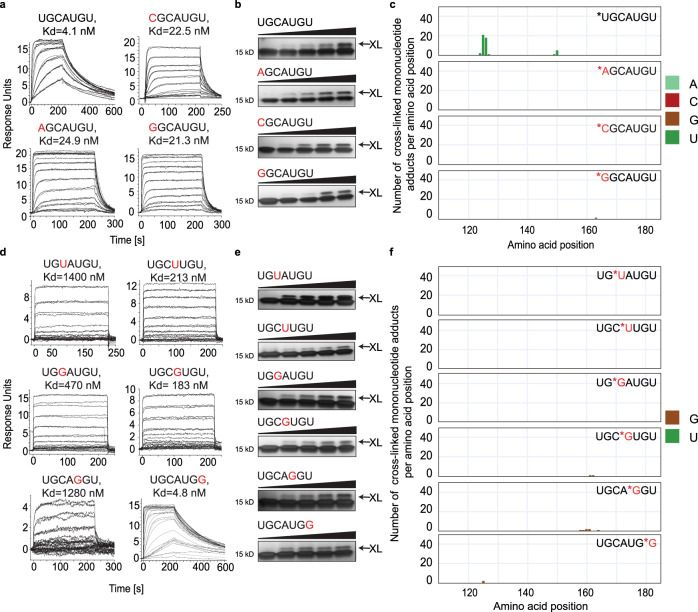
FOX_RRM_/FOX_RBE_ cross-linking is nucleotide- and site-specific. **a** SPR sensorgrams of FOX_RBE_ variants bound to FOX_RRM_. **b** SDS-PAGE gels showing that all N_1_-variants undergo cross-linking with FOX_RRM_ with increasing radiation. The cross-linking product band is indicated by “XL” (repeated three times). **c** CLIR-MS plots show that cross-linking occurs at U_1_ of FOX_RBE_, but not with N_1_-mutants (the xQuest software was used to search for cross-linked mono-, di-, tri- and tetra-nucleotide adducts and the data was filtered for mononucleotides that are present in FOX_RBE_ mutants which are colour coded (*N indicates a ^13^C-labelled nucleotide, mutated nucleotides are labelled in red) (see Supporting Data 1). **d** SPR sensorgrams show that U or G mutations at N_3_, N_4_, N_5_ and N_7_ of FOX_RBE_ attenuate, but do not abolish, FOX_RRM_ binding to FOX_RBE_ variants. **e** SDS-PAGE gels show that FOX_RBE_ variants cross-link to FOX_RRM_ with increasing irradiation (repeated three times). **f** CLIR-MS plots of singly-labelled FOX_RBE_ variants show that protein-RNA cross-linking does not occur with U or G nucleotides located at at N_3_, N_4_, N_5_ and N_7_ (see Supporting Data 1). (The xQuest software searches for cross-linked all mono-, di-, tri- and tetra-nucleotide that are present in FOX_RBE_ mutants, and the data was filtered for mononucleotides which are colour coded).

We synthesized the three labelled mutants of *NGCAUGU (N = A, G, C; Supporting Table 1), as well as the corresponding per-labelled control sequences *N*G*C*A*U*G*U. We first confirmed that the NGCAUGU variants bound to FOX_RRM_ using SPR. In this assay, parent UGCAUGU bound strongly to FOX_RRM_ with a *K*_d_ = 4.1 nM. Substitution of the 5’-uridine reduced the strength of the interaction by 4-6-fold for the three variants (AGCAUGU: *K*_d_ = 24.9 nM; CGCAUGU: *K*_d_ = 22.5 nM; GGCAUGU: *K*_d_ = 21.3 nM) (Fig. 2a). This was consistent with the NMR structure showing that the 5’-uridine of FOX_RBE_ contributes to binding by π-stacking to F126 (Fig. 1d).

Next, we incubated the RNAs together with FOX_RRM_ and irradiated the complexes with increasing doses. Work-up and analysis by SDS-PAGE for the three NGCAUGU mutants revealed a new slow-migrating band on the gels, similar to that of the wild-type FOX_RBE_ (N = U), consistent with RNA-protein cross-linking (Fig. 2b). The appearance of a band on an SDS-PAGE confirms that cross-linking occurs, but it does not identify the site of cross-linking nor the composition of the product. In order to determine whether the mutants cross-linked at the N_1_-position, we turned to CLIR-MS (Figs. S5, and S6). CLIR-MS data for per-labelled *N*G*C*A*U*G*U confirmed that the three FOX_RBE_ mutants exhibit the same cross-linking “fingerprint” as wild type FOX_RBE_, i.e. in the same two amino acid clusters around positions 126 and 160 (Fig. S6a). However, in order to differentiate cross-linking of N_1_ to that from G_2_ in the 126-cluster, we performed CLIR-MS on the singly labelled sequences (*NGCAUGU). In contrast to U_1_, cross-linking hardly occurred at A_1_, G_1_ or C_1_ (Figs. 2c, S5), confirming the high reactivity of uridine in photo-reactions^18,32^. Nevertheless, it was surprising that G_1_ was unreactive given the reactivity of G_2_, which may have been due to inappropriate orbital overlap in stacking.

In order to determine systematically the propensity for cross-linking at each position in FOX_RBE_ when a photoreactive nucleotide (i.e. U or G) is present, we performed CLIR-MS on six additional positional FOX_RBE_ mutants. Thus, we exchanged *U for C_3_ and A_4_ in FOX_RBE_ (UG*UAUGU, UGC*UUGU, resp.), and *G for C_3_, A_4_, U_5_ and U_7_ (UG*GAUGU, UGC*GUGU, UGCA*GGU, UGCAUG*G, resp.). In each case, we first confirmed that the mutants bound and cross-linked to FOX_RRM_ using SPR and SDS-PAGE gels (Fig. 2d, e, resp., Fig. S6a). Remarkably, in none of these six examples, did the mutated nucleotides cross-link efficiently to the protein (Fig. 2f). The lack of reactivity at U_3_ (in UG*UAUGU) was particularly surprising given the close proximity of C_3_ to F126 in the NMR structure.

In summary, while G_2_ and G_6_ in wild type FOX_RBE_ cross-linked to FOX_RRM_, guanosine did not cross-link efficiently at any other of the other five locations in the FOX_RBE_. Similarly, uridine readily cross-linked to FOX_RRM_ from position N_1_ - where A, C and G were unreactive - but not from the four other locations in the FOX_RBE_. Taken together, the data from this controlled model study confirmed that RNA-protein cross-linking events have strict requirements, beyond simply the proximity of a reactive nucleotide and a reactive amino acid.

### Aromatic amino acids play a key role in priming RNA-protein cross-linking reactions

Analysis of the aforementioned CLIR-MS data (Figs. 1c, and S6) provided two important insights: i) on the RNA side, strong cross-linking only occurred with nucleotides that were stacked to aromatic amino acids; and ii) on the protein side, cross-links involved F126 and F160, but also upto three amino-acids up- and downstream of F126 and F160.

**Fig. 3:**
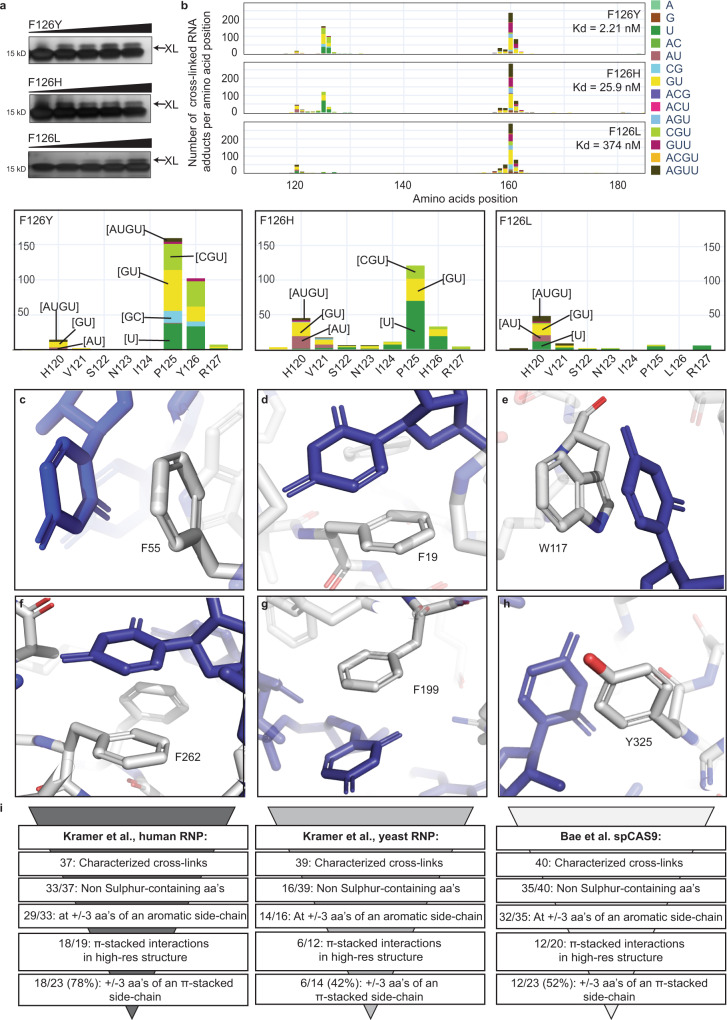
Proximal aromatic amino acids mediate cross-linking in RNA protein complexes. **a** F126 mutants undergo binding and cross-linking with FOX_RBE_ with increasing irradiation, as shown by SDS-PAGE. The upper cross-linked RNA-protein band is indicated by “XL” (repeated three times). **b** CLIR-MS plots show the number of RNA adducts at each amino acid position of FOX_RRM_ mutants (the xQuest software was used to search for cross-linked mono-, di-, tri- and tetra-nucleotide adducts that are present in FOX_RBE_ which are colour coded; see also Supporting Data 1). Binding affinities of FOX_RBE_ are taken from ref. ^36^. **c** Structure of LIN28 in complex with preEM-let-7f obtained by Nam et al.^50,51^. (PDB ID: 3TS0). **d** Structure of hnRNP C binding on AUUUUUC obtained by Cienikova et al.^53,54^. (PDB ID: 2MXY). **e** Structure of the 40 S ribosomal protein S1 in complex with the 18 S rRNA obtained by Ben-Shem et al^18,55^. (PDB ID: 4V88). **f**, **g** Structure of the U2AF binding to poly-U RNA obtained by Mackereth et al.^18,56^. (PDB ID: 2YH1). **h** Structure of the Streptococcus pyogenes Cas9 (spCas9) protein in complex with sgRNA obtained by Jiang et al^57^. (PDB ID: 4ZT0) (all structures are visualized with PyMOL (PyMOL Molecular Graphics System, Version 2.5 Schrödinger, LLC)). **i** The proteome-wide cross-linking data of human and yeast RNP from Kramer et al.^18^ and the cross-linking data of bacterical protein spCAS9 from Bae et al.^17^ were manually curated for π-stacking interactions in proximity to characterized, cross-linked (non-S-containing) amino acids. Cross-links were filtered for those falling within of ±3 residues of an aromatic residue and overlaid with available high-resolution structures to observe possible π-stacking.

We therefore mutated F126 in FOX_RRM_ to histidine, tyrosine and leucine. An effort to perform CLIR-MS on a tryptophan mutant failed because of protein precipitation. We have previously shown using SPR that aromatic amino acids at position 126 are crucial for binding FOX_RBE_ (F126Y: *K*_d_ = 2.21 nM; F126H: *K*_d_ = 25.9 nM), although a sterically-fitting aliphatic amino acid such as leucine can partially substitute for the phenylalanine (F126L: *K*_d_ = 374 nM)^36^. We irradiated these variants in the presence of FOX_RBE_. All three protein mutants cross-linked to FOX_RBE_, as evident from SDS-PAGE (Fig. 3a). Next, we carried out CLIR-MS experiments with uniformly ^13^C-labelled FOX_RBE_. F126Y and F126H cross-linked to the FOX_RBE_ similarly to FOX_RRM_ (Fig. 3b). The cross-linking profile was similar for the three complexes at F126 and F160. However, when phenylalanine was exchanged for leucine, binding was weaker and the cross-linking to position 126 was abolished. Notably, cross-linking to the neighbouring amino acids 124-127 was also mostly lost for F126L (Fig. 3b), confirming the primary role of the aromatic side chain in mediating the cross-linking reactions with flanking amino acids at positions 124, 125 and 127. Interestingly, the F126H mutant appears not to cross-link to G_2_, as shown by the absence of G mononucleotides (brown) or CG dinucleotides (turquoise) in Fig. 3b (Supporting Data 1). Although we do not have supporting data, nor know of any precedence in literature, it is plausible that the histidine has a different cross-linking preference to those of tyrosine or phenylalanine and/or that stacking to the guanosine G_2_ is disturbed in this particular complex. Unexpectedly, a H120 cross-link occurred with the three FOX_RRM_ mutants, which was hardly observable in the wild type FOX_RRM_ (Fig. 3b). Analysis of the oligonucleotide fragments in Fig. 3b strongly suggested that the cross-link occurred with U_5_. In fact, the NMR structure of FOX_RRM_-FOX_RBE_ shows that U_5_ adopts a stacking arrangement with H120, and thus might have been expected to cross-link in the wild type FOX_RBE_-FOX_RRM_ interaction (Fig. S7). Together, the data obtained from these RNA- and protein mutants suggests that π-stacking interactions between aromatic amino acids (e.g. phenylalanine, tyrosine or histidine) and guanosines or uridines are an important pre-requisite for their cross-linking, not only to the aromatic side chains, but also to the flanking amino acids. Clearly, our findings do not speak to all cross-linking reactions in RNA-protein complexes, for instance those involving sulfur-containing amino-acids, such as cysteine, which is not present in the FOX_RRM_, but which is prone to cross-link probably due to the high reactivity of the thiyl radical^18,30,43^.

### RNA-protein cross-linking correlates with π-stacking interactions in other complexes

In order to determine whether these findings apply more broadly to RNA-protein cross-linking, we examined CLIR-MS data from the alternative splicing factor PTBP1 in complex with the internal ribosomal entry site (IRES) of encephalomyocarditis virus (EMCV)^28^. Cross-links mainly clustered around five aromatic amino acids (Y127, Y267, F371, H411, H457), and comprised uridines, as reported by ref. ^28^. Correlating these observations with the NMR solution structure of PTBP1 bound to short poly-pyrimidine sequences (CUCUCU)^44^, confirmed that these amino acids were indeed all π-stacked to pyrimidines (PDB IDs: 2AD9, 2ADB, 2ADC) (Fig. S8), with cross-linking extended for a few amino acids along the protein backbone, flanking the aromatic side chains. Tyrosines Y127, Y267 and histidine H411 π-stack to uridines in CUCUCU, consistent with uridine-containing cross-links in the CLIR-MS dataset from the IRES of EMCV (Fig. S8b). Intriguingly, however, histidine H457 π-stacks to cytidines in the IRES^28^ and CUCUCU^44^, but produces high numbers of U- and UU-containing cross-links in the CLIR-MS dataset^28^. Likewise, Cléry *et al* observed by NMR spectroscopy a π-stacking of cytidine to Y19 in the RRM of SRSF1^45^, whereas Kramer et al. found a uridine cross-linked to the same amino acid^18^. These observations suggested that C-to-U conversion (i.e. hydrolysis) might occur at π-stacked cytidines during cross-linking or in sample work-up/analysis. Although the cytosine group itself is stable to the conditions used to fragment RNA by base hydrolysis or enzyme digestion (refs. ^46,47^), the exocylic amino group of cytidine is more susceptible to hydrolysis when its 5–6 double bond is reduced, i.e. in dihydrocytidine^48,49^ (Fig. S6b). Since cross-linking reactions may produce intermediates or final products in which the cytidine 5–6 carbon-carbon bond is saturated, it is plausible that C-to-U conversion only occurs at π-stacked/cross-linked cytidines. Hence, mindful of the minor differences in the masses of cytidine/uridine-containing fragments, we searched our datasets for supporting evidence of this, using an appropriate set of parameters for the xQuest software. We did not observe significant ^13^C-to-^13^U hydrolysis using CLIR-MS on UG*CAUGU and the FOX_RBE_ mutant *CGCAUGU. However, this might have been because neither of these cytidines underwent efficient cross-linking/π-stacking to FOX_RRM_ (UG*CAUGU: Fig. 1c, third panel; *CGCAUGU: Fig. S5, second panel). Therefore, we also analyzed additional CLIR-MS data from four fully ^13^C-labelled Fox_RBE_ mutants bearing cytidines at positions N_4_, N_5_, N_6_ and N_7_ (Fig. S6c). Indeed, we found that two of the mutants (UGCACGU and UGCAUCU) produced large numbers of cross-links that - consistent with the NMR structure - could only have derived after C-to-U conversion; for example, AU and AUGU from UGCACGU, bound to H120; and AUUU and UUU from UGCAUCU, bound to F160 (Fig. S6c–f). Taking together the data from the PTBP1 study, that of SRSF1^18,45^ and that of these six FOX_RBE_ RNAs, we concluded that cytidine likely undergoes partial hydrolysis mainly at positions in an RNA where it π-stacks and cross-links to the protein; for FOX_RBE_, at positions N_5_ and N_6_, but not at positions N_3_, N_4_ and N_7_. In contrast to previous assumptions^15,17^, these findings provide direct mass-spectrometry evidence that cytidine in RNA-protein complexes readily participates in photo-induced cross-linking, especially when it is π-stacked. However, this renders it susceptible to hydrolysis to uridine, which confounds its detection and in some cases may even lead to mis-assignments during RNA-protein modeling.

Next, we sought to confirm the importance of π-stacking to RNA-protein cross-linking in datasets that were generated using techniques other than CLIR-MS. Thus, we searched for structurally well-characterized examples in literature that would speak to the generalization of our findings. A unique strength of the CLIR-MS technique is that in many cases it is possible to simultaneously identify both the precise amino acid and the ribonucleotide in a cross-linked fragment. Indeed, we identified only one published example where cross-linking at both the ribonucleotide and the amino acid were unambiguously defined by isoptopic labelling, and where these sites could be mapped to a high resolution structure. In this case, ^18^O-RNA labelling and targeted mass spectrometry were used to localise the cross-link of U_11_ in a let-7 microRNA to a π-stacked phenylalanine (F55) in the LIN28 cold shock domain (Fig. 3c)^50–52^. On the other hand, we found numerous examples where amino acids involved in stacking interactions (predominantly with uracils) underwent UV-cross-linking, most likely with the same uracil but not unambiguously proven by nucleotide labelling. For example, Panhale et al. report a cross-link between F19 and a uridine in hnRNPC, from which the NMR structure with poly-U sequences confirms the π-stacking interaction with F19^53,54^ (Fig. 3d). Kramer *et al*. used a sophisticated workflow to pin-point cross-linking sites on a broad scale from ribonuclear protein complexes (RNPs) isolated from human and yeast cells^18^. By correlating their cross-linking data from ribosomal yeast protein S1 with the crystal structure of the protein (PDB ID: [4V88]) (Fig. 3e)^55^, we confirmed that tryptophan W117 π-stacks and cross-links to uridine U_1799_ from ribosomal S1. Similarly, the same group localised RNA cross-links on the human splicing factor U2AF 65-kDa subunit to amino acids L261, F262 and F199; according to the crystal structure, F262 and F199 both π-stack to uridines in complex with poly-U RNA (Fig. 3f, g) (PDB ID: 2YH1)^56^. Bae *et al*. showed that tyrosines Y325 (Fig. 3h), Y450 and Y1356 in the *Streptococcus pyogenes* Cas9 (spCas9) protein all cross-link with RNA^17^; the crystal structure of spCas9 shows that all three residues are π-stacked to uridines or guanosines^57^.

These well-characterized, selected examples already provided supporting evidence for the generality of our findings. However, the aforementioned examples of Kramer et al. and Bae et al. were extracted from large well annotated datasets in which, collectively, more than 100 RNA-protein cross-links from a wide variety of RBPs are catalogued. These datasets therefore offered an opportunity to analyze in an unbiased fashion the putative link between π-stacking and cross-linking. The two proteome-wide datasets reported by Kramer et al. each comprise approximately 60 RNA-protein cross-links generated from affinity-captured nuclear pre-mRNAs from human cells^18^ and from pre/mRNAs of yeast cells^18^. The third dataset reports 84 cross-links to spCAS9, which forms a complex with single guide RNAs^17^. We manually annotated each of the three datasets in a systematic fashion in order to determine whether amino acids that undergo cross-linking are located within + /−3 positions of an aromatic amino acid side chain (mindful that in a fully random sequence, 20% of the amino acids may be aromatic) and if yes, whether said aromatic side-chains π-stack to nucleobases.

The human RNP dataset^18^ details 60 cross-links to approximately 35 proteins, with 37 cross-links that are localized on defined amino acids, in mostly RRM binding domains (Supporting Data 4). From these, 33 cross-links are assigned to non-cysteine and non-methionine amino acids (Fig. 3i; Supporting Data 4), 29 of which are located within three amino acids of an aromatic side chain. High-resolution structures were informative for 19 of these amino acids and, pleasingly, showed that 18 of the aromatic side chains were involved in apparent π-stacking interactions, and one which was not. Taking into account also the four cross-links which are not close to an aromatic amino acid, means that 18/23 (78%) cross-links occur close to a π-stacked aromatic side chain, fully consistent with our findings. In this dataset, neither of the KH domain-bearing proteins carry aromatic amino acids close to cross-links, although both underwent cross-linking to cysteines, demonstrating that cysteine does not follow the pattern, as expected. In contrast, a positive π-stacking/cross-linking association (to an adenosine) was present for the cold shock domain of Y-box binding protein, as well as for ribosomal proteins S2, L5, L6 and L34 with distinct domains.

The yeast RNP dataset^18^ contains 39 defined cross-links to 52 proteins, containing a variety of domains (Supporting Data 4). Surprisingly, 23/39 cross-links involve cysteines, which the authors suggested might be due to the present of dithiothreitol (DDT) in the yeast sample which is known to promote cross-links involving cysteine residues^18,58^. Fourteen cross-links lie within three amino acids of an aromatic side-chain, for which 12 high-resolution structures are available. These show that six cross-links occur at apparent π-stacking interactions, and for two cross-links high-resolution structures are not available (Fig. 3i; Supporting Data 4). Finally, the outcome of cross-linking of the spCAS9 protein to RNA was reported by ref. ^17^. Cross-links comprising 40 amino acids were catalogued, of which five were cysteine or methionines and were discarded from further analysis. Of these 35 cross-links, 32 lie within three amino acids of an aromatic side chain and of these, 20 can be studied with the high-resolution structure. Twelve of the cross-links involve apparent π-stacking interactions (Fig. 3i; Supporting Data 4), whereas eleven cross-links are not close to a π-stacking interaction. In summary, this unbiased analysis confirmed the association of cross-linking with π-stacking in a variety of RNA-binding domains for totals of 78, 42 and 52% of the cross-links in studies performed by independent groups in yeast, bacterial and human systems.

Taken together with the aforementioned specific examples from literature and our analysis of the FOX_RRM_ and PTPB1 CLIR-MS data, the data overall strongly supports the importance of π-stacking to the RNA-protein cross-linking chemistry. The absence of a positive correlation for some cross-links may be due to a variety of reasons; e.g. different conditions for protein structures/domain determination in vitro and cross-linking experiments performed in vivo on protein complexes; or cross-linking reactions that occur as a result of transient interactions (i.e. artifacts). In addition, the lack of structural information for several cross-links in the human RNP and spCAS9 datasets may have prevented an even higher correlation. Finally, it is also apparent that a π-stacking interaction is not a strict requirement for all cross-linking events. Cysteine, which is prone to cross-linking, does not require a π-stacking interaction in order to produce a long-lived, highly reactive radical^18,30,43^. This is consistent with the lack of aromatic amino acids proximal to cysteine-containing cross-links in the KH domains of proteins in the yeast and human RNP data-sets^18^. Thiol-containing molecules present in buffer may also initiate UV-induced cross-linking of proteins and nucleic acids^58^. In addition, recent publications described cross-linking of dsDNA to histones using conventional cross-linking^59^, where π-stacking of the side chain is more difficult to envision because of the double-stranded helical structure; although this may partly explain why double-stranded oligonucleotides are reported to cross-link less efficiently than single-stranded oligonucleotides^60,61^.

### Photo-induced electron transfer in a π-stacked RNA-protein complex may mediate radical reactions of cross-linking

**Fig. 4:**
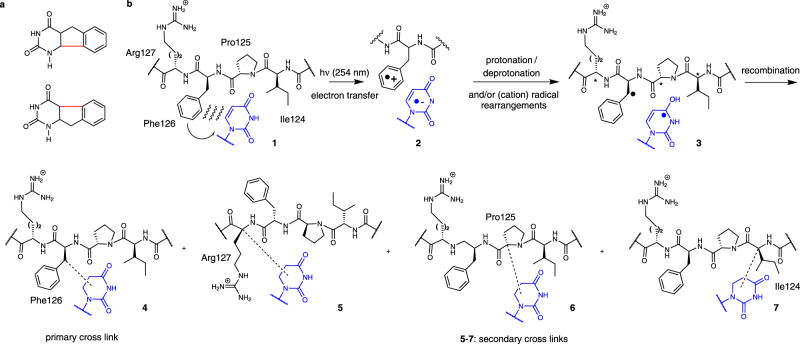
Proposed mechanism for UV induced RNA-protein cross-linking. **a** UV induced cyclization of 5-benzyluracil and 6-benzyluracil after Sun et.al.^35^. (bonds in red are formed upon cyclisation). **b** Possible mechanism of UV cross-linking between the stacked F126 und U_1_ of the FOX_RRM_ (1). Photo-induced electron transfer leads to a radical ion pair (2). After protonation/deprotonation steps, the radicals on the benzylic position of F126 and C4 of uridine (3) recombine to yield direct cross-links (4). Indirect cross-links between U_1_ and R127 (5), P125 (6) or I124 (7) may form when the radical cation of F126 oxidizes amide carbonyls from flanking amino acids, which rearrange to radicals stabilized by capto-dative effects at the α-carbons (*).

Free radical reactions of nucleic acids and proteins have been well studied in the context of oxidative damage and electron transfer^43,62^, but less thoroughly investigated for RNA-protein interactions^29,41^. However, a description of the photo-induced intramolecular cyclization of 5-benzyluracil and 6-benzyluracil via benzyl and uracil radical intermediates suggests a plausible model for the cross-linking of U_1_ with F126 (Fig. 4a)^35^. Hence, photo-induced electron transfer between U_1_ and F126 generates a short-lived anion/cation radical pair (exciplex) (Fig. 4b; structures **1** and **2**). Subsequent protonation of the uracil radical anion can yield a neutral α-hydroxy radical^43^, whereas ready deprotonation of the F126 radical cation will produce a stabilized benzylic radical. In the absence of oxygen, the major fate of these free radicals is recombination with the formation of the direct U_1_-F126 cross-link (Fig. 4b; structure **4**). An analogous mechanism has been proposed for the reaction between uracils/halogenated uracils and tyrosine derivatives^31,63^.

Alternatively, the F126 radical, or radical cation, may rearrange to neighboring amino acids in processes mediated by hydrogen atom abstractions^43^, or via oxidation of amide carbonyls (by the F126 radical cation)^64^, yielding free radicals at peptide α-carbon sites on the protein backbone. Viehe *et al* have proposed that α-carbon radicals are especially stabilized thermodynamically by capto-dative effects, i.e. simultaneously by electron-withdrawing (-C = O) and electron-donating (-NR_2_) groups^65^ and, furthermore, that they readily combine with other radicals.

Hence, depending on the lifetimes and the locations of these radicals on the protein backbone, “indirect” cross-links to U_1_ may form, yielding products that are identified by mass spectral analysis after controlled digestion (e.g. structures **5**–**7**; Fig. 4b). These steps are consistent with the outcome of cross-linking reactions of the F126 mutants. Thus, the exchange of phenylalanine for histidine and tyrosine produced similar direct and indirect cross-links, whereas leucine was mostly inactive since its aliphatic side chain cannot partake in the initial electron transfer. Several efforts to mimic some of these cross-linking reactions in solutions were unsuccessful, confirming the crucial role played by the local protein binding site environment. Based on the similarity of the cross-linking profiles from U_1_ and G_2_ (Fig. 1c), it seems intuitively likely that guanosines G_2_ and G_6_ may follow a similar mechanistic reaction path as U_1_. Thus, photo-excitation of the stacked guanine-phenyl ring systems produces free radicals at G_2_ and G_6_, as well as on the peptide backbone around F126 and F160. Recombination yields direct and indirect cross-links, which in the case of G_2_ are to the same α-carbon radicals that couple with U_1_. The nature of the initial exciplex formed from electron-transfer in a stacked guanosine-phenylalanine is unclear, and we were unable to identify a literature precedent for such a mechanism. However, well-cited studies have shown photo-induced electron transfer between π-stacked pyrimidine and purine nucleobases that produce long-lived exciplexes^66,67^. Electron transfer between an amino acid and a nucleotide might be expected to occur in the direction that yields the lowest-energy exciplex. However, due to the special environment of an RNA-protein binding site (see discussions in refs. ^64,66^), this may not necessarily correlate with the measured redox potentials of isolated nucleotides or aromatic amino acid side chains. Together, our observations demonstrate the importance of local environment to cross-linking in the RNA-protein binding site, and at least partly explain why cross-links occur only at specific positions in an RNA-RBP motif.

## Discussion

For a complete understanding of the roles that RBPs play in cellular processes, it is necessary to understand at the atomic level how RNA binding domains in proteins engage with RNAs. RNA-protein interactions are generally characterized in two main ways in vivo: isolating proteins and sequencing the bound RNAs (CLIP methods), and identifying proteins bound to RNAs, for example, by mass spectrometry. Most of these approaches rely upon photo-induced cross-linking, which provides direct evidence of binding under native conditions. However, presently, native cross-linking-based methods suffer from two drawbacks: i) it is challenging to identify simultaneously sites of cross-linking on the RNA and protein, ii) cross-linking in an RNA-RBP motif typically proceeds inefficiently and in an unpredictable fashion. Therefore, any progress that furthers our understanding of this chemistry is of high value.

The CLIR-MS method^28^ employs isotope-labelled RNAs to resolve amino acid/ribonucleotide cross-links in a single protocol, whereby segments of labelled RNA are produced by in vitro transcription prior to ligation-assembly into a full-length RNA. In this study, we have broadened the application of CLIR-MS through the use of chemically synthesized ^13^C-labelled RNAs. This enables site-specific incorporation of labelled nucleotides into the RNA. After irradiation of the RNA-protein complex, and controlled digestion to nucleotide-peptide adducts, the locations of cross-linked nucleotides are pinpointed site-specifically. We demonstrated this methodological advance with a study of the interaction of the RRM domain of the RBFOX family bound to its consensus binding element (U)GCAUGU, for which we have previously determined an NMR structure^36^ and studied cross-linking^37^. Photo-irradiation of the FOX_RRM_-FOX_RBE_ complex led to key observations with potentially wide-ranging implications: 1) strong cross-linking occurred between U_1_, G_2_ and G_6_ with clusters of amino acids centred around the phenylalanines F126 and F160; 2) very little cross-linking was observed at other uridines in the parent or a mutated FOX_RBE_; and 3) amino acids that flank F126 and F160 also cross-linked efficiently to U_1_, G_2_ and G_6_, but not to other nucleotides of FOX_RBE_. Since the NMR structure of FOX_RRM_-FOX_RBE_^36^ shows that U_1_ and G_2_ π-stack to F126, and that G_6_ π-stacks with F160, the data suggested that a π-stacking interaction is a requirement for cross-linking events in an RNA-protein interaction, at least for this RRM domain. Indeed, other aromatic side chains could substitute for F126 in cross-linking, but incorporation of leucine abolished direct and almost all indirect (flanking) cross-linking to U_1_/G_2_. Other researchers have noted in passing the increased presence of aromatic amino acid side chains in UV cross-linking datasets (see refs. ^17,18,29,52,68^), but have not to our knowledge recognized its role as a trigger for cross-linking, nor distinguished between direct and indirect cross-link events. We validated our results on the RBFOX system with the correlation of published cross-linking and structural data from CLIR-MS data generated with the PTBP1 protein, and selected examples from LIN28, hnRNPC, U2AF, ribosomal yeast protein S1 and bacterial spCAS9, that were produced using different methods. Our findings were further strengthened by an unbiased analysis of more than 100 cross-links in large-scale data-sets comprising various RNA-binding domains, where in one case up to 78% of the cross-links showed π-stacking to a proximal aromatic amino acid side chain. It is clear that factors in addition to π-stacking also contribute to cross-linking events in RNA-protein sites, including efficiency of the photo-induced electron transfer between nucleobase and amino acids, the ability to stabilize free radicals, the flexibility of the structure to adopt to the configurations that are required for the radical reactions^69^ and the proximity of reacting radical pairs^70^. Furthermore, our findings do not explain all RNA-protein cross-linking reactions, including those involving cysteine, which is highly photoreactive and prone to cross-link probably due to the high reactivity of the thiyl radical^18,30,43^.

The major findings in this study were enabled by the combination of site-specific labeling with the CLIR-MS protocol, which together provides enhanced knowledge of cross-linking sites at single-nucleotide and amino acid resolution. These included the surprising discovery that cytidine residues which are π-stacked to aromatic residues can undergo partial hydrolysis during photo-induced cross-linking. This observation may explain discordance in some cases between structural- and cross-linking data. Furthermore, the hydrolysis of cytidine should be anticipated in the analysis of CLIR-MS data and may also be relevant to the interpretation of data from CLIP experiments, which is currently an area of intense activity^15^.

CLIR-MS technology is inherently flexible and we are exploring further improvements to the method^37^. However, the method described here requires the use of chemically synthesized, isotope-labelled RNA and is currently restricted to the study of purified individual RNA-protein complexes. Nevertheless, the data produced can aid the interpretation of that from unbiased complex systems. For instance, our findings extended the knowledge on the role of the local environment to cross-linking in the binding site, i.e. beyond the simple proximity of photo-reactive nucleotides and amino acids. This helps at least partly to explain why cross-links occur only at specific sites in an RNA-RBP motif. Furthermore, the localization of π-stacking interactions will aid the interpretation of proteome-wide datasets, for example in cases where proteins lack canonical RNA-binding domains, and in the analysis of CLIP datasets. Thanks to the inherent variations in the ways that RBPs recognize their RNA targets, predictive modeling of RBP selectivity is extremely challenging; our findings can be implemented into the development of new tools^33,71^ for de novo motif discovery.

In a broader sense, the RNA-binding sites of RBPs have garnered attention in the context of disease and drug targeting; for example, the RNA binding site in the intrinisically disordered region of TDP43 contributes to its aggregation in amyotrophic lateral sclerosis (see ref. ^17^). A fuller understanding of how RNA binding domains in proteins engage with RNAs can support the development of new methods of targeting RBPs via the RNA binding site^72^.

## Methods

### Protein expression and purification

The FOX_RRM_ and its mutants were expressed in transformed BL21 Codon+ *Escherichia coli* at 37 °C in LB medium with kanamycin and chloramphenicol^36^. The cells were induced with 1 mM IPTG and after 4 h the cells were harvested by centrifugation. Cells were lysed in 50 mM Na_2_HPO_4_, 1 M NaCl, pH=8 using a cell cracker and the cell lysate was centrifuged at 17 000 rpm at 4 °C for 30 min. The supernatant was purified using a NiNTA affinity column (Ni-NTA agarose, Qiagen). After washing with buffer 50 mM Na_2_HPO_4_, 3 M NaCl, pH=8, the protein was eluted with a step gradient of imidazole (40–500 mM). The purest fractions as judged by 5–20 % SDS–PAGE were combined, and the column was repeated. Pure fractions were dialyzed against 5 L 20 mM NaCl, 10 mM NaH_2_PO_4_, pH=6.5 overnight. The identity of the FOX_RRM_ and its mutants was confirmed using LC-MS/MS measured by top-down analysis and data analysed using ToPIC^73,74^.

For the biotinylated FOX_RRM_, the 15-amino acid *E. coli* biotin ligase recognition sequence GLNDIFEAQKIEWHE was introduced between the TEV cleavage site and the gene encoding FOX-1 using standard PCR mutagenesis. *E. coli* protein ligase BirA was cloned, expressed and purified as previously described^75^. The generation of biotinylated FOX_RRM_ was achieved in a 10 ml batch-mode cell-free synthesis reaction which was conducted for 3.5 h in presence of 2 μM BirA and 400 μM D-biotin^75,76^. The proteins were purified as described above and the biotinylated proteins were cleaved overnight at 4 °C with 0.5 mg TEV protease^76^.

### RNA synthesis

The synthesis of all oligonucleotides was carried out with the MM12 synthesizer (Bio Automation Inc., Plano, TX) on a 50 nmol scale using 500 Å UnyLinker CPG using standard conditions. Synthesis conditions, purification methods and characterisation (Supplementary Table 1, Fig. S10) are listed in supplementary methods.

### Surface Plasmon Resonance Spectroscopy (SPR)

The SPR analysis was carried out on the MASS-1 or SPR-2 from Sierra Sensors (Hamburg, DE). For coating, the amine chip was first treated with PBS buffer at a flow rate of 12.5 μl/min at a pH of 7.5. Next, a solution of 1 M NaCl and 1 M NaOH was injected to all 16 channels for 2 min. Afterwards, 100 μl of a mixture of 200 mM EDC and 100 mM NHS was added. For coating of the streptavidin, an acetate buffer (10 mM sodium acetate) at a pH of 5.5 was used and a 100 μl injection resulted in an approx. response of 2500 RU. The running buffer was switched to a HEPES buffer (10 mM HEPES at pH 7.4, 200 mM NaCl, 3.4 mM EDTA, 0.01 % (v/v) Tween 20) before capturing the analyte. Approximately 10 μl of a 75 nM solution of biotinylated FOX_RRM_ in HEPES buffer was injected only on the second channel resulting in a response of approx. 200 RU. The amount of the injected ligand varied depending on the desired coating. 100 μl of the analyte was injected at a flow rate of 25 μl/min with a dissociation time of 480 s. For regeneration, 50 μl of a 2 M NaCl solution was used. After every injection, a buffer injection was added for double referencing. The binding affinities were determined from kinetic measurements or using steady-state measurements.

### Cross-linking and gel electrophoresis

Complexes of FOX_RRM_ and the desired RNA were prepared by mixing both components in equimolar ratios at the desired concentration of 10 µM in 10 mM sodium phosphate (pH = 6.5) and 50 mM NaCl and incubated for 10 min on ice. 15 μl of the sample solutions were placed in a 96 well-plate on ice and irradiated at 800 mJ/cm^2^, 1600 mJ/cm^2^, 2400 mJ/cm^2^ and 3200 mJ/cm^2^ at 254 nm in a CL-1000 Ultraviolet Crosslinker (UVP, Cambridge). The samples were then loaded on a 4-20% Tris-Glycine SDS-Gel with a 1xTris/Glycine/SDS running buffer. The gels were stained using the Pierce Silver Stain Kit and uncropped pictures of the gels can be found in the Supplementary Information.

### Cross-linking and mass spectrometry

75 µg of RNA-protein complexes were made of equimolar mixtures of unlabelled and ^13^C-labelled RNA and irradiated four times with 800 mJ/cm2 as described above. Each irradiation step was separated by 1 min for sample cooling. After irradiation, samples were precipitated with 3 volumes of ethanol at −20 °C and 1/10 volumes 3 M sodium acetate (pH 5.2), left at −20 °C for at least 2 h, and centrifuged at 4 °C for 30 min at 13,000 × *g*. Resulting pellets were washed by brief vortexing in 80% ethanol at −20 °C, and centrifugation was repeated. For the digestion with alkaline hydrolysis: Pellets were air dried for 10 min, then were resuspended in 50 μl of 50 mM Tris-HCl (pH 7.9). 1 ml 0.1 M NaOH was added, and the sample incubated at 70 °C for 10 min on a shaking incubator. The sample was neutralized with 105 μl 1 M HCl, cooled on ice, purified using solid-phase extraction and evaporated to dryness in a vacuum centrifuge. The sample was resuspended in 50 μl 50 mM Tris-HCl, pH 7.9, 4 M urea and then diluted with 150 μl 50 mM Tris-HCl, pH 7.9. The exact procedures of the digestion using RNases and trypsin, the enrichment using titanium dioxide affinity chromatography, and LC-MS analysis^28^ are described in the Supplementary Information. All identified cross-links are listed in Supporting Data 2 and the masses of the RNA adducts and neutral mass losses are given in Supporting Data 3.

### Reporting summary

Further information on research design is available in the Nature Research Reporting Summary linked to this article.

## Supplementary information

Supplementary Information

Description of Additional Supplementary Files

Supplementary Data 1

Supplementary Data 2

Supplementary Data 3

Supplementary Data 4

Reporting Summary

## Data Availability

The mass spectrometry proteomics data have been deposited at the ProteomeXchange Consortium via the PRIDE^77^ partner repository with the dataset identifier PXD031381. The referenced accession codes for the structures in the Protein Data Bank are 2ERR, 2AD9, 2ADB, 2ADC, 3TS0, 2MXY, 4V88, 2YH1, 4ZT0.
