# Supplementary material for: Nucleotide-amino acid π-stacking interactions initiate photo cross-linking in RNA-protein complexes
Source: Nat Commun. 2022 May 17;13:2719. doi: 10.1038/s41467-022-30284-w (PMC9114321; doi:10.1038/s41467-022-30284-w)
Supplement: Supplementary file 1 — Supplementary Information [file 41467_2022_30284_MOESM1_ESM.pdf]

Supporting Information

Nucleotide-amino acid π-stacking interactions initiate photo cross-linking in RNA-protein complexes.

Anna Knörlein[1], Chris P. Sarnowski[2], Tebbe de Vries[3], Moritz Stoltz[1], Michael Götze[2], Ruedi Aebersold[2,4], Frédéric H.-T. Allain[3], Alexander Leitner[2], Jonathan Hall[1]*

[1]Department of Chemistry and Applied Biosciences, Institute of Pharmaceutical Sciences, ETH Zurich, Zurich, Switzerland

[2]Department of Biology, Institute of Molecular Systems Biology, ETH Zurich, Zurich, Switzerland

[3]Department of Biology, Institute of Biochemistry, ETH Zurich, Zurich, Switzerland

[4]Faculty of Science, University of Zurich, Zurich, Switzerland

Corresponding author: jonathan.hall@pharma.ethz.ch

Table of Contents

Supplementary methods 3

Supplementary Figure 1. Chemical digestion of RNA enhances the detection of single nucleotide cross links. 6

Supplementary Figure 2. Incorporation of 13C-labelled phosphoramidite into FOX_{RBE}. 8

Supplementary Figure 3. Mutations at G_2 and G_6 attenuate cross-linking to FOX_{RRM}. 9

Supplementary Figure 4. Cross-linking is clustered around aromatic amino acids and 1-3 flanking amino acids. 10

Supplementary Figure 5. Singly-labelled FOX_{RBE} mutants cross-link to FOX_{RRM}. 11

Supplementary Figure 6. FOX_{RBE} mutants cross-link to FOX_{RRM} similarly to wild type FOX_{RBE}. 13

Supplementary Figure 7. FOX_{RRM}/FOX_{RBE} structure showing π-stacking of H120 and U_5. 14

Supplementary Figure 8. CLIR MS of PTBP1 in complex with IRES RNA of EMCV and overlay of NMR solution structure of PTBP1 in complex with CUCUCU. 16

Supplementary Figure 9. Full length gels from cross-linking reactions shown in Figures 2-3 and Supplementary Figure 3. 17

Supplementary Figure 10: LC-MS traces of synthesised oligonucleotide sequences. 68

Supplementary Table 1. Variants of the FOX_{RBE} used in this study. 69

Supplementary Data 1.. List of plotted protein-RNA cross-links (separate excel file) 70

Supplementary Data 2. List of identified protein-RNA cross-links (separate excel file) 70

Supplementary Data 3. Table of RNA seqs and neutral losses used for xQuest analyses (separate excel file) 70

Supplementary Data 4: Analysis of large-scale datasets of Kramer et al and Bae et al. (separate excel file) 70

Supplementary References 71

Supplementary methods

RNA synthesis

All chemicals were purchased from Fluorochem (Hadfield, United Kingdom), TCI (Tokio, JPN) and Aldrich (St. Louis, MO). Phosphoramidites were purchased from ThermoFisher scientific (Waltham, MA). The ^{13}C ribose-labelled phosphoramidites were purchased from Pitsch NucleicAcids (Stein am Rhein, CH).

The synthesis of all oligonucleotides was carried out with an MM12 synthesizer (Bio Automation Inc., Plano, TX) on a 50 nmol scale using 500 Å UnyLinker CPG (ChemGenes, Wilmington, MA). The coupling time for 2′-O-TBDMS phosphoramidites was 2 × 150 s and 2 × 240 s for the 2′-O-TOM phosphoramidites. The RNA phosphoramidites were prepared as 0.08 M solutions in dry acetonitrile (ACN), the activator BTT (Biosolve, Valkenswaard, Netherlands) was prepared as a 0.24 M solution in dry ACN. A 0.02 M I_2 solution in THF/Pyridine/H_2O (70:20:10, w/v/v/v) was used for oxidation. Capping was achieved by using THF/lutidine/acetic anhydride (8:1:1) and 16% N-methylimidazole/THF. Detritylations were performed using 3% dichloroacetic acid in dichloromethane. Deprotection was performed by treating the CPG with gaseous methylamine for 1.5 h at 70 °C. Afterwards, the oligonucleotides were washed from the solid support with 3 × 200 µl of H_2O/EtOH (1:1). To the solution, 20 µl of 1 M Tris buffer was added, and the solution was reduced to dryness under vacuum. Desilylation was carried out by treatment with a mixture of N-methyl-2-pyrrolidone (60 µl), triethylamine (30 µl), and triethylamine trihydrofluoride (40 µl) at 70 °C for 2 h. The reaction was quenched by adding trimethylethoxysilane (200 µl, 5 min, room temperature). After precipitation with diethyl ether (200 µl, 5 min, room temperature), the solid was dissolved in 200 µl of H_2O and purified on an Agilent 1200 series preparative RP-HPLC on an XBridge OST C-18 column (10 × 50 mm, 2.5 µm; Waters, Milford, MA) at 65 °C with a flow rate 5 ml/min. Buffer A: 0.1 M aqueous triethylamine/acetic acid, pH 8.0; buffer B: 100% ACN; gradient 10–50% B in 5 min.

Fractions containing the DMT-on product were collected, dried under vacuum, and treated with 40% aqueous acetic acid for 15 min at room temperature. Samples were dried under vacuum and dissolved in 200 µl of H_2O, and purified in the DMT-off mode by RP-HPLC on an XBridge OST C-18 column (10 × 50 mm, 2.5 µm; Waters, Milford, MA) at 65 °C with a flow rate 5 ml/min. Buffer A: 0.1 M aqueous triethylamine/acetic acid, pH 8.0; buffer B: 100% ACN; gradient 2–20% B in 15 min.

Fractions containing the desired product were collected and dried under reduced pressure. The oligonucleotides were analyzed by LC–MS (Agilent 1200/6130 system) on an Acquity OST C-18 column (2.1 × 50 mm; Waters, Milford, MA). The column oven was set to 65 °C, flow-rate: 0.3 ml/min. Solvent A: H_2O containing 0.4 M hexafluoroisopropanol, 15 mM triethylamine; solvent B: MeOH; gradient 1–35% B in 15 min. UV absorption of the final products was measured on a NanoDrop 2000 spectrophotometer (ThermoFisher scientific, Waltham, MA).

Cross-linking and mass spectrometry

For the digestion using RNases, 75 µg of RNA-protein complexes were made from equimolar mixtures of unlabelled and isotope-labelled RNA and irradiated four times at 800 mJ/cm^2. Each irradiation step was separated by 1 min for sample cooling. After irradiation, samples were precipitated with 3 volumes of ethanol at -20°C and 1/10 volumes 3 M sodium acetate (pH 5.2), left at -20°C for at least 2 h, and centrifuged at 4°C for 30 min at 13,000 g. Resulting pellets were washed by brief vortexing in 80% ethanol at -20°C, and centrifuged repeatedly. Pellets were air-dried for 10 min, then were resuspended in 50 µL of 50 mM Tris-HCl (pH 7.9) with 4 M urea, and then diluted with 150 µL 50 mM Tris-HCl, pH 7.9. RNases A (Roche Diagnostics, Rotkreuz, CH) and T1 (ThermoScientific, Waltham, MA) were added at 5 µg or 5 U per mg of cross-linked sample, respectively. Samples were digested for 2 h at 52 °C. Samples were cooled on ice, and 2 µl of 1 M $MgCl_2$ was added to each sample, followed by 125 U of

benzonase (Sigma Aldrich, St Louis, MO) per mg of cross-linked complex. Samples were further digested for 1 h at 37 °C.

Trypsin was added in a 24:1 protein:enzyme ratio (w/w) to all samples, and the samples incubated overnight at 37°C on a shaking incubator. Samples were heated to 70°C for 10 min to deactivate trypsin, purified by solid-phase extraction (SPE, Waters SepPak 50 mg tC18 cartridges, Milford, MA), and evaporated to dryness in a vacuum centrifuge. RNA–protein crosslinks were enriched by titanium dioxide affinity chromatography, as described previously[1].

Briefly, dried samples were resuspended in 100 µl of 50% acetonitrile, 0.1% trifluoroacetic acid, 10 mM lactic acid (loading buffer). The samples were incubated for 30 min on a shaking incubator at >10,000 rpm with 5 mg of pre-equilibrated TiO_2 beads (10 µm Titansphere PhosTiO, GL Sciences, Tokyo, JPN), and the beads settled by centrifugation. The supernatant was removed and replaced with 100 µl fresh loading buffer, and the sample incubated for a further 15 min. Centrifugation was repeated, the supernatant removed, and 100 µl 50% acetonitrile, 0.1% trifluoroacetic acid (washing buffer) was added, followed by 15 min incubation, centrifugation for 1 min at 10,000 g, and removal of the supernatant. Peptide-RNA adducts were eluted from the beads with 50 µl 50 mM ammonium phosphate, pH 10.5, and incubated for 15 min on a shaking incubator at >1000 rpm. Beads were settled by centrifugation as in previous steps, and the supernatant was carefully collected. The elution step was repeated a second time, and the eluate was stored on ice. The eluate was immediately acidified to pH 2-3 with TFA, and purified with solid phase extraction using self-packed Stage tips. Briefly, two layers of C18-filter (Empore, 3M, ThermoFisher scientific, Waltham, MA) in a 200 µl tip were washed with 100% acetonitrile, 80% acetonitrile with 0.1% formic acid, then twice with 5% acetonitrile with 0.1% formic acid. The sample was applied, and the tips then washed 3 times using 5% acetonitrile with 0.1% formic acid, and finally eluted 3 times using 50% acetonitrile with 0.1% formic acid. Pre-washed LoBind tubes (Eppendorf, Hamburg, DE) were used for collection of eluate, and subsequent evaporation to dryness.

Dried samples were resuspended in 20 µl 5% acetonitrile with 0.1% formic acid, and 5 µl sample was injected for LC-MS/MS analysis. For all experiments except those shown in Fig. S1, LC-MS/MS analysis was performed with an Easy-nLC 1200 HPLC system (ThermoFisher scientific, Waltham, MA) connected to an Orbitrap Fusion Lumos mass spectrometer (ThermoFisher scientific, Waltham, MA) with a Nanoflex (ThermoFisher scientific, Waltham, MA) nanoflow electrospray source. Separation of peptide-RNA adducts took place on a PepMap RSLC column (150 mm x 75 µm, 2 µm particle size, ThermoFisher scientific, Waltham, MA) using a gradient of 6-40% mobile phase B (A = water:acetonitrile:formic acid, 98:2:0.15 (v/v/v); B = water:acetonitrile:formic acid, 20:80:0.15 (v/v/v)) over 60 min with a flow rate of 300 nl/min. The Orbitrap Fusion Lumos was operated in data-dependent acquisition mode. The Orbitrap analyzer was used for acquisition of precursor ion spectra with a resolution of 120000. Higher-energy collisional dissociation (HCD) with stepped collision energies of 21.85%, 23% and 24.15% was used for fragmentation. Ions with charge states of +2 to +7 were selected for MS2 with the quadrupole mass analyzer using an isolation window of 1.2 m/z and a cycle time of 3 s. The dynamic exclusion period was set to 30 s. Fragment ions were detected in the ion trap, or in the Orbitrap with a resolution of 30000.

For data shown in Fig. S1, LC-MS/MS analysis was performed with an Easy-nLC 1000 HPLC system (ThermoFisher scientific, Waltham, MA) connected to an Orbitrap Elite mass spectrometer (ThermoFisher scientific, Waltham, MA) with a Nanoflex (ThermoFisher scientific, Waltham, MA) nanoflow electrospray source. Separation of peptide-RNA adducts took place on a PepMap RSLC column (150 mm x 75 µm, 2 µm particle size, ThermoFisher

scientific, Waltham, MA) using a gradient of 5-30% mobile phase B (A = water:acetonitrile:formic acid, 98:2:0.15 (v/v/v); B = water:acetonitrile:formic acid, 2:98:0.15 (v/v/v)) over 60 min with a flow rate of 300 nl/min.

The Orbitrap Elite was operated in data-dependent acquisition mode. The Orbitrap analyzer was used for acquisition of precursor ion spectra with a resolution of 120000. Precursor ions were fragmented with collision-induced dissociation (CID, normalised collision energy = 35%), with dynamic exclusion enabled for 30 sec. Fragment ions were detected at "Normal" resolution in the ion trap.

ThermoFisher RAW data files produced by the mass spectrometer were converted to centroided mzXML files with msconvert.exe (ProteoWizard version 3.0.9393) and searched against a FASTA database containing the FOX_{RRM} protein sequence using xQuest.[2] All amino acids were specified as possible modification sites, and all possible adducts of 1-4 nucleotides in length, based on the sequence UGCAUGU or the subsequent mutated sequences, were considered possible modifications (Supplementary Data 3). A mass shift of 5.016774 Da per labelled nucleotide in the expected sequence was specified, in order to obtain only identifications from labelled RNA. A mass tolerance window of +/- 15 ppm and retention time tolerance of 60 s was used for light-heavy species pairing. Additional xQuest search parameters (described in Walzthoeni *et al* 2012[2]): Enzyme = trypsin, maximum missed cleavages = 2, MS1 mass tolerance = 10 ppm, MS2 mass tolerance = 0.2 Da. Only identifications with an Id.Score > 20 (according to the scoring scheme of Walzthoeni et al. [2]) were considered. The Qutputs from xQuest analysis were plotted using the Plotly package (4.6.0) in Python 3.7. The mass spectrometry proteomics data have been deposited at the ProteomeXchange Consortium via the PRIDE[3] partner repository with the dataset identifier PXD031381. All identified cross-links are listed in Supporting Data 2.

Where plots are shown with "Number of cross-linked RNA adducts", the number refers to the number of cross-link spectrum matches from a given LC-MS/MS dataset.

Unbiased analysis of three large cross-linking datasets

For the analysis of the cross-linking data from Kramer et al.[4], the human and yeast dataset was manually filtered for non-sulphur containing cross-linking sites (Supporting Data 4). Next, cross-linked amino acids that are aromatic (F, W, Y, H) or within -/+3 amino acids of an aromatic amino acid were selected. For these, the Protein Data Bank (PDB) was searched for high resolution structures using the UniProt number and the assigned cross-linked amino acid or its neigbouring aromatic amino acid was manually evaluated for possible participation in a π-stacking interaction. In case of unavailable structures also closely related protein structures were considered. Protein hits with no known structure (Date: January 1, 2022) or hits where the cross-linked amino acid is not in contact with the RNA were designated as "unknown".

For the analysis of the cross-linking data from Bae et al.[5], the data was filtered for hits with a total peptide-spectrum match (PSM) Count >3. Next, cross-linked amino acids that are aromatic (F, W, Y, H) or within -/+3 amino acids neigbouring an aromatic amino acid were manually evaluated for π-stacking interaction using the crystal structure (PDB ID: 4ZT0). Cross-linked amino acids which are not in contact with the RNA molecule in the structure were designated as "unknown".

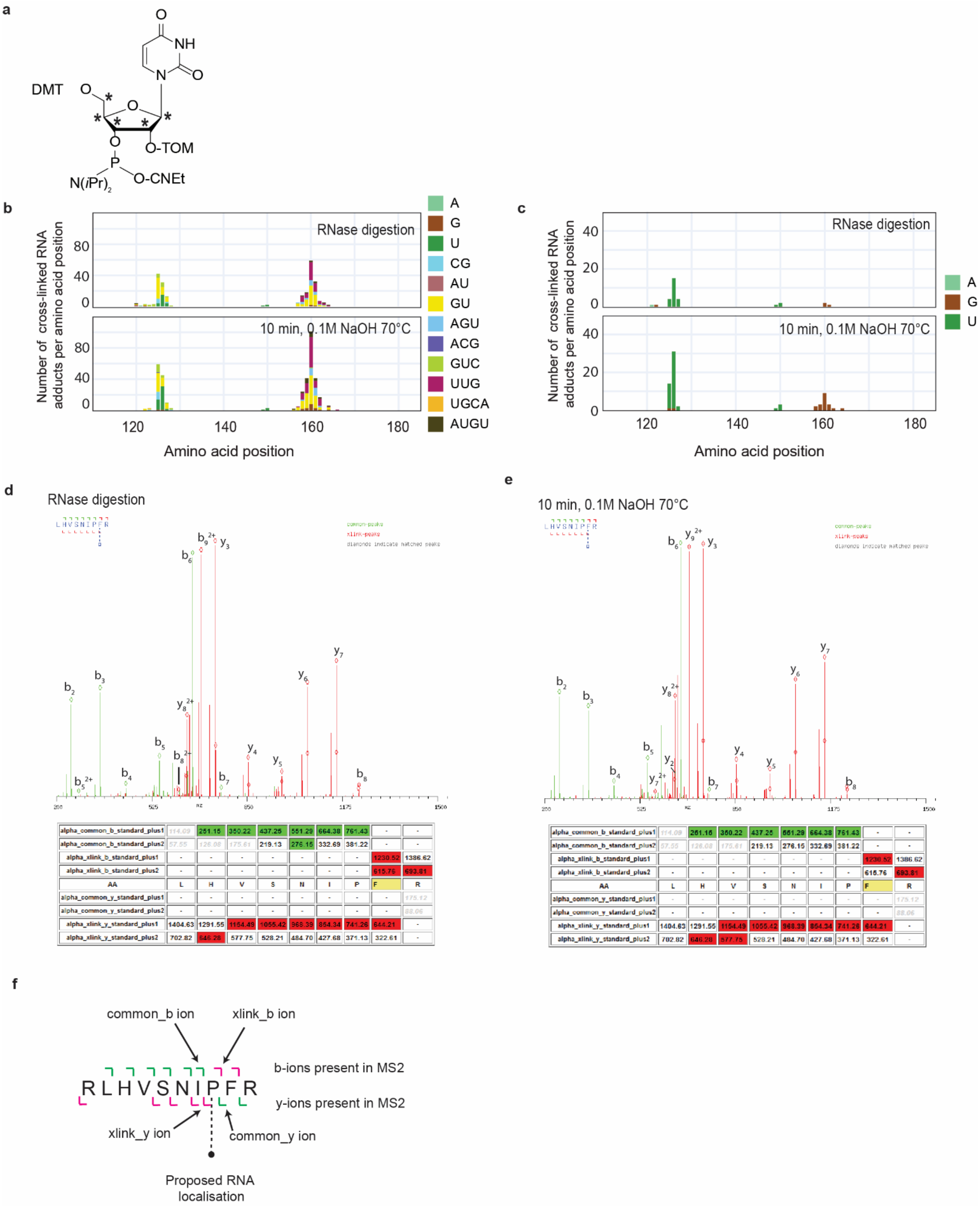

Supplementary Figure 1. Chemical digestion of RNA enhances the detection of single nucleotide cross links.

a) Structure of ^{13}C-labelled 5'-dimethoxytrityl-uridine,2'-O-triisopropylsilyloxymethyl-3'-[(2-cyanoethyl)-(N,N-diisopropyl)]-phosphoramidite; U, C, A and G derivatives were used for synthesis (asterisks indicate the position of the ^{13}C atoms). **b)** CLIR-MS plots of uniformly labelled FOX_{RBE} cross-linked to FOX_{RRM}. The digestion was

performed according to standard CLIR-MS protocol[1] with RNase A, RNase T1 and Benzonase (upper panel) and *via* alkaline hydrolysis (10 min, 0.1 M NaOH, at 70°C) (lower panel). The xQuest software was used to search for cross-linked mono-, di-, tri- and tetra-nucleotide adducts that are present in FOX_{RBE} which are colour coded. **c)** CLIR MS plot showing mononucleotides (color coded) from the data in b). A larger number of cross-linked mononucleotides are detected using alkaline hydrolysis. **d)** xQuest MS/MS (manually annotated) spectra of uniformly labelled FOX_{RBE} cross-linked to $FOX_{RRM,}$ digested with with RNase A, RNase T1 and Benzonase. **e)** xQuest MS/MS (manually annotated) spectra of uniformly labelled FOX_{RBE} cross-linked to $FOX_{RRM,}$ digested *via* alkaline hydrolysis. For manually annotated spectra, the common peptides are labelled in green, while the cross-linked peptides are marked in red. **f)** Illustration of peptide fragment ion positions relative to a proposed RNA cross-linking site. The peptide fragments which do not contain the RNA adduct (common ions) are labelled in green while the peptide fragments containing the cross-linked RNA adduct (xlink ions) are labelled in red.

a

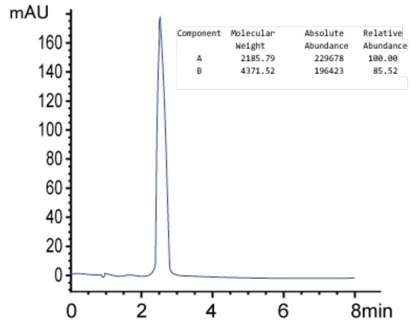

b

Sequence	$Mass_{calc}$	$Mass_{found}$	UV Purity
*UGCAUGU	2186.4	2185.8	>99%
U*GCAUGU	2186.4	2185.7	>99%
UG*CAUGU	2186.4	2185.8	>99%
UGC*AUGU	2186.4	2185.8	>99%
UGCA*UGU	2186.4	2185.8	>99%
UGCAU*GU	2186.4	2185.8	>99%
UGCAUG*U	2186.4	2185.8	>99%
UGCAUGU	2181.4	2180.9	>99%

c

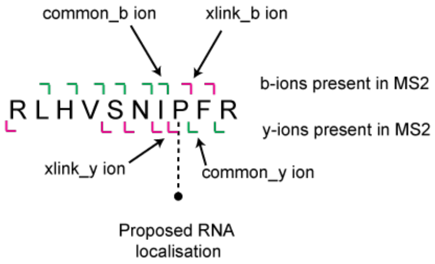

d

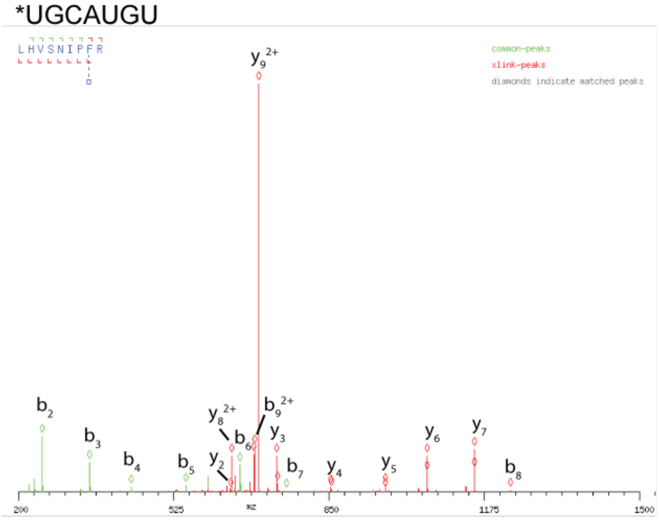

alpha_common_b_standard_plus1	114.09	251.15	350.22	437.25	551.29	664.38	761.43	-	-
alpha_common_b_standard_plus2	57.55	126.08	175.61	219.13	276.15	332.69	381.22	-	-
alpha_xlink_b_standard_plus1	-	-	-	-	-	-	-	1230.52	1386.62
alpha_xlink_b_standard_plus2	-	-	-	-	-	-	-	615.76	693.81
AA	L	H	V	S	N	I	P	F	R
alpha_common_y_standard_plus1	-	-	-	-	-	-	-	-	175.12
alpha_common_y_standard_plus2	-	-	-	-	-	-	-	-	88.06
alpha_xlink_y_standard_plus1	1404.63	1291.55	1154.49	1055.42	968.39	854.34	741.26	644.21	-
alpha_xlink_y_standard_plus2	702.82	646.28	577.75	528.21	484.70	427.68	371.13	322.61	-

e

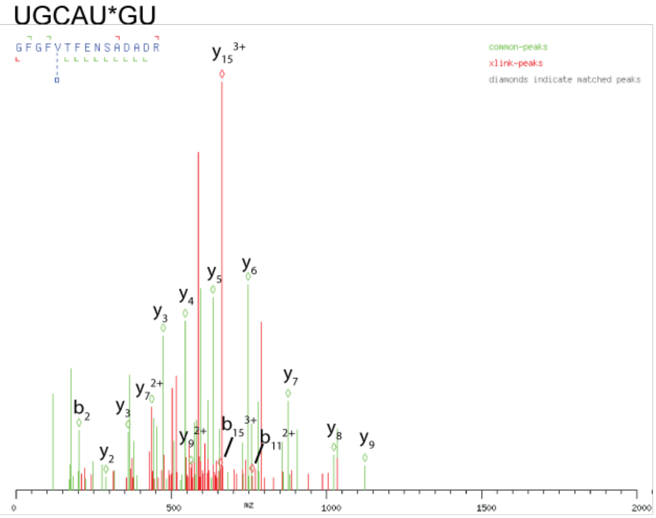

alpha_common_b_standard_plus1	58.03	205.10	262.12	409.19	-	-	-	-	-	-	-	-	-	-	-
alpha_common_b_standard_plus2	29.52	103.05	131.56	205.10	-	-	-	-	-	-	-	-	-	-	-
alpha_common_b_standard_plus3	20.01	69.04	88.04	137.07	-	-	-	-	-	-	-	-	-	-	-
alpha_xlink_b_standard_plus1	-	-	-	-	869.30	970.35	1117.41	1246.46	1360.50	1447.53	1518.57	1633.60	1704.63	1819.66	1975.76
alpha_xlink_b_standard_plus2	-	-	-	-	435.15	485.68	559.21	623.73	680.75	724.27	759.79	817.30	852.82	910.33	988.38
alpha_xlink_b_standard_plus3	-	-	-	-	290.44	324.12	373.14	416.16	454.17	483.18	506.86	545.20	568.88	607.23	659.26
AA	G	F	G	F	V	T	F	E	N	S	A	D	A	D	R
alpha_common_y_standard_plus1	-	-	-	-	-	1125.48	1024.43	877.37	748.32	634.28	547.25	476.21	361.18	290.15	175.12
alpha_common_y_standard_plus2	-	-	-	-	-	563.24	512.72	439.19	374.67	317.64	274.13	238.61	181.10	145.58	88.06
alpha_common_y_standard_plus3	-	-	-	-	-	375.83	342.15	293.13	250.11	212.10	183.09	159.41	121.07	97.39	59.05
alpha_xlink_y_standard_plus1	1993.77	1936.75	1789.68	1732.66	1585.59	-	-	-	-	-	-	-	-	-	-
alpha_xlink_y_standard_plus2	997.39	968.88	895.34	866.83	793.30	-	-	-	-	-	-	-	-	-	-
alpha_xlink_y_standard_plus3	665.26	646.26	597.23	578.23	529.20	-	-	-	-	-	-	-	-	-	-

Supplementary Figure 2. Incorporation of 13C-labelled phosphoramidite into FOX_{RBE}.

a) UV-trace from a high-performance liquid chromatography (HPLC) chromatogram of *UGCAUGU. **b)** ^{13}C-labelled FOX_{RBE} versions used in this study (*N indicates a ^{13}C-labelled nucleotide). **c)** Illustration of peptide fragment ion positions relative to a proposed RNA cross-linking site. The peptide fragments which do not contain the RNA adduct (common ions) are labelled in green while the peptide fragments containing the cross-linked RNA adduct (xlink ions) are labelled in red. **d)** xQuest MS/MS (mannually annotated) spectra of singly labelled *UGCAUGU cross-linked to FOX_{RRM}, **e)** xQuest MS/MS (mannually annotated) spectra of singly labelled UGCAU*GU cross-linked to FOX_{RRM},. For manually annotated spectra, the common peptides are labelled in green, while the cross-linked peptides are marked in red.

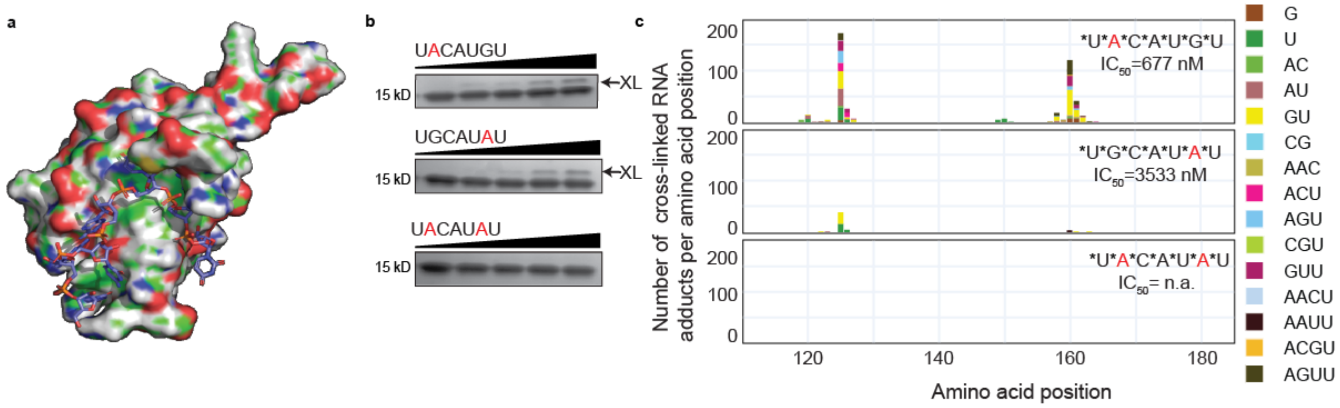

Supplementary Figure 3. Mutations at G_2 and G_6 attenuate cross-linking to FOX_{RRM}.

a) Overview of the solution structure of FOX_{RRM} complexed with UGCAUGU (PDB ID: 2ERR)[6]. Surface (heavy atoms of RRM) and stick (heavy atoms of the RNA) represent the lowest energy structure (structure visualized with PyMOL (PyMOL Molecular Graphics System, Version 2.5 Schrödinger, LLC)). **b)** SDS-PAGE showing that G_2/G_6 mono-mutants undergo cross-linking with FOX_{RRM} with increasing irradiation. The cross-linking band is indicated by XL (repeated three times). **c)** CLIR-MS plots of uniformly labelled G_2/G_6 mutants cross-linked to FOX_{RRM}, in order to identify sites of cross-linking; the xQuest software was used to search for cross-linked mono-, di-, tri- and tetra-nucleotide adducts which are colour coded. Mutation of G_2 or G_6 to A_2 or A_6 greatly attenuates cross-linking of the mutated nucleotides to the clusters of amino-acids around positions 126 and 160, respectively. Inserted IC_{50} values are taken from Auweter *et al.*[6] and show that mutation of G_2 and G_6 attenuates binding to FOX_{RRM} (the mutated nucleotide is labelled in red).

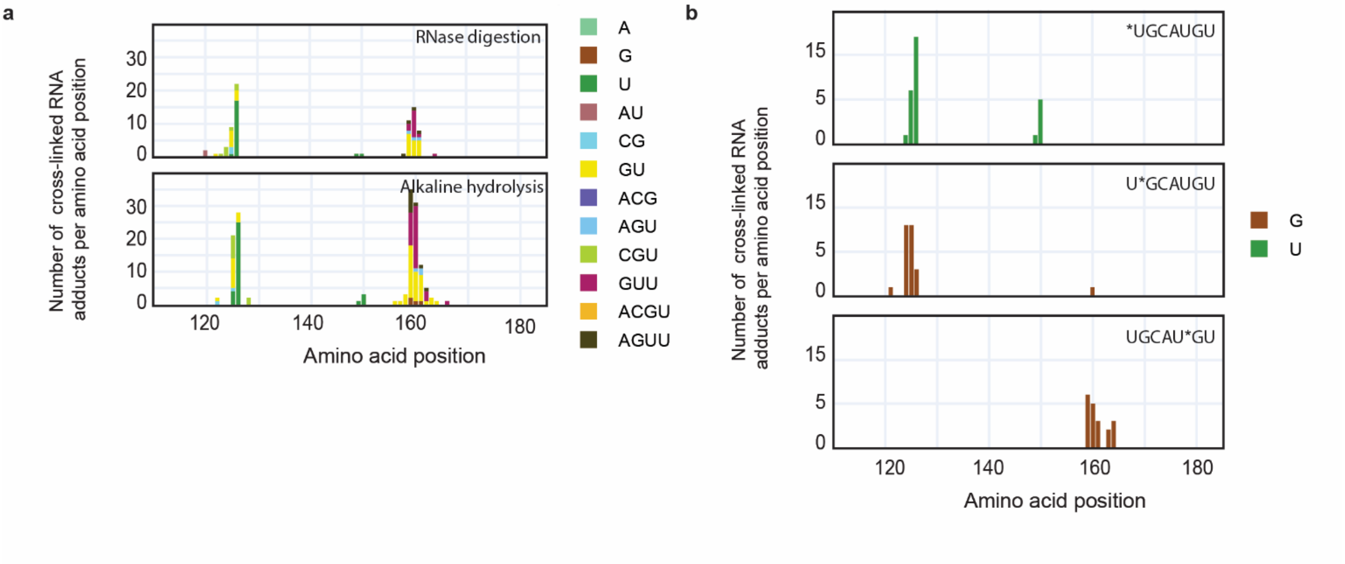

Supplementary Figure 4. Cross-linking is clustered around aromatic amino acids and 1-3 flanking amino acids. .

a) Data from Fig. S1 was filtered for CLIR-MS identifications where the MS2 spectrum contains fragment ions that unambiguously localise the RNA modification to a single amino acid position. **b)** Data from Fig 1c (sequences of *UGCAUGU, U*GCAUGU and UGCAU*GU) were filtered for CLIR-MS identifications where the MS2 spectrum contains fragment ions that unambiguously localise the RNA modification to a single amino acid position. Any pair of common or xlink and b or y- ions that fall on opposing sides of the proposed cross-linked amino acid imply unambiguous localisation (see highlighted fragments in Fig S1f) (*N indicates a ^{13}C-labelled nucleotide).

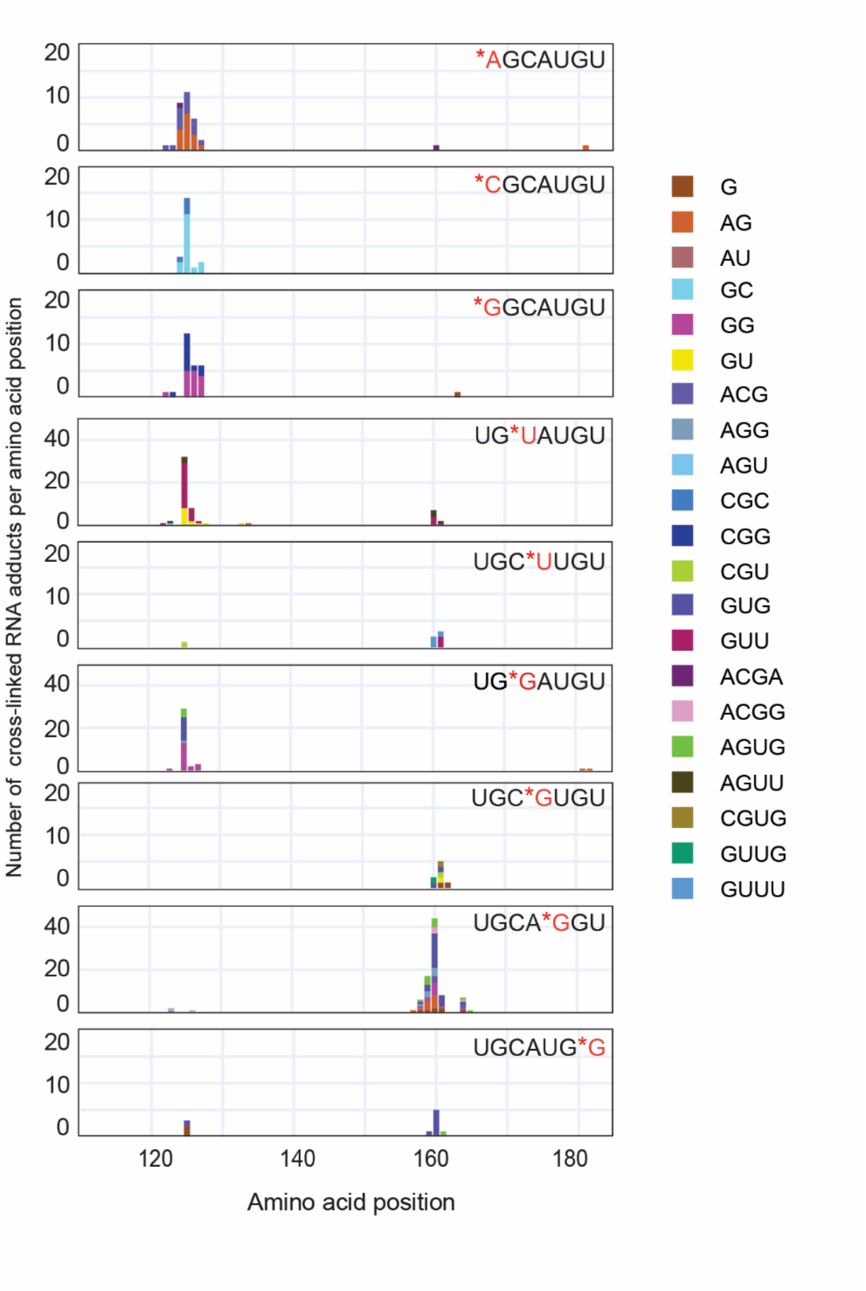

Supplementary Figure 5. Singly-labelled FOX_{RBE} mutants cross-link to FOX_{RRM}.

CLIR-MS analysis of singly-labelled FOX_{RBE} mutants to FOX_{RRM} using alkaline hydrolysis work-up. Plots show number of RNA adducts at each amino acid position of FOX_{RRM}. The xQuest software was used to search for cross-linked mono-, di-, tri- and tetra-nucleotide adducts that include a labelled nucleotide which are colour coded (*N indicates a ^{13}C-labelled nucleotide and the mutated nucelotide is labelled in red).

a

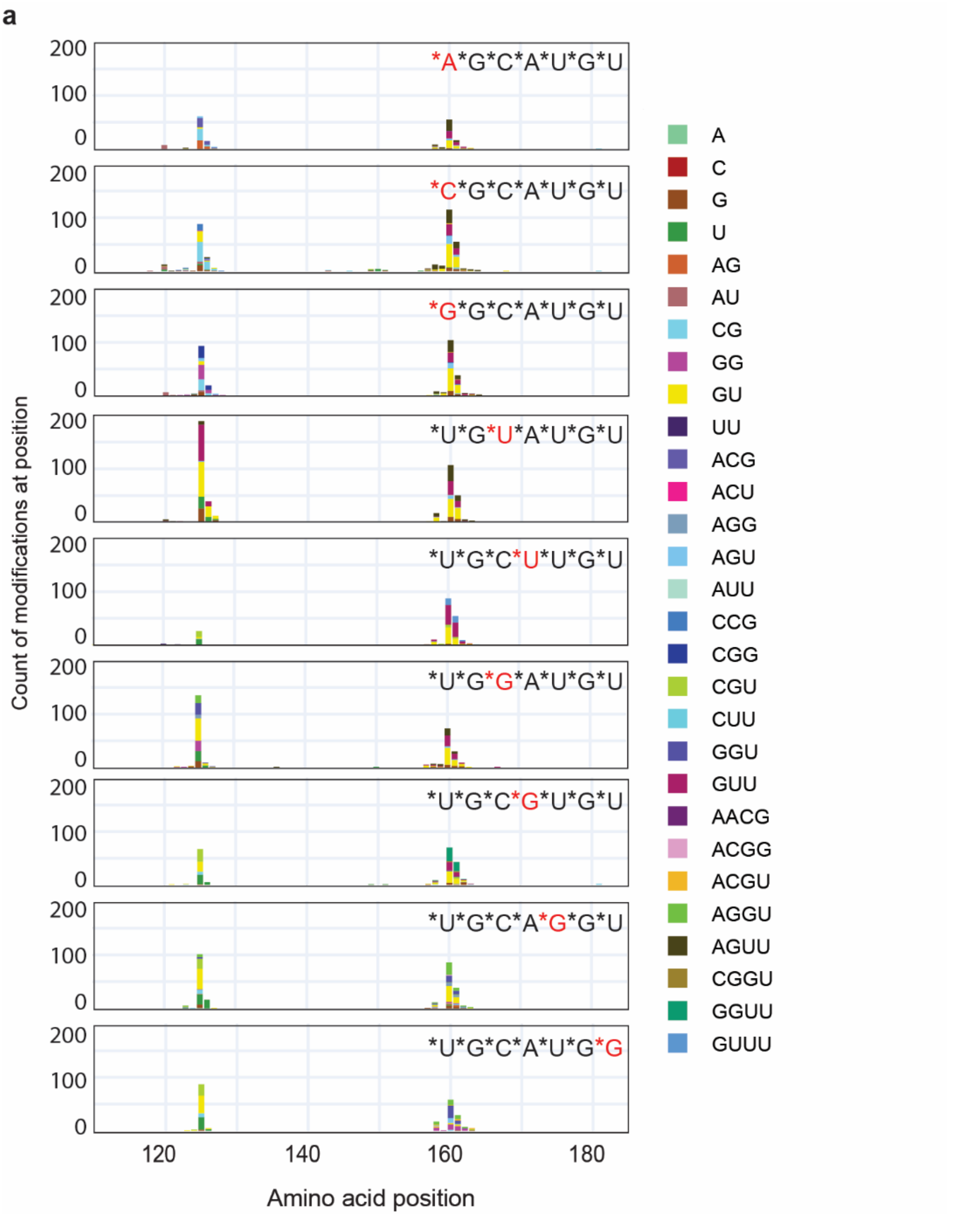

200
100
0
*A*G*C*A*U*G*U
*C*G*C*A*U*G*U
*G*G*C*A*U*G*U
*U*G*U*A*U*G*U
*U*G*C*U*U*G*U
*U*G*G*A*U*G*U
*U*G*C*G*U*G*U
*U*G*C*A*G*G*U
*U*G*C*A*U*G*G
Count of modifications at position
120
140
160
180
Amino acid position
A
C
G
U
AG
AU
CG
GG
GU
UU
ACG
ACU
AGG
AGU
AUU
CCG
CGG
CGU
CUU
GGU
GUU
AACG
ACGG
ACGU
AGGU
AGUU
CGGU
GGUU
GUUU

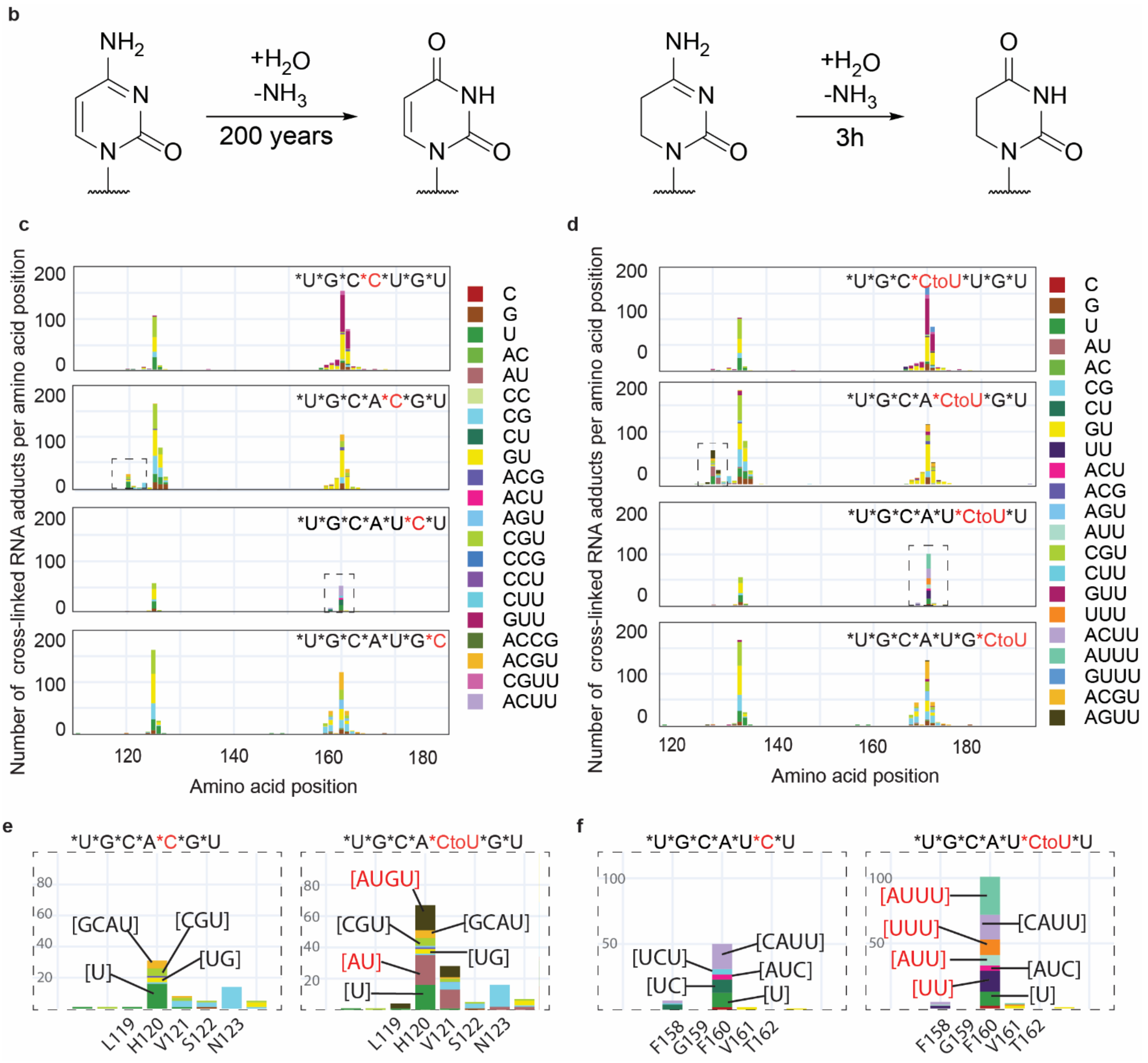

Supplementary Figure 6. FOX_{RBE} mutants cross-link to FOX_{RRM} similarly to wild type FOX_{RBE}.

a) CLIR-MS analysis of uniformly-labelled FOX_{RBE} mutants after cross-linking with FOX_{RRM}. Plots show number of RNA adducts at each amino acid position; the RNase digestion, enrichment, and LC-MS/MS analysis for these experiments were performed according to the standard CLIR MS protocol[1] and the xQuest software searched for all cross-linked mono-, di-, tri- and tetra-nucleotide adducts present in each FOX_{RBE} mutant (*N indicates a ^{13}C-labelled nucleotide and mutated nucelotides are labelled in red). **b)** Rates of spontaneous hydrolysis of cytidine[7] and 5-6 dihydrocytidine according to literature[8]. **c)** CLIR-MS analysis was performed for four uniformally labelled FOX_{RBE} variants UGC<u>C</u>UGU, UGCA<u>C</u>GU, UGCAU<u>C</u>U, UGCAUG<u>C</u>. Plots show the number of cross-linked RNA adducts at each position of FOX_{RRM} identified by xQuest, which detects all cross-linked mono-, di-, tri- and tetra-nucleotide adducts that are present in the four FOX_{RBE} mutants. The RNase digestion, enrichment, and LC-MS/MS analysis for these experiments were performed according to the standard CLIR MS protocol[1]. **d)** The samples from c) were analyzed for evidence of hydrolysis. Here, xQuest was instructed to search for mono-, di-, tri-, and tetra-nucleotide adducts, in which the mutant cytidine is defined as a uridine (*CtoU*) (i.e. in UGC"*CtoU*"UGU, UGCA"*CtoU*"GU, UGCAU"*CtoU*"U, UGCAUG"*CtoU*"). Note the large increase in the numbers of cross-links detected around H120 and F160 in the boxed areas in the second and third panels. **e)** Expanded view of cross-links around H120 for UGCA<u>C</u>GU (left) and for the *CtoU* sequence UGCA"*CtoU*"GU (right); red-highlighted sub-sequences (AU and AUGU) likely result from hydrolysis of cytidine stacked at H120. **f)** Expanded view of cross-links around F160 for UGCAU<u>C</u>U (left) and the CtoU sequence UGCAU"*CtoU*"U (right); red-highlighted sub-sequences (UU, AUU, UUU, AUUU) likely result from hydrolysis of cytidine stacked H160, respectively.

(The RNase digestion, enrichment, and LC-MS/MS analysis for these experiments were performed according to the standard CLIR MS protocol[1]. Annotations are only performed for cross-links of >3 hits).

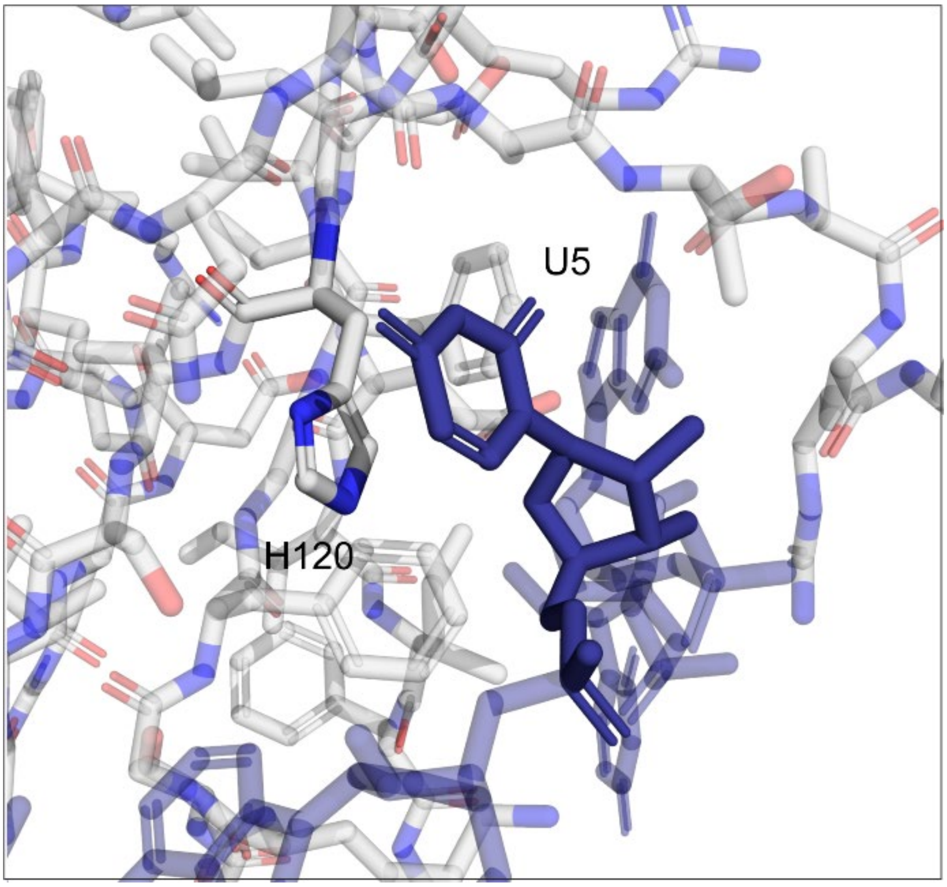

Supplementary Figure 7. FOX_{RRM}/FOX_{RBE} structure showing π-stacking of H120 and U_5.
Structure of FOX_{RRM} bound to FOX_{RBE}, visualized with PyMOL (PyMOL Molecular Graphics System, Version 2.5 Schrödinger, LLC) (from Auweter et al.[6]).

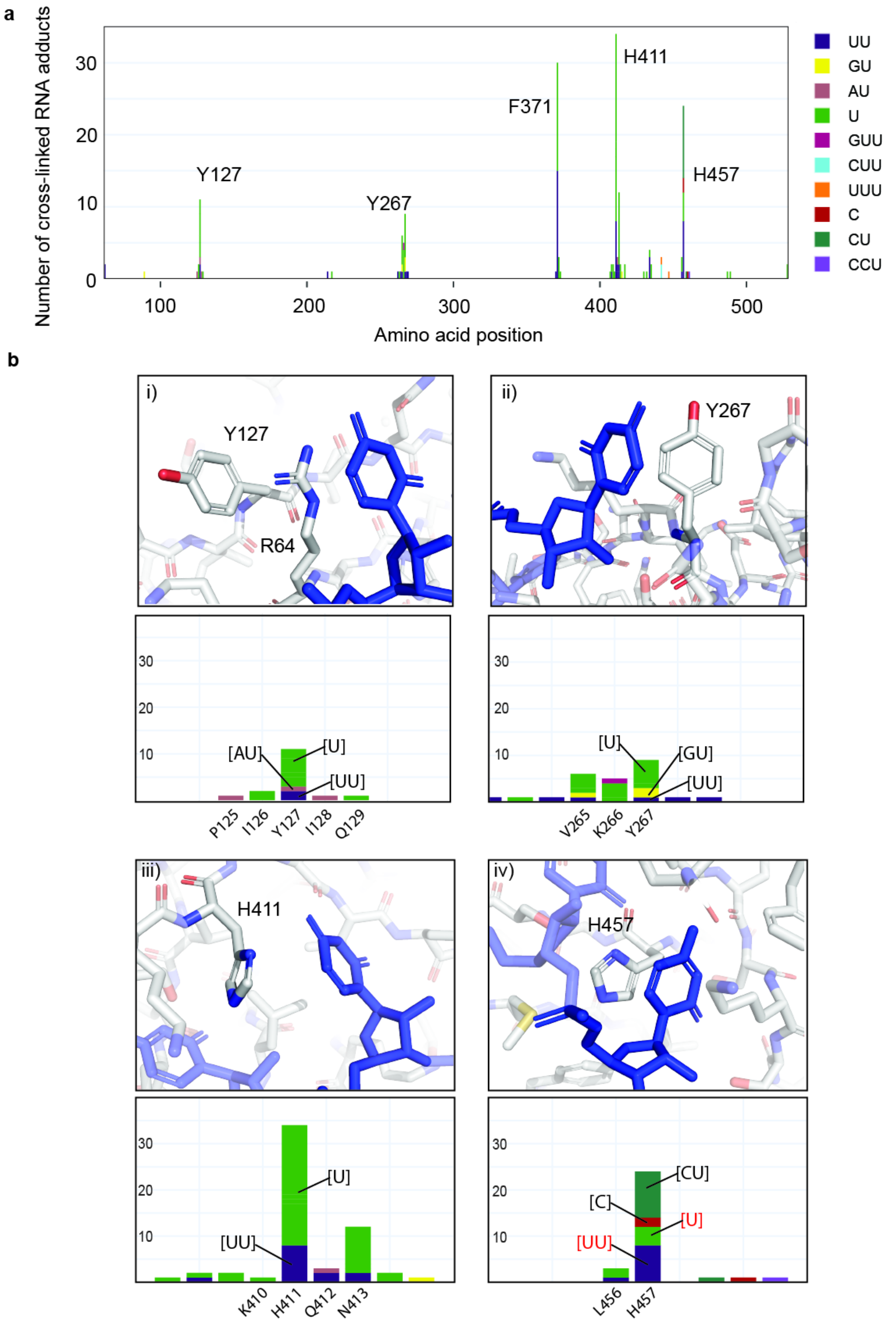
a
Number of cross-linked RNA adducts
Amino acid position
Y127
Y267
F371
H411
H457
UU
GU
AU
U
GUU
CUU
UUU
C
CU
CCU
b
i)
Y127
R64
[AU]
[U]
[UU]
P125
I126
Y127
I128
Q129
ii)
Y267
[U]
[GU]
[UU]
V265
K266
Y267
iii)
H411
[U]
[UU]
K410
H411
Q412
N413
iv)
H457
[CU]
[C]
[U]
[UU]
L456
H457

Supplementary Figure 8. CLIR MS of PTBP1 in complex with IRES RNA of EMCV and overlay of NMR solution structure of PTBP1 in complex with CUCUCU.

a) CLIR-MS analysis of PTBP1 in complex with the internal ribosomal entry site (IRES) of encephalomyocarditis virus (EMCV) analysed by Dorn *et al*.[1]. Samples were digested using RNases (Rnase A, T1 and benzonase). Plots show the number of RNA adducts at each amino acid position. The different cross-linked mono-, di-, tri- and tetra-nucleotide adducts that are present in the IRES of EMCV are colour coded. The main cross-linked amino acids are annotated.

b) Enlarged view of selected cross-linking clusters of PTBP1 in complex with IRES RNA of EMCV (same data as **Fig. S8a,** analysed by Dorn et al.[1]) with annotated cross-linking adducts correlated with NMR solution structure of PTBP1 in complex with RNA binding motif CUCUCU (PDB ID: 2AD9, 2ADB, 2ADC)[9]. (Due to the more complex structure of the IRES EMCV RNA, the cross-linking adducts UU, GU and AU are detected, which are not present in the consensus RNA binding motif CUCUCU); red-highlighted sub-sequences likely result from hydrolysis of cytidine stacked to H457. Structures are visualized with PyMOL (PyMOL Molecular Graphics System, Version 2.5 Schrödinger, LLC). The π-stack of Y127 and uridine (i) has an intervening π-stacked arginine.

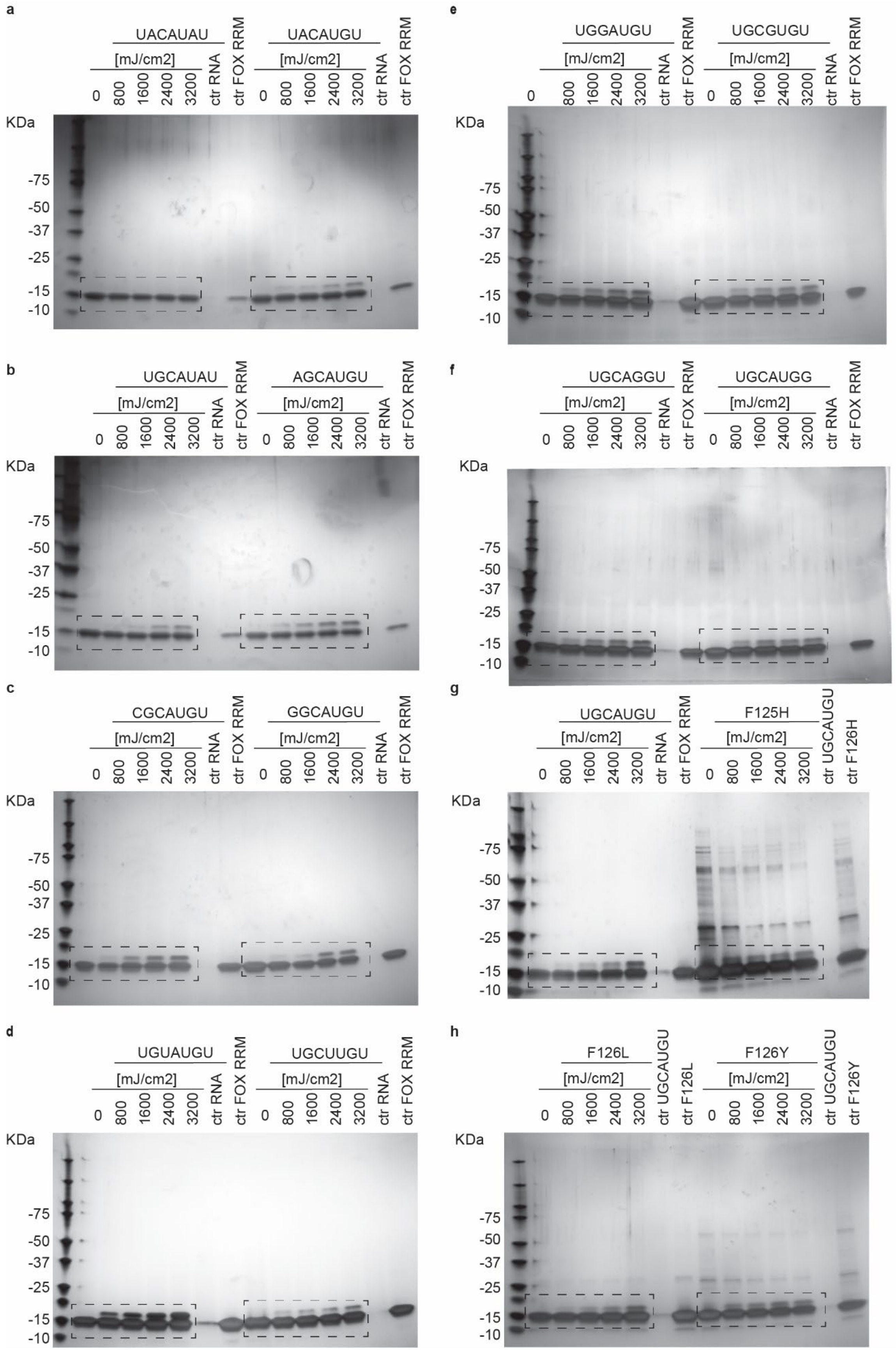

Supplementary Figure 9. Full length gels from cross-linking reactions shown in Figures 2-3 and Supplementary Figure 3.

UGCAUGU

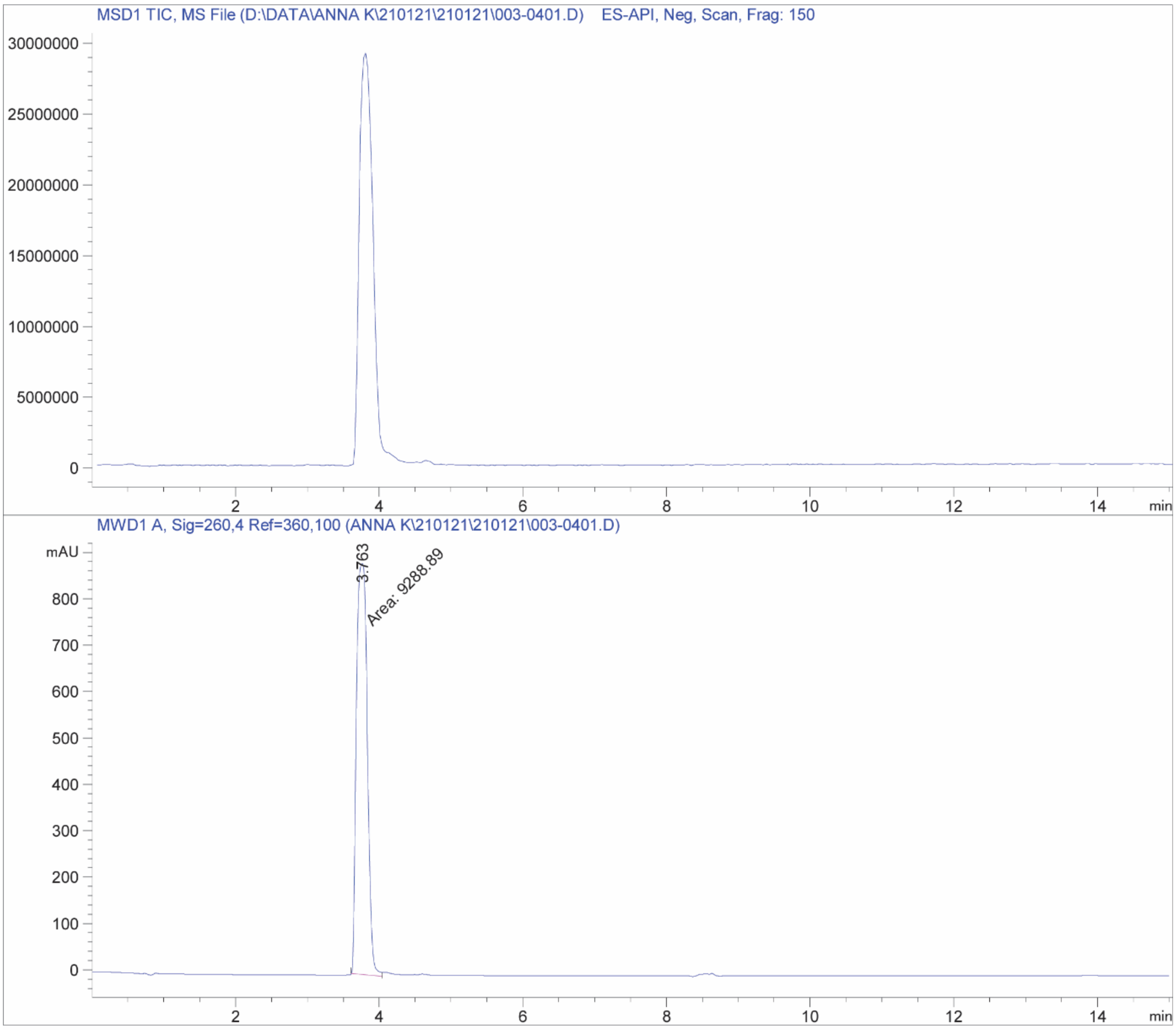

Signal 2: MWD1 A, Sig=260,4 Ref=360,100

Peak #	RetTime [min]	Type	Width [min]	Area [mAU*s]	Height [mAU]	Area %
1	3.763	PM	0.1748	9288.89355	885.65887	100.0000

Totals : 9288.89355 885.65887

Deconvolution of Spectrum # 1 @ 3.588 - 4.087 min

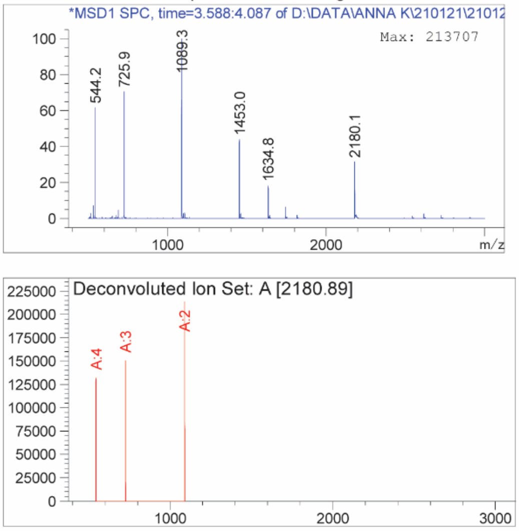

Component	Molecular Weight	Absolute Abundance	Relative Abundance
A	2180.89	490699	100.00

*** End of Report ***

*U*G*C*A*U*G*U

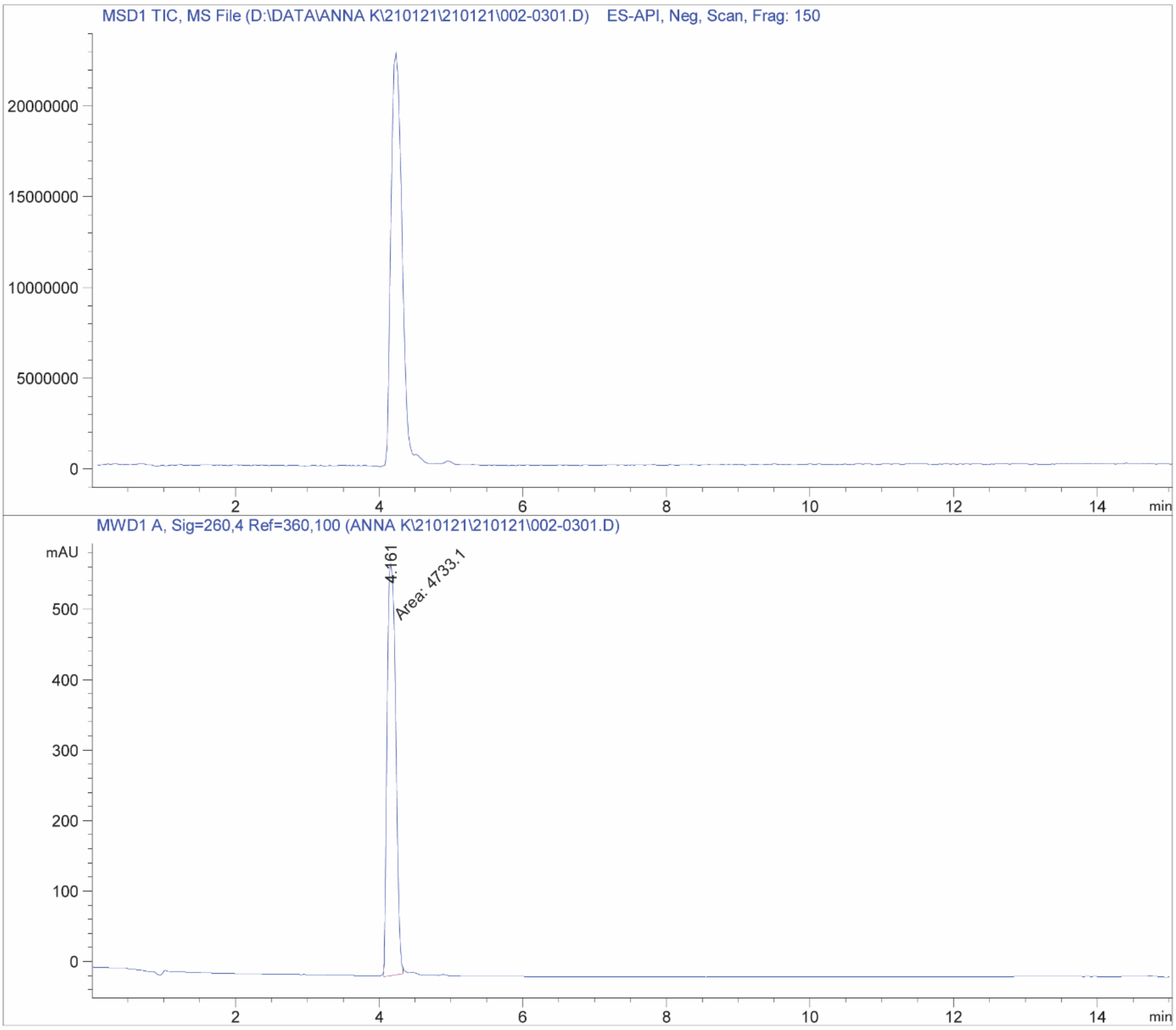

Signal 2: MWD1 A, Sig=260,4 Ref=360,100

Peak #	RetTime [min]	Type	Width [min]	Area [mAU*s]	Height [mAU]	Area %
1	4.161	MM	0.1350	4733.09570	584.51263	100.0000

Totals : 4733.09570 584.51263

Deconvolution of Spectrum # 1 @ 4.161 - 4.386 min

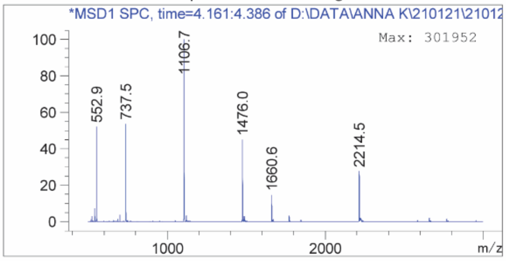

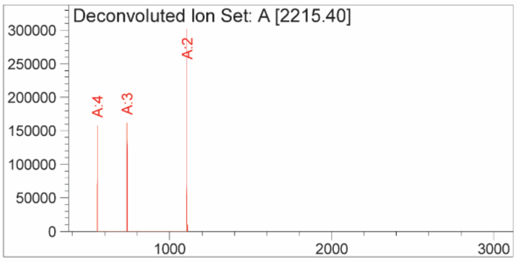

Component	Molecular Weight	Absolute Abundance	Relative Abundance
A	2215.40	619548	100.00

*** End of Report ***

*UGCAUGU

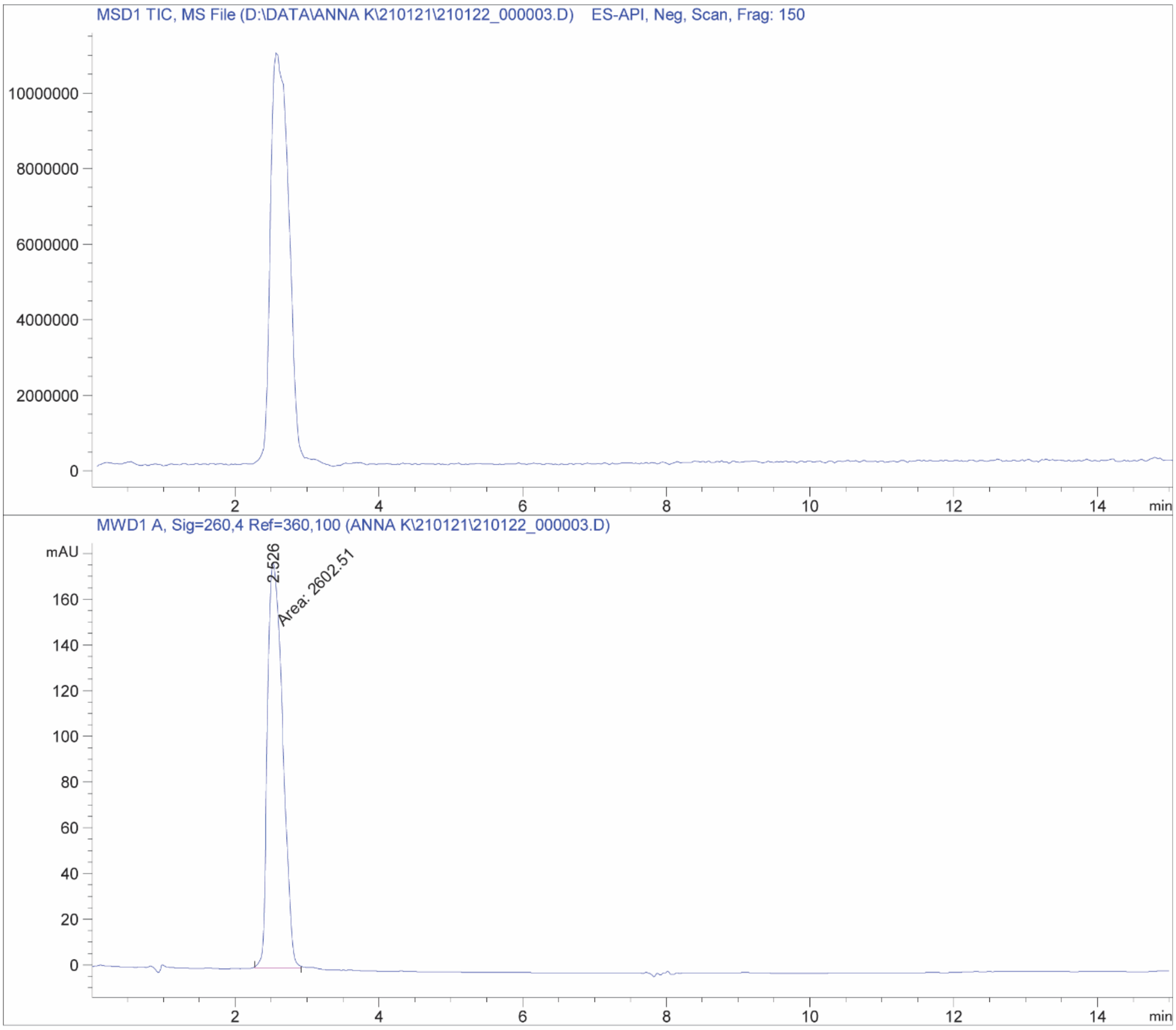

Signal 2: MWD1 A, Sig=260,4 Ref=360,100

Peak #	RetTime [min]	Type	Width [min]	Area [mAU*s]	Height [mAU]	Area %
1	2.526	MM	0.2451	2602.51318	176.95284	100.0000

Totals : 2602.51318 176.95284

Deconvolution of Spectrum # 1 @ 2.393 - 2.891 min

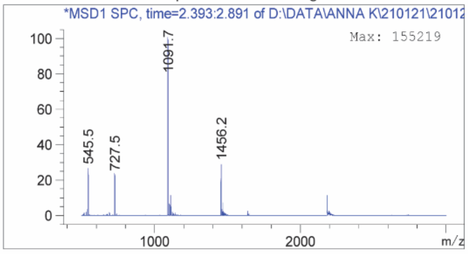

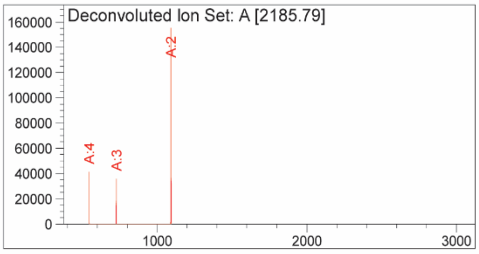

Component	Molecular Weight	Absolute Abundance	Relative Abundance
A	2185.79	229678	100.00
B	4371.52	196423	85.52

*** End of Report ***

U*GCAUGU

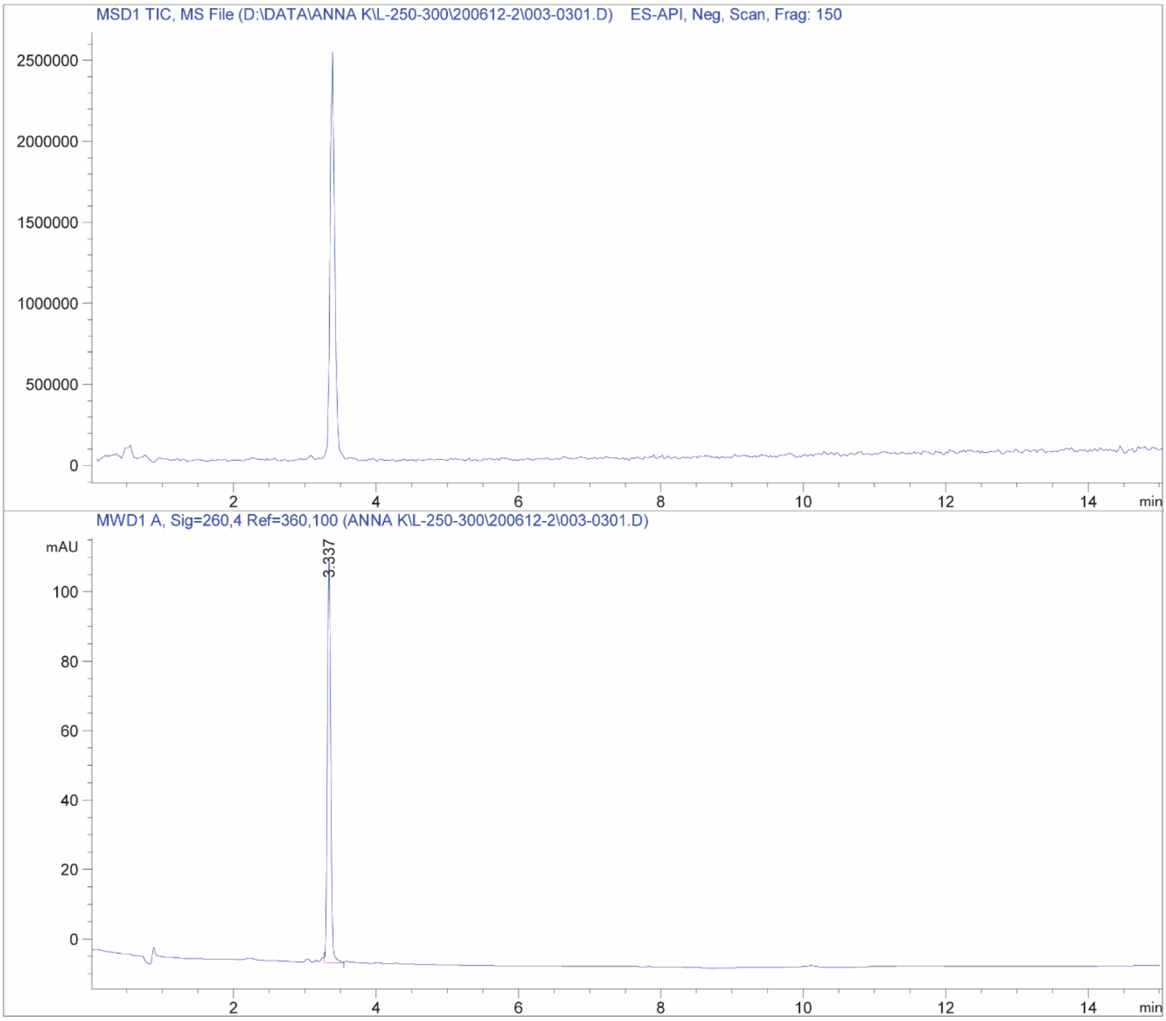

Signal 2: MWD1 A, Sig=260,4 Ref=360,100

Peak #	RetTime [min]	Type	Width [min]	Area [mAU*s]	Height [mAU]	Area %
1	3.337	VV	0.0495	376.93045	117.13254	100.0000
Totals :				376.93045	117.13254	

Deconvolution of Spectrum # 1 @ 3.065 - 3.563 min

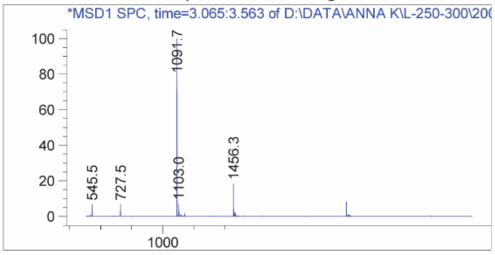

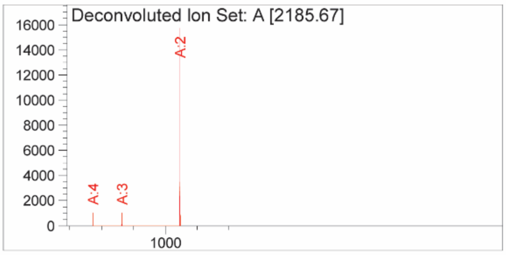

Component	Molecular Weight	Absolute Abundance	Relative Abundance
A	2185.67	17722	100.00

UG*CAUGU

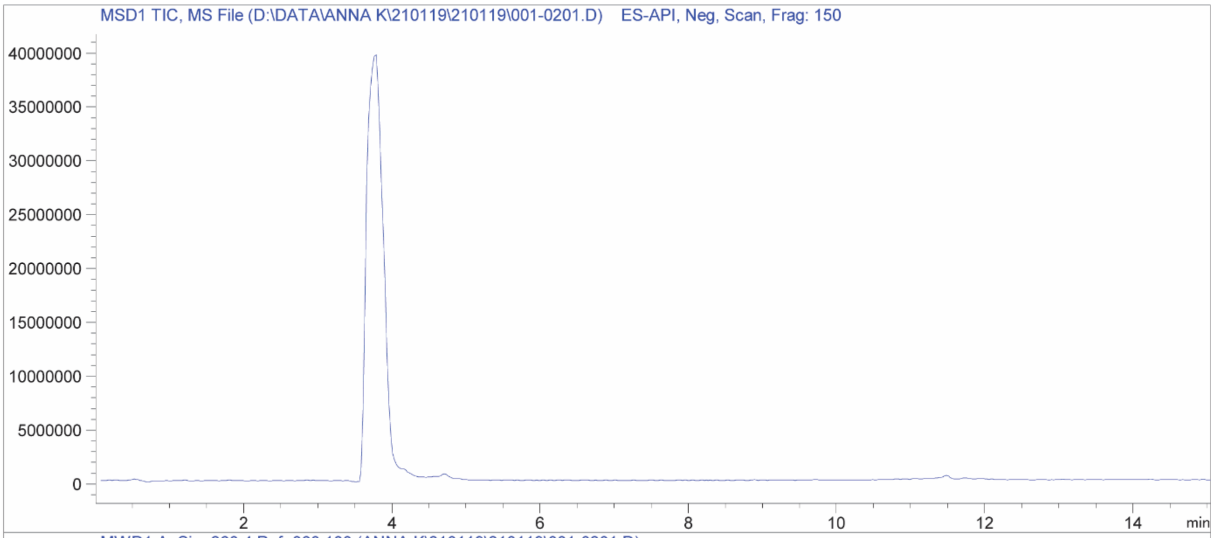

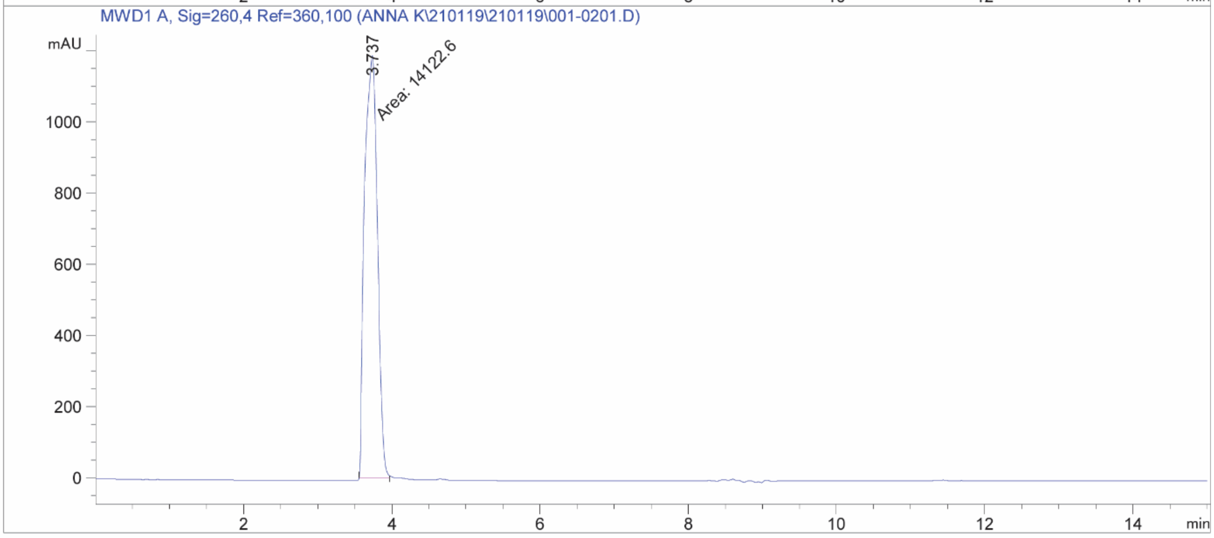

Signal 2: MWD1 A, Sig=260,4 Ref=360,100

Peak #	RetTime [min]	Type	Width [min]	Area [mAU*s]	Height [mAU]	Area %
1	3.737	PM	0.1982	1.41226e4	1187.32349	100.0000
Totals :				1.41226e4	1187.32349	

Deconvolution of Spectrum # 1 @ 3.439 - 4.012 min

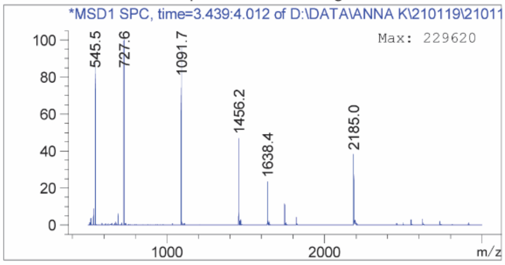

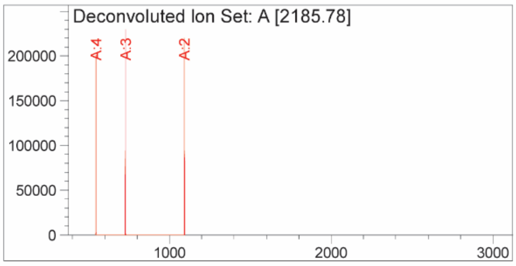

Component	Molecular Weight	Absolute Abundance	Relative Abundance
A	2185.78	641781	100.00

*** End of Report ***

UGC*AUGU

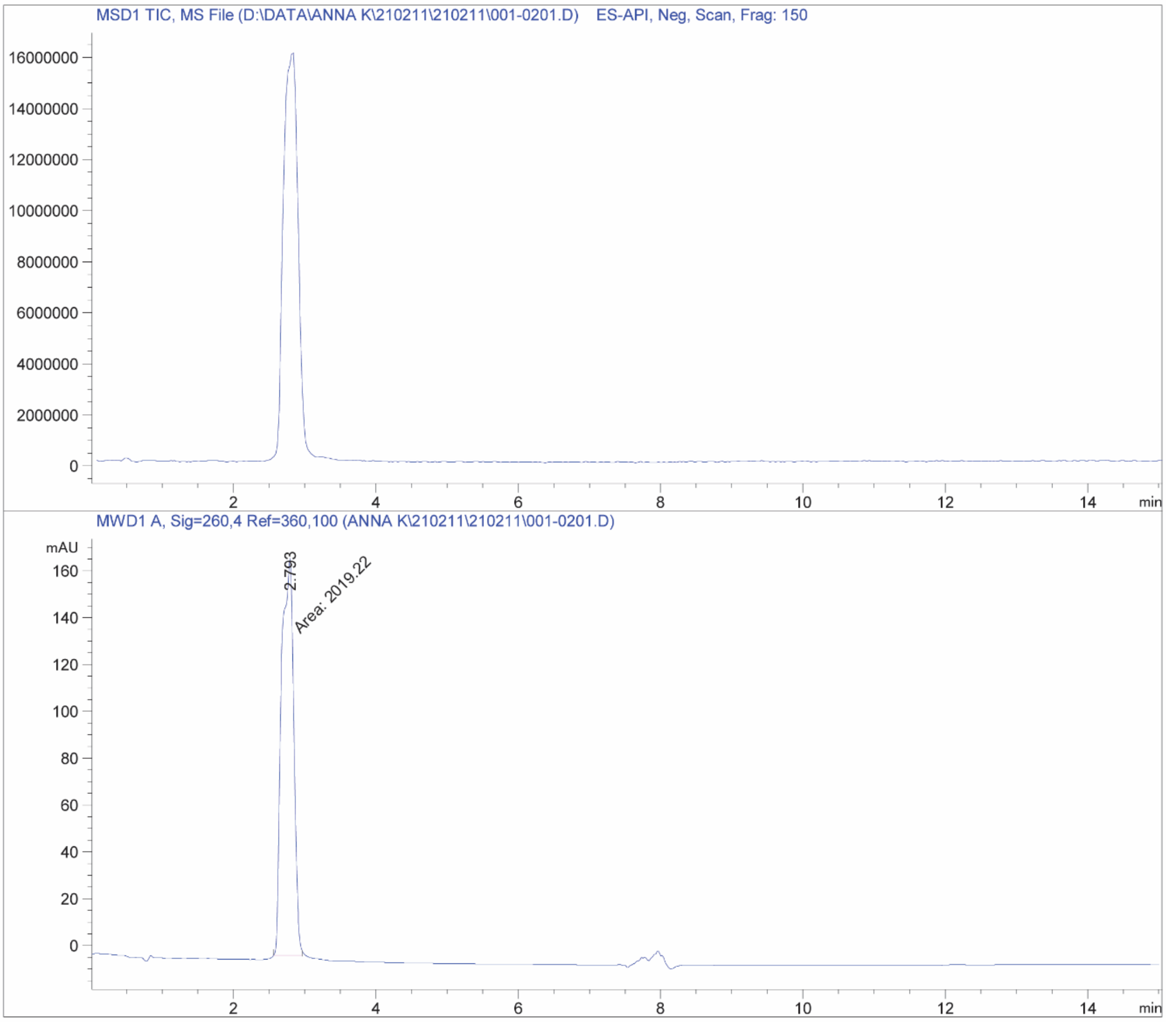


```
Signal 2: MWD1 A, Sig=260,4 Ref=360,100

Peak RetTime Type  Width     Area      Height     Area
  #   [min]        [min]   [mAU*s]     [mAU]       %
----|-------|----|-------|----------|----------|--------|
   1   2.793 PM    0.1989 2019.21643  169.21225 100.0000

Totals :                  2019.21643  169.21225
```

Deconvolution of Spectrum # 1 @ 2.442 - 3.115 min

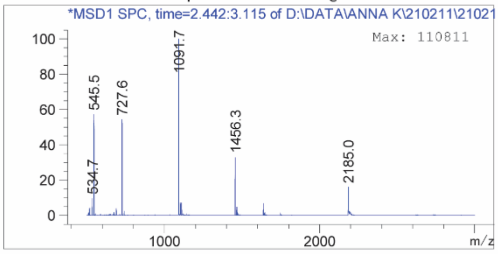

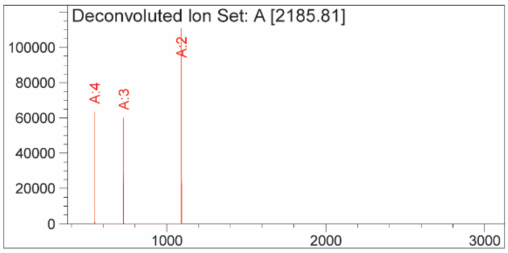

Component	Molecular Weight	Absolute Abundance	Relative Abundance
A	2185.81	232940	100.00

*** End of Report ***

UGCA*UGU

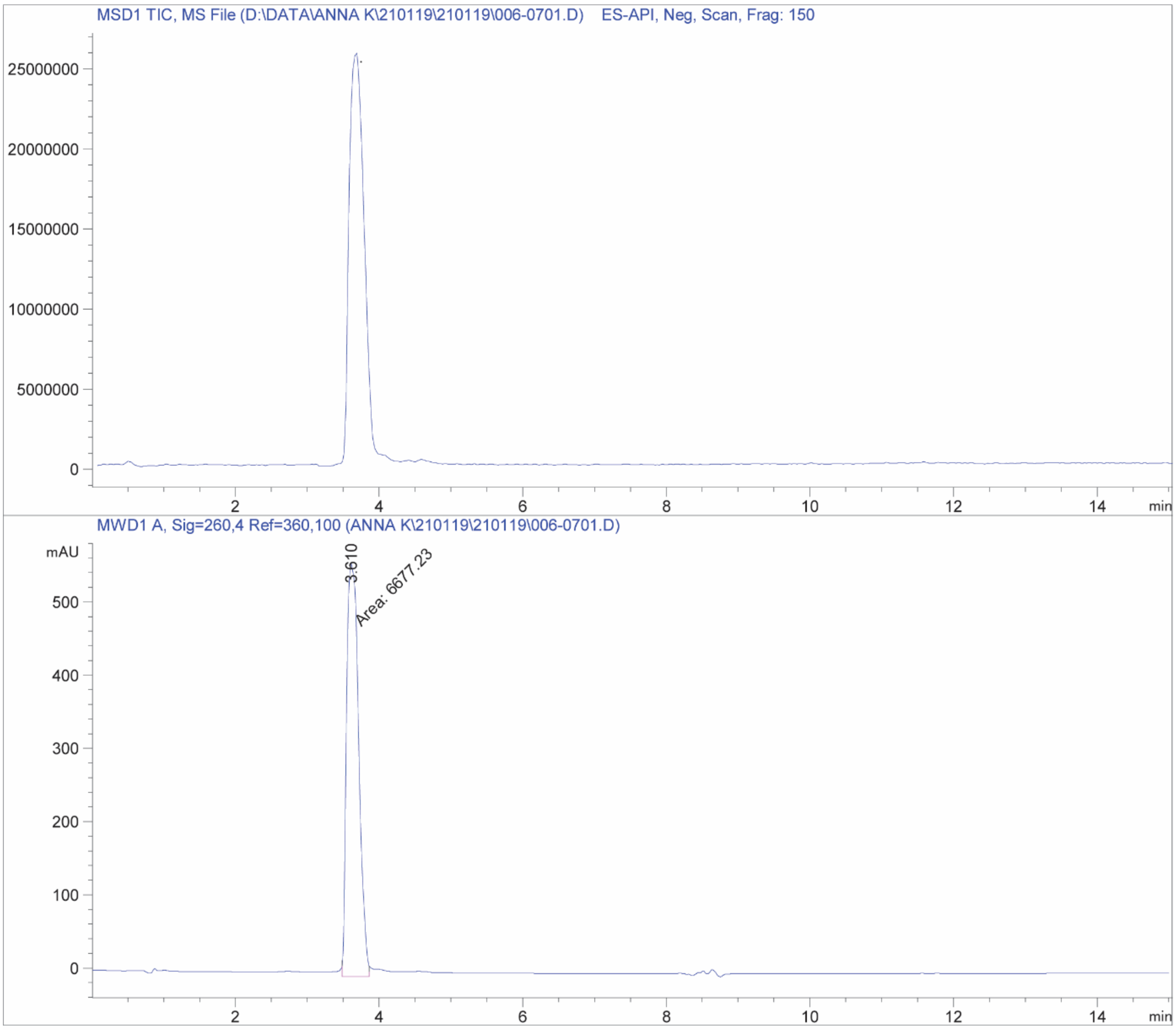

Signal 2: MWD1 A, Sig=260,4 Ref=360,100

Peak #	RetTime [min]	Type	Width [min]	Area [mAU*s]	Height [mAU]	Area %
1	3.610	MM	0.1974	6677.23438	563.68323	100.0000

Totals : 6677.23438 563.68323

Deconvolution of Spectrum # 1 @ 3.539 - 3.838 min

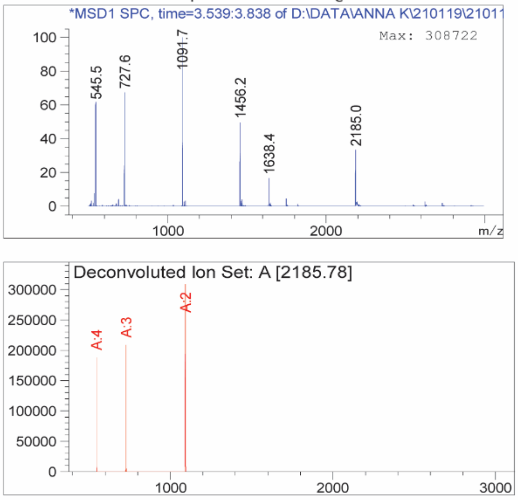

Component	Molecular Weight	Absolute Abundance	Relative Abundance
A	2185.78	695904	100.00

*** End of Report ***

UGCAU*GU

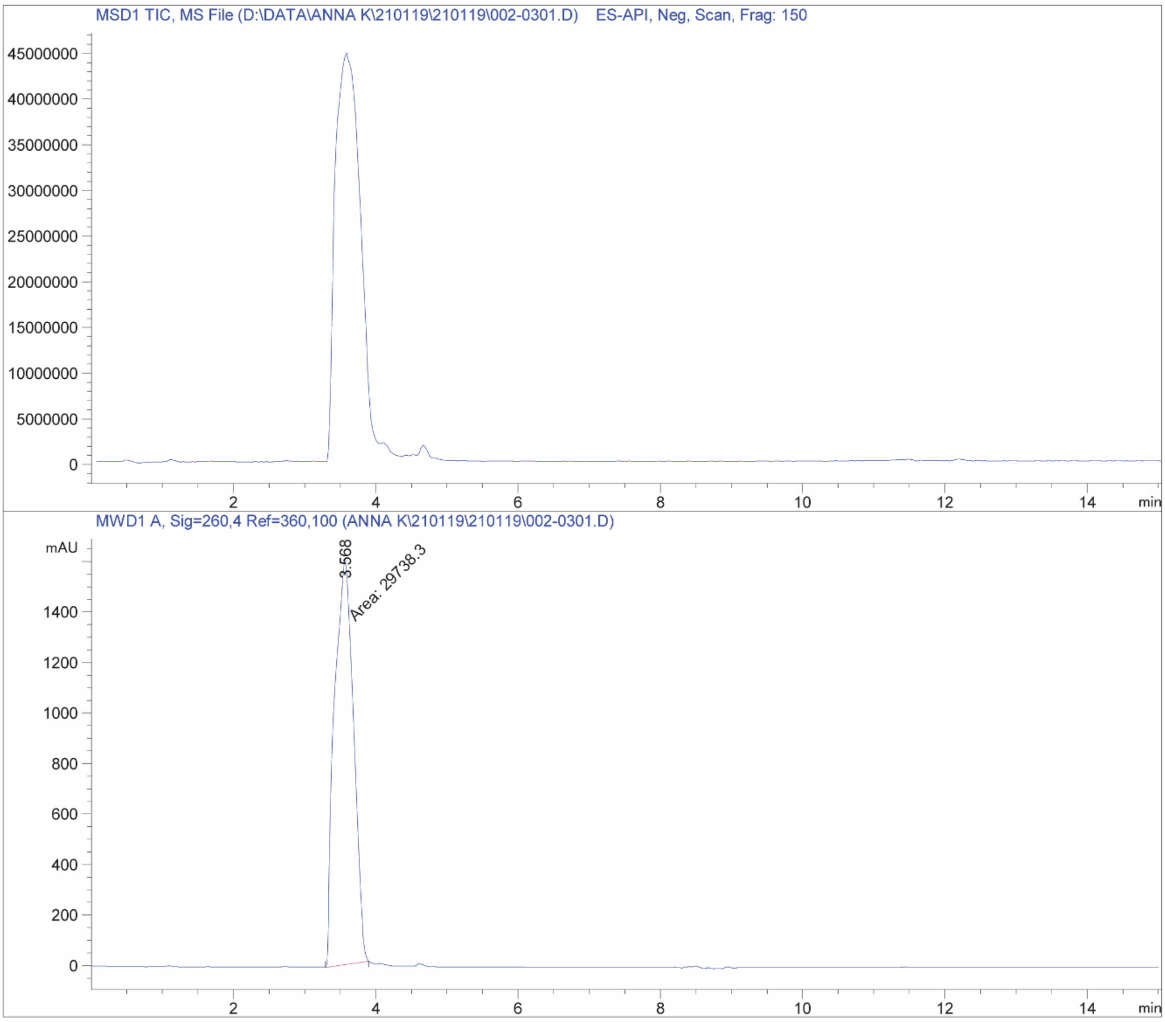


```
Signal 2: MWD1 A, Sig=260,4 Ref=360,100

Peak RetTime Type  Width     Area      Height      Area
  #   [min]        [min]   [mAU*s]     [mAU]        %
----|-------|----|-------|----------|----------|--------|
   1   3.568 MM    0.3086 2.97383e4  1605.95520 100.0000

Totals :                  2.97383e4  1605.95520
```

Deconvolution of Spectrum # 1 @ 3.115 - 4.087 min

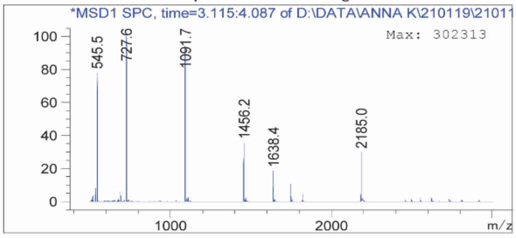

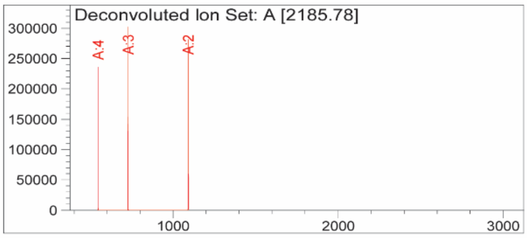


```
Component   Molecular      Absolute    Relative
             Weight        Abundance   Abundance
    A        2185.78        810249      100.00
                          *** End of Report ***
```

UGCAUG*U

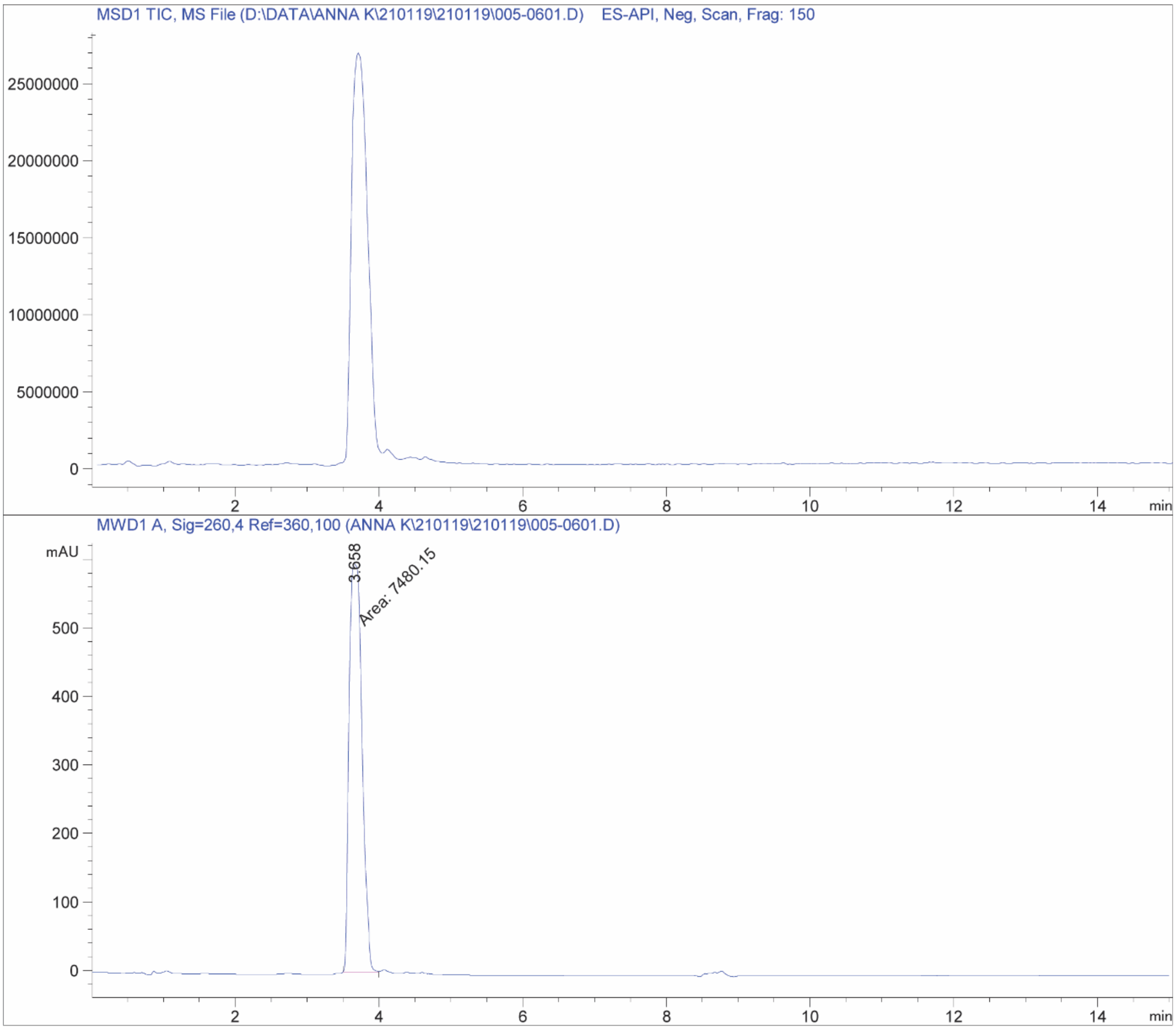


```
Signal 2: MWD1 A, Sig=260,4 Ref=360,100

Peak RetTime Type  Width     Area      Height     Area
  #   [min]        [min]   [mAU*s]     [mAU]       %
----|-------|----|-------|----------|----------|--------|
   1   3.658 MM     0.2090 7480.14600  596.61926 100.0000

Totals :                   7480.14600  596.61926
```

Deconvolution of Spectrum #   1 @ 3.638 - 3.862 min

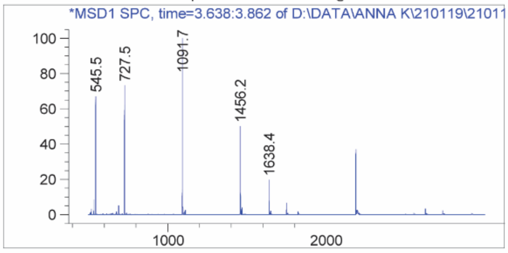

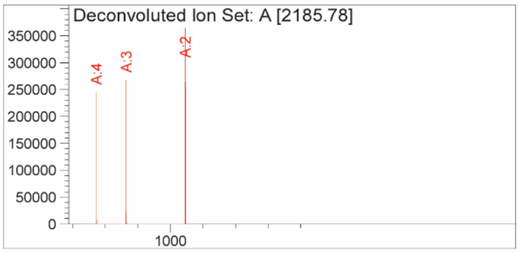


```
Component  Molecular      Absolute    Relative
             Weight       Abundance   Abundance
    A        2185.78       867296      100.00
                          *** End of Report ***
```

AGCAUGU

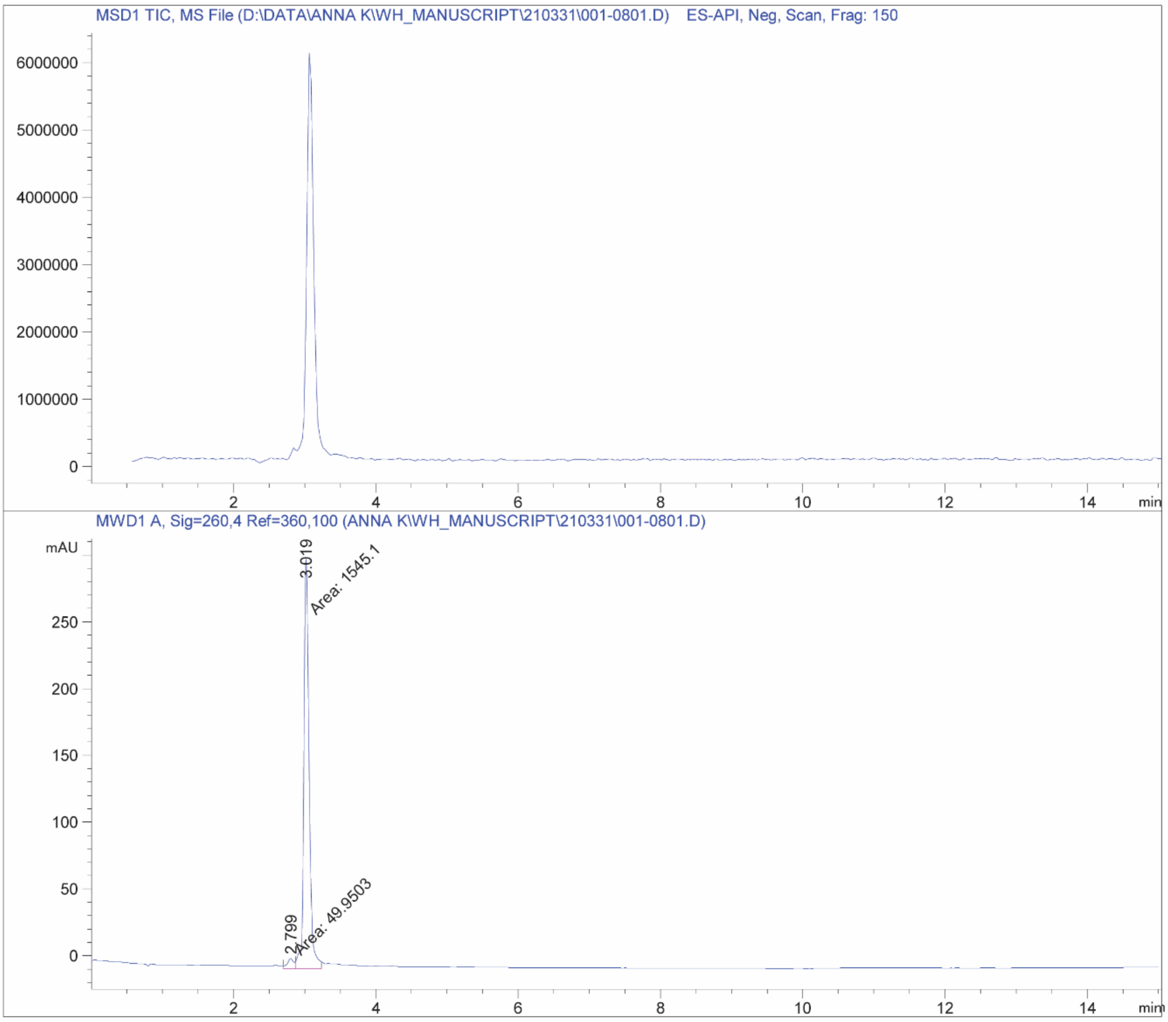

Signal 2: MWD1 A, Sig=260,4 Ref=360,100

Peak #	RetTime [min]	Type	Width [min]	Area [mAU*s]	Height [mAU]	Area %
1	2.799	MM	0.1086	49.95030	7.66722	3.1316
2	3.019	MM	0.0836	1545.09583	308.19852	96.8684

Totals : 1595.04612 315.86573

Deconvolution of Spectrum # 1 @ 2.891 - 3.264 min

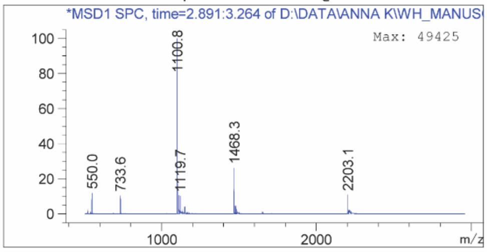

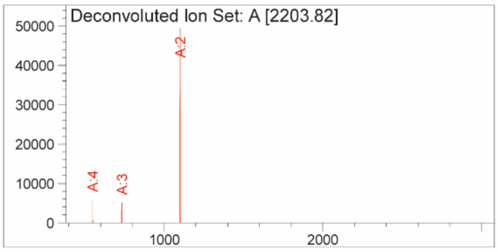

Component	Molecular Weight	Absolute Abundance	Relative Abundance
A	2203.82	60348	100.00

*** End of Report ***

*A*G*C*A*U*G*U

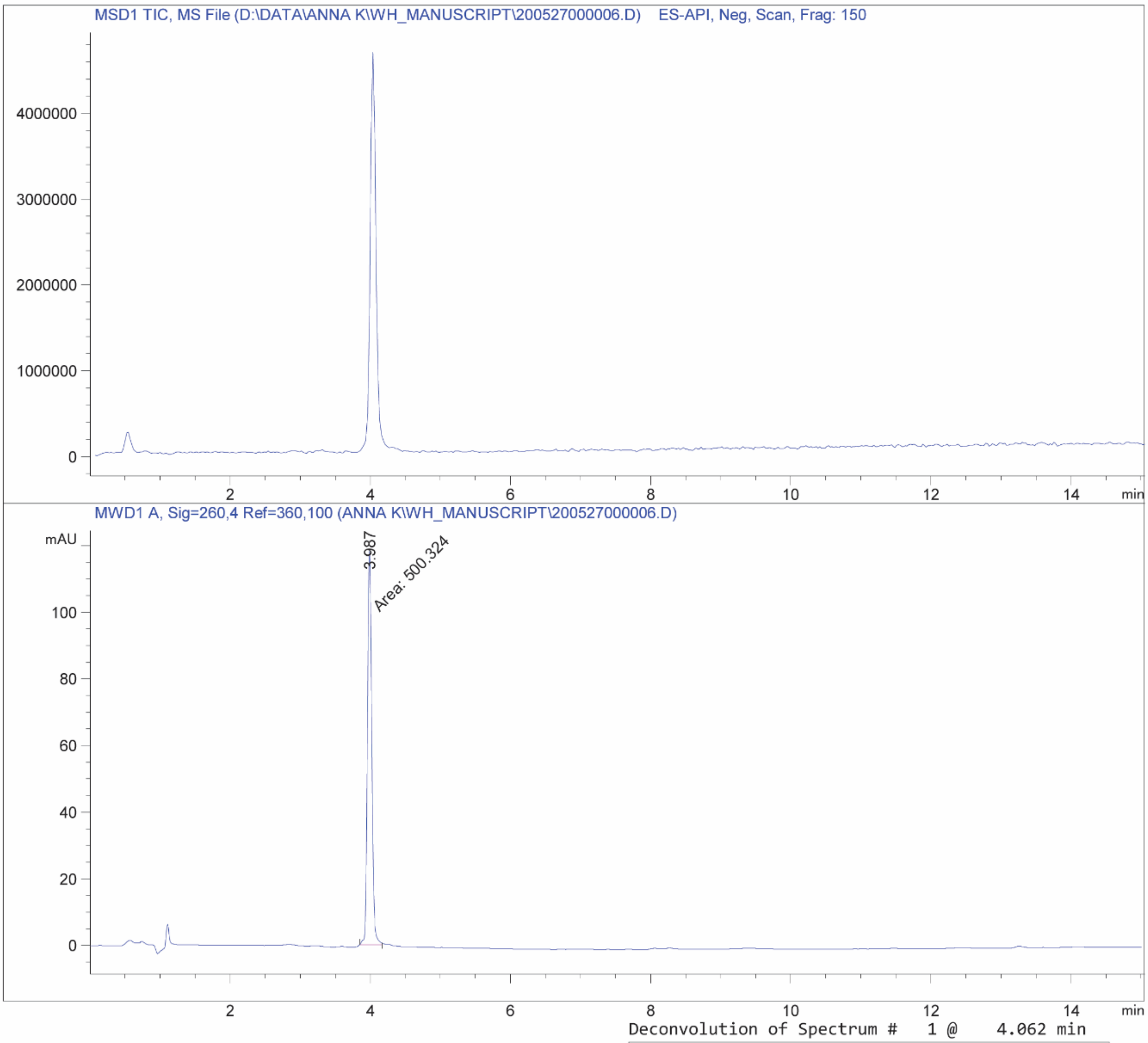

Deconvolution of Spectrum # 1 @ 4.062 min

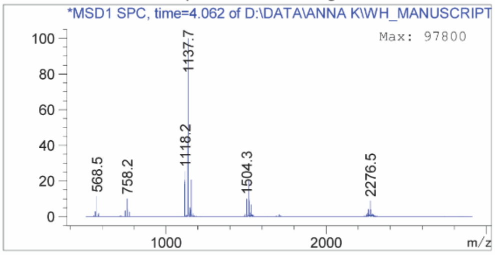

Signal 2: MWD1 A, Sig=260,4 Ref=360,100

Peak #	RetTime [min]	Type	Width [min]	Area [mAU*s]	Height [mAU]	Area %
1	3.987	PM	0.0703	500.32376	118.64858	100.0000
Totals :				500.32376	118.64858	

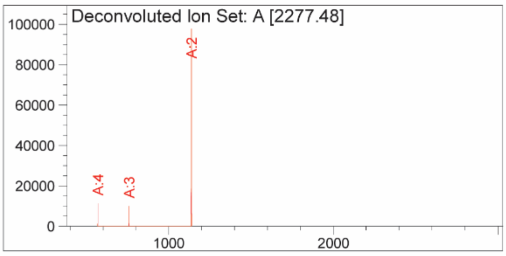

Component	Molecular Weight	Absolute Abundance	Relative Abundance
A	2277.48	118295	100.00

*AGCAUGU

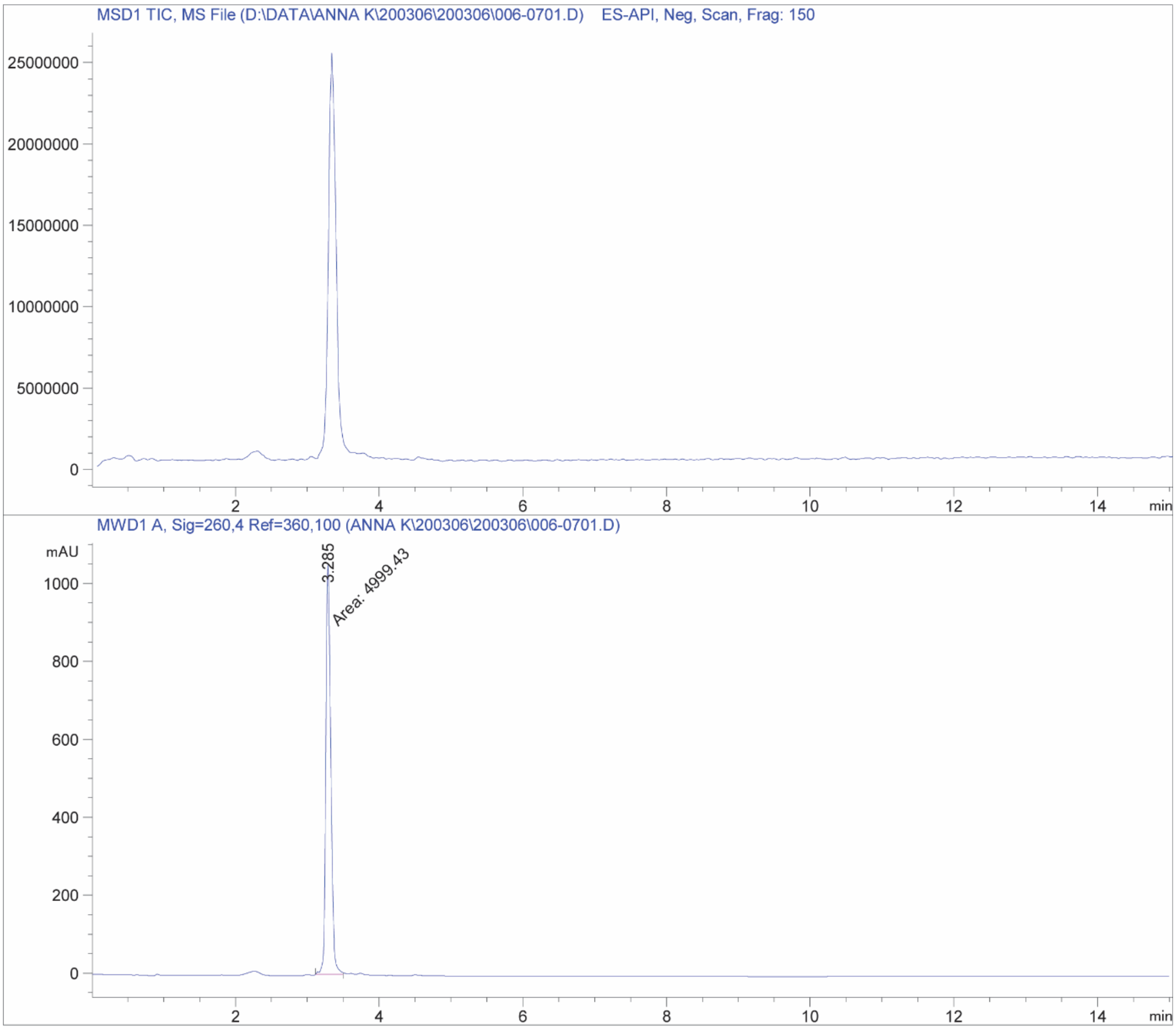

Signal 2: MWD1 A, Sig=260,4 Ref=360,100

Peak #	RetTime [min]	Type	Width [min]	Area [mAU*s]	Height [mAU]	Area %
1	3.285	PM	0.0788	4999.42725	1057.13831	100.0000

Totals : 4999.42725 1057.13831

Deconvolution of Spectrum # 1 @ 2.741 - 4.012 min

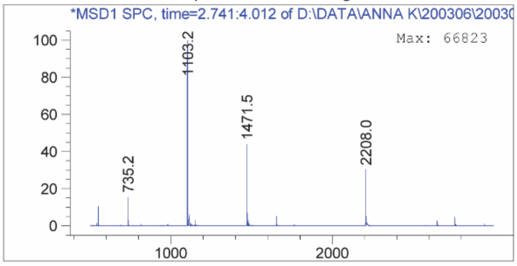

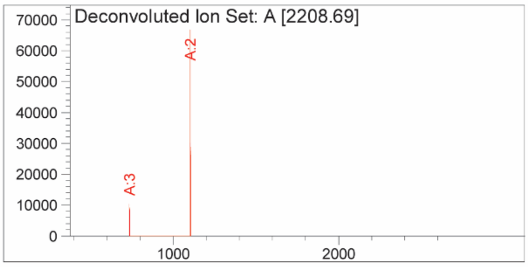

Component	Molecular Weight	Absolute Abundance	Relative Abundance
A	2208.69	76955	100.00

CGCAUGU

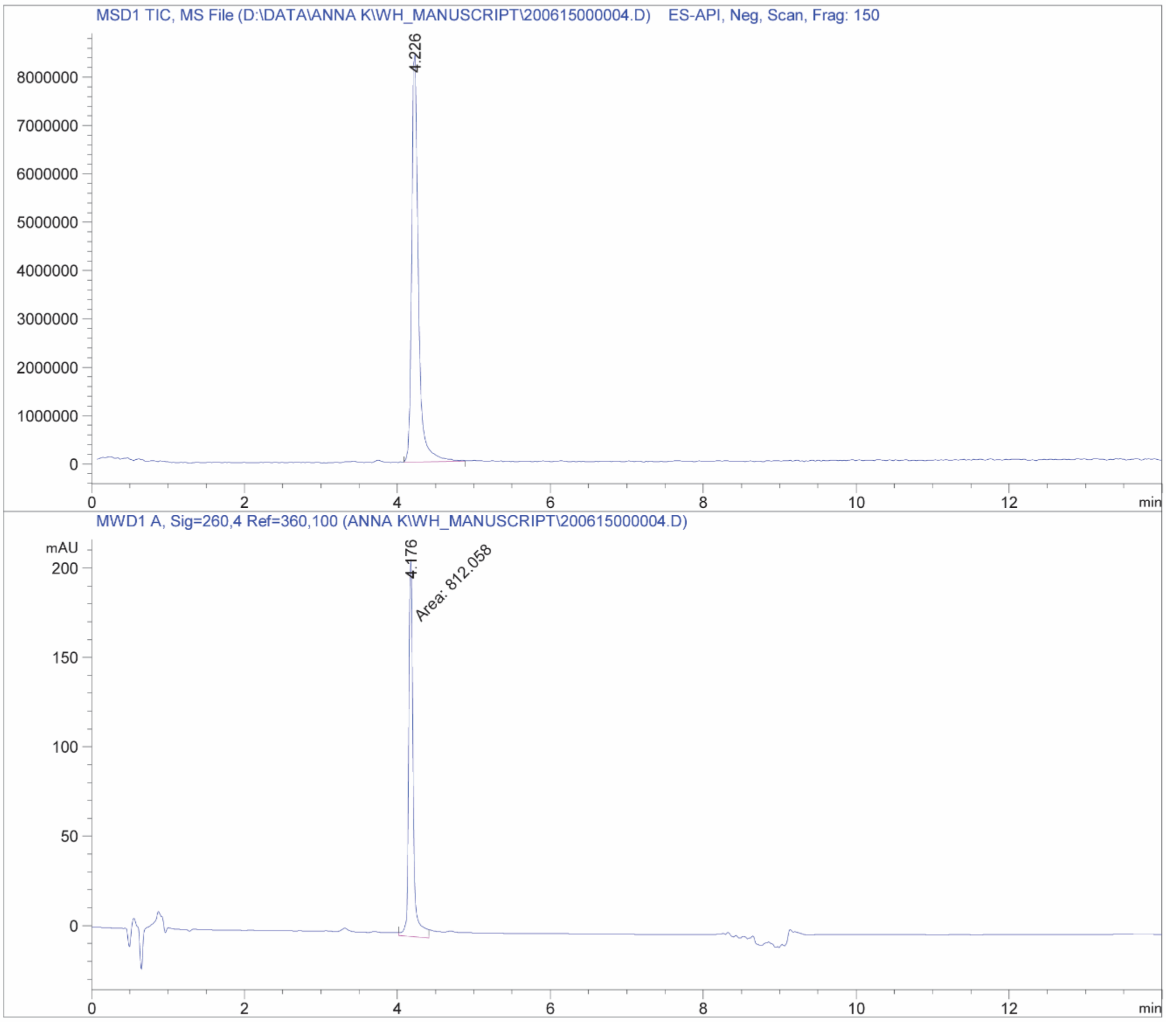

Signal 2: MWD1 A, Sig=260,4 Ref=360,100

Peak #	RetTime [min]	Type	Width [min]	Area [mAU*s]	Height [mAU]	Area %
1	4.176	MM	0.0638	812.05829	212.02115	100.0000
Totals :				812.05829	212.02115	

Deconvolution of Spectrum # 1 @ 4.136 - 4.361 min

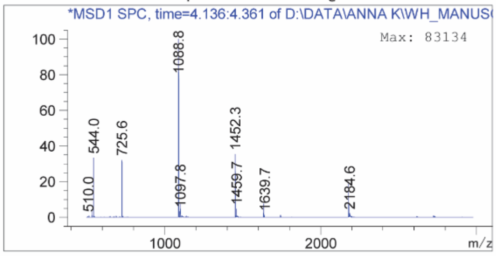

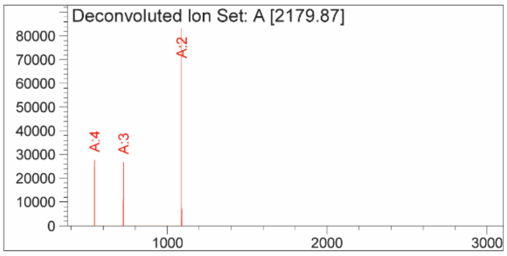

Component	Molecular Weight	Absolute Abundance	Relative Abundance
A	2179.87	137082	100.00

*C*G*C*A*U*G*U

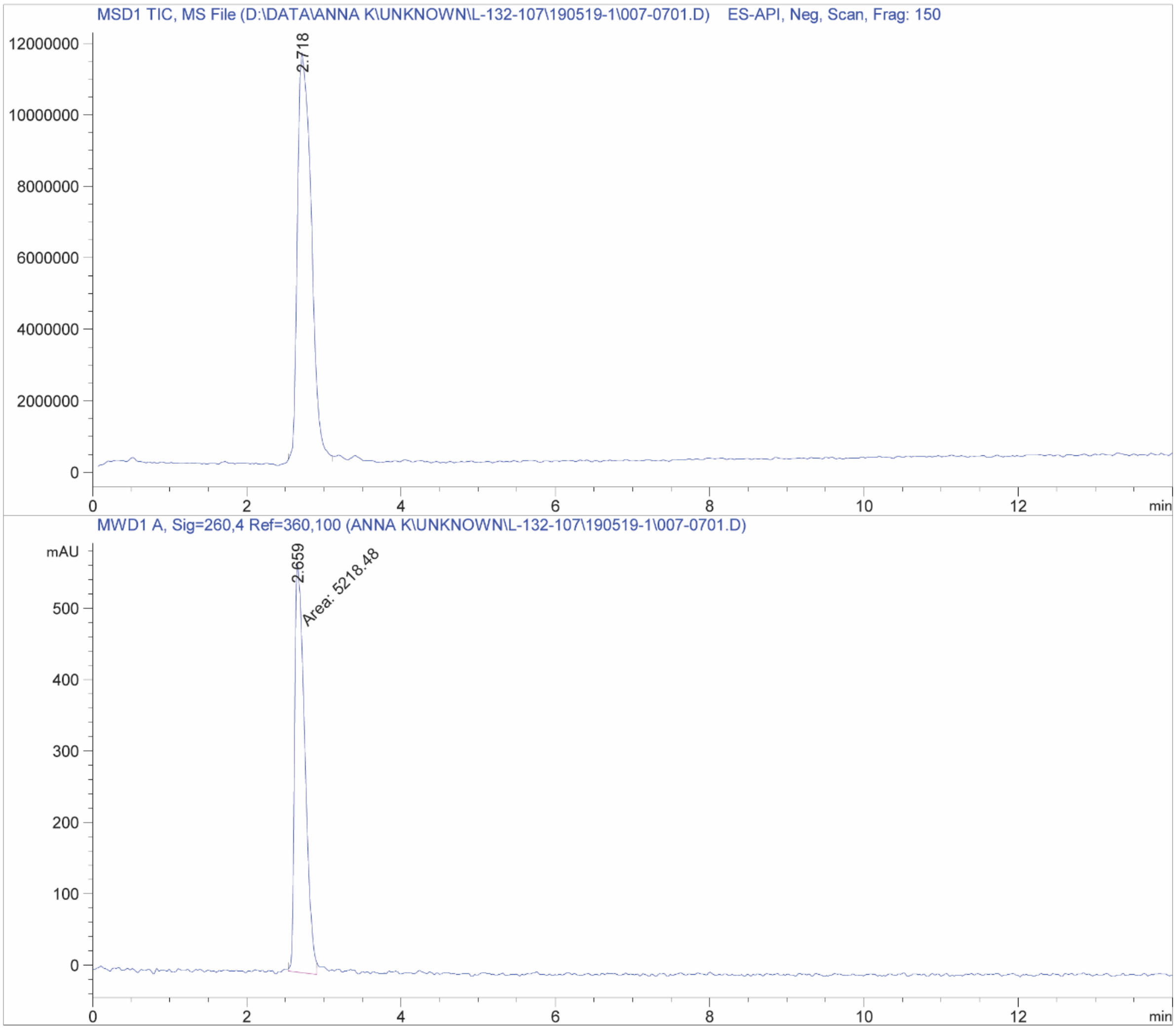

Signal 2: MWD1 A, Sig=260,4 Ref=360,100

Peak #	RetTime [min]	Type	Width [min]	Area [mAU*s]	Height [mAU]	Area %
1	2.659	MM	0.1519	5218.48047	572.42468	100.0000

Totals : 5218.48047 572.42468

Deconvolution of Spectrum # 1 @ 2.592 - 2.990 min

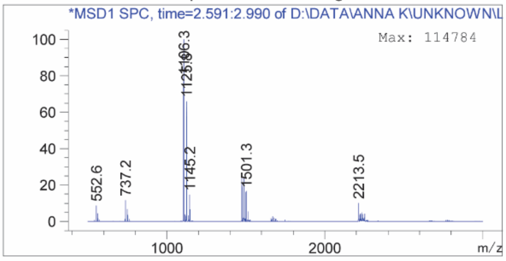

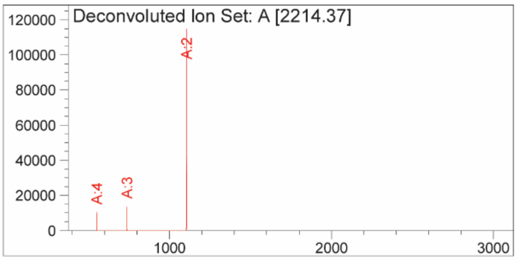

Component	Molecular Weight	Absolute Abundance	Relative Abundance
A	2214.37	137854	100.00

*CGCAUGU

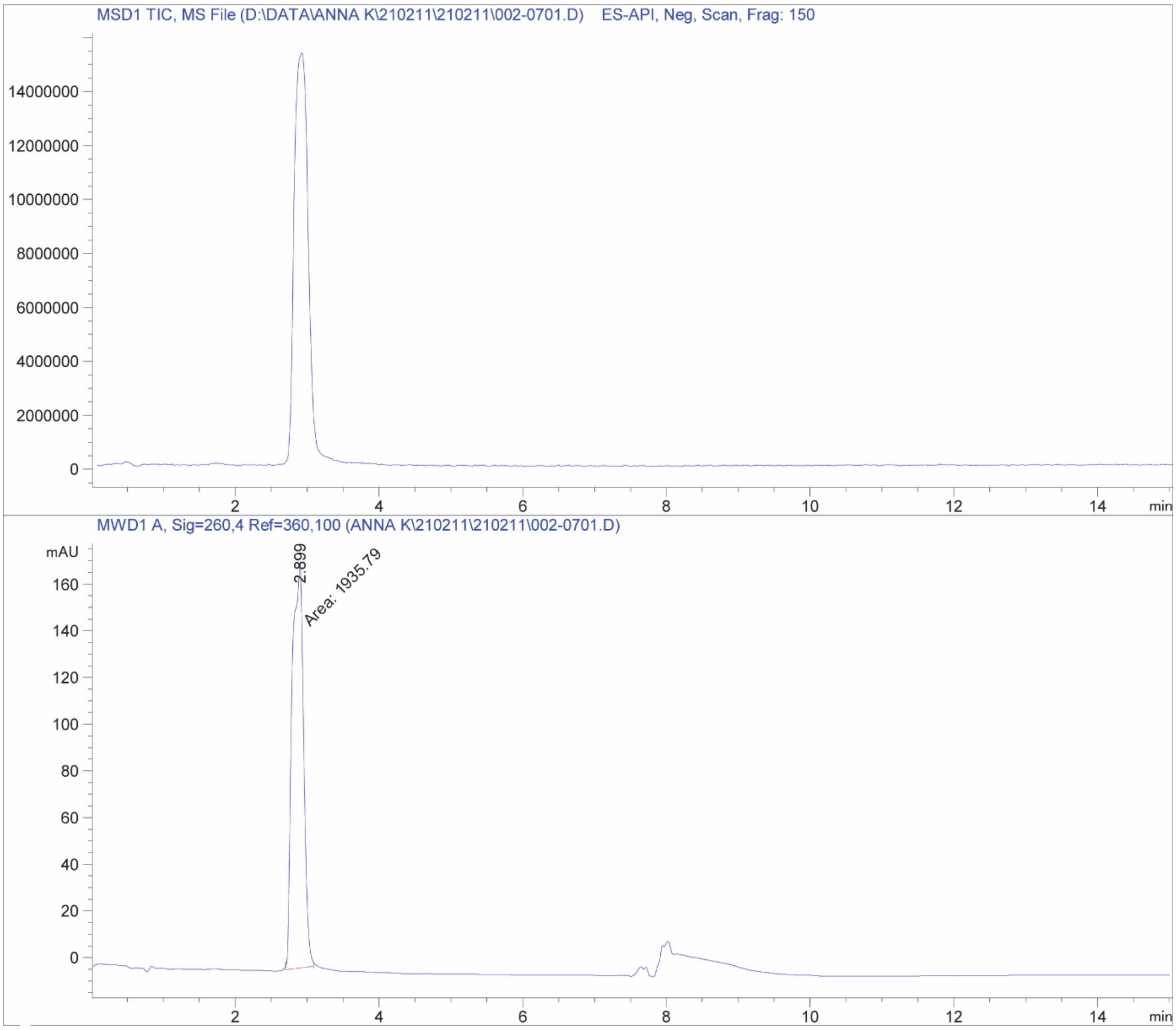

Signal 2: MWD1 A, Sig=260,4 Ref=360,100

Peak #	RetTime [min]	Type	Width [min]	Area [mAU*s]	Height [mAU]	Area %
1	2.899	MM	0.1862	1935.78821	173.25032	100.0000

Totals : 1935.78821 173.25032

Deconvolution of Spectrum # 1 @ 2.667 - 3.165 min

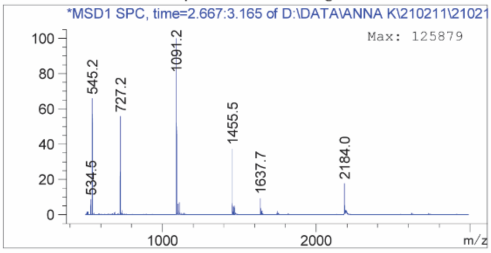

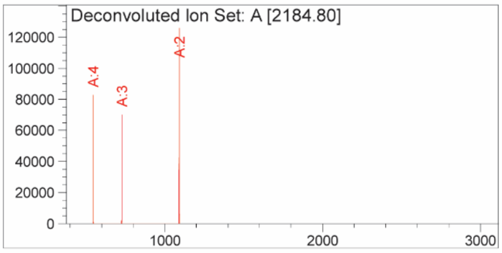

Component	Molecular Weight	Absolute Abundance	Relative Abundance
A	2184.80	275590	100.00

*** End of Report ***

GGCAUGU

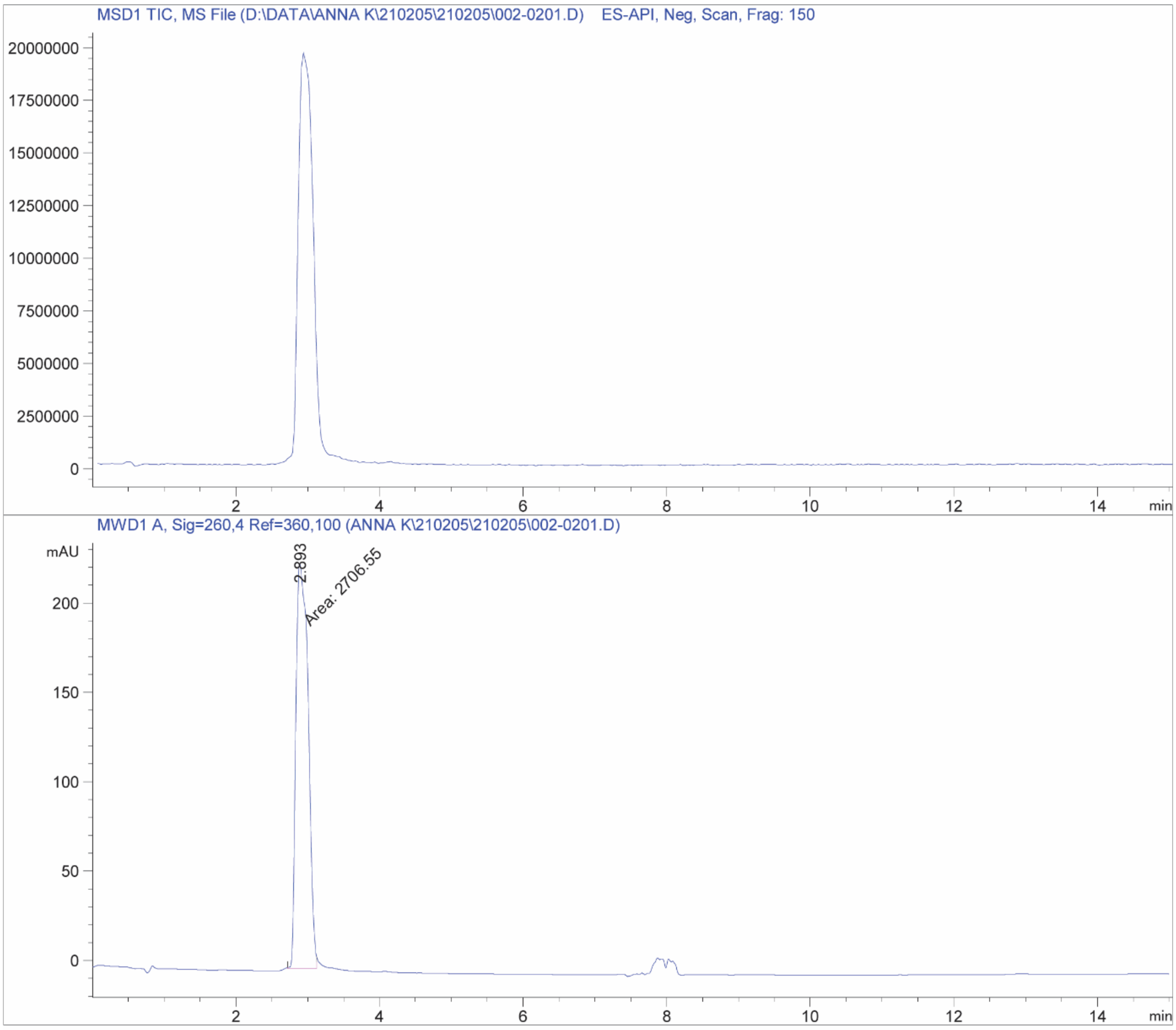

Signal 2: MWD1 A, Sig=260,4 Ref=360,100

Peak #	RetTime [min]	Type	Width [min]	Area [mAU*s]	Height [mAU]	Area %
1	2.893	MM	0.1990	2706.55396	226.66071	100.0000
Totals :				2706.55396	226.66071	

Deconvolution of Spectrum # 1 @ 2.591 - 3.264 min

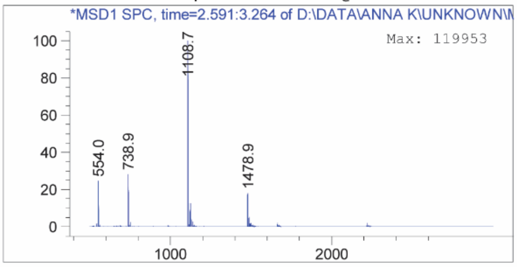

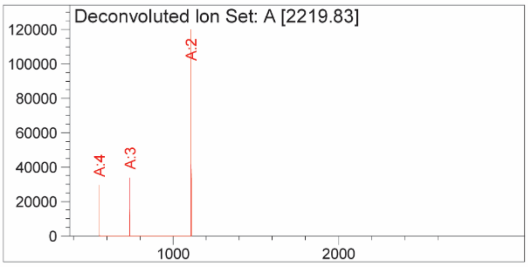

Component	Molecular Weight	Absolute Abundance	Relative Abundance
A	2219.83	179813	100.00

*G*G*C*A*U*G*U

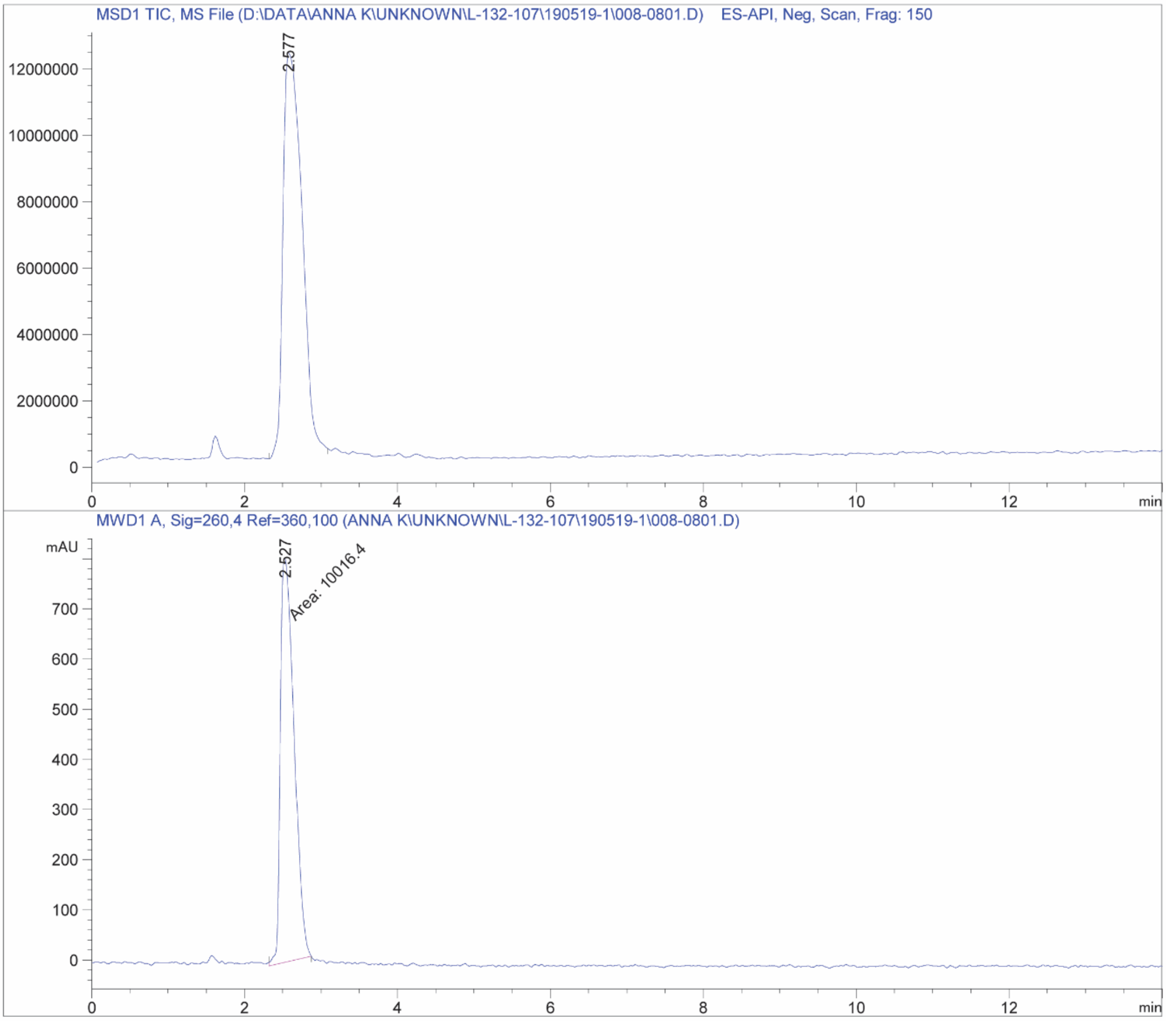

Signal 2: MWD1 A, Sig=260,4 Ref=360,100

Peak #	RetTime [min]	Type	Width [min]	Area [mAU*s]	Height [mAU]	Area %
1	2.527	MP	0.2073	1.00164e4	805.31378	100.0000
Totals :				1.00164e4	805.31378	

Deconvolution of Spectrum # 1 @ 2.442 - 2.940 min

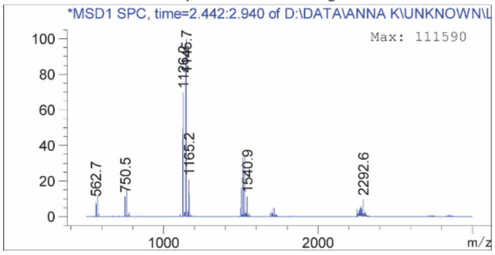

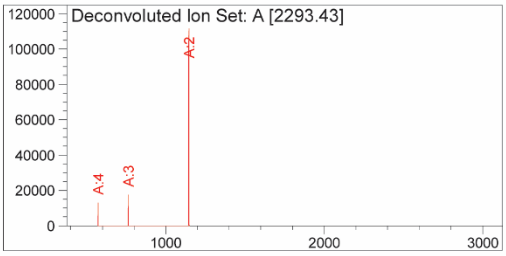

Component	Molecular Weight	Absolute Abundance	Relative Abundance
A	2293.43	142031	100.00
B	2254.38	99958	70.38

*GGCAUGU

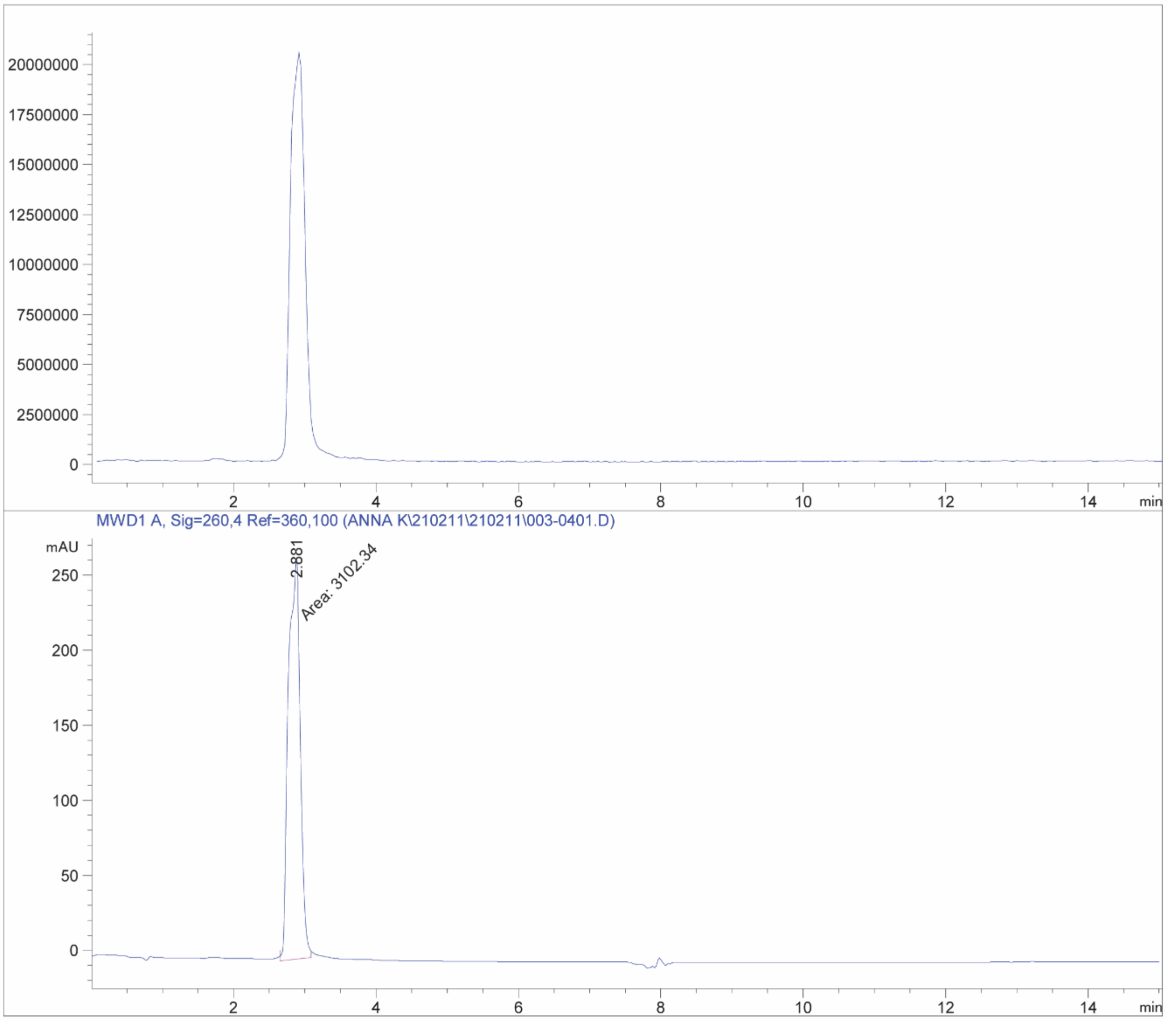

Signal 2: MWD1 A, Sig=260,4 Ref=360,100

Peak #	RetTime [min]	Type	Width [min]	Area [mAU*s]	Height [mAU]	Area %
1	2.881	MM	0.1936	3102.34375	267.03357	100.0000
Totals :				3102.34375	267.03357	

Deconvolution of Spectrum # 1 @ 2.667 - 3.165 min

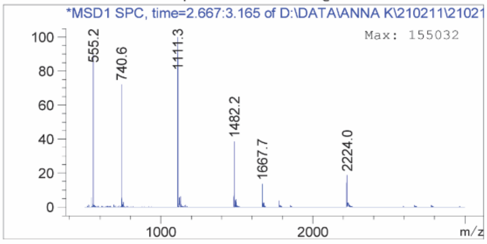

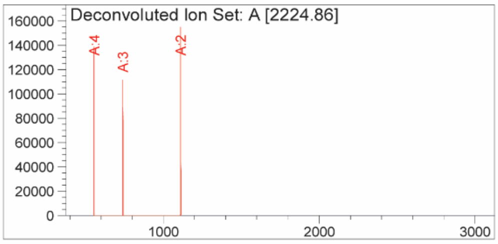

Component	Molecular Weight	Absolute Abundance	Relative Abundance
A	2224.86	401898	100.00

*** End of Report ***

UGGAUGU

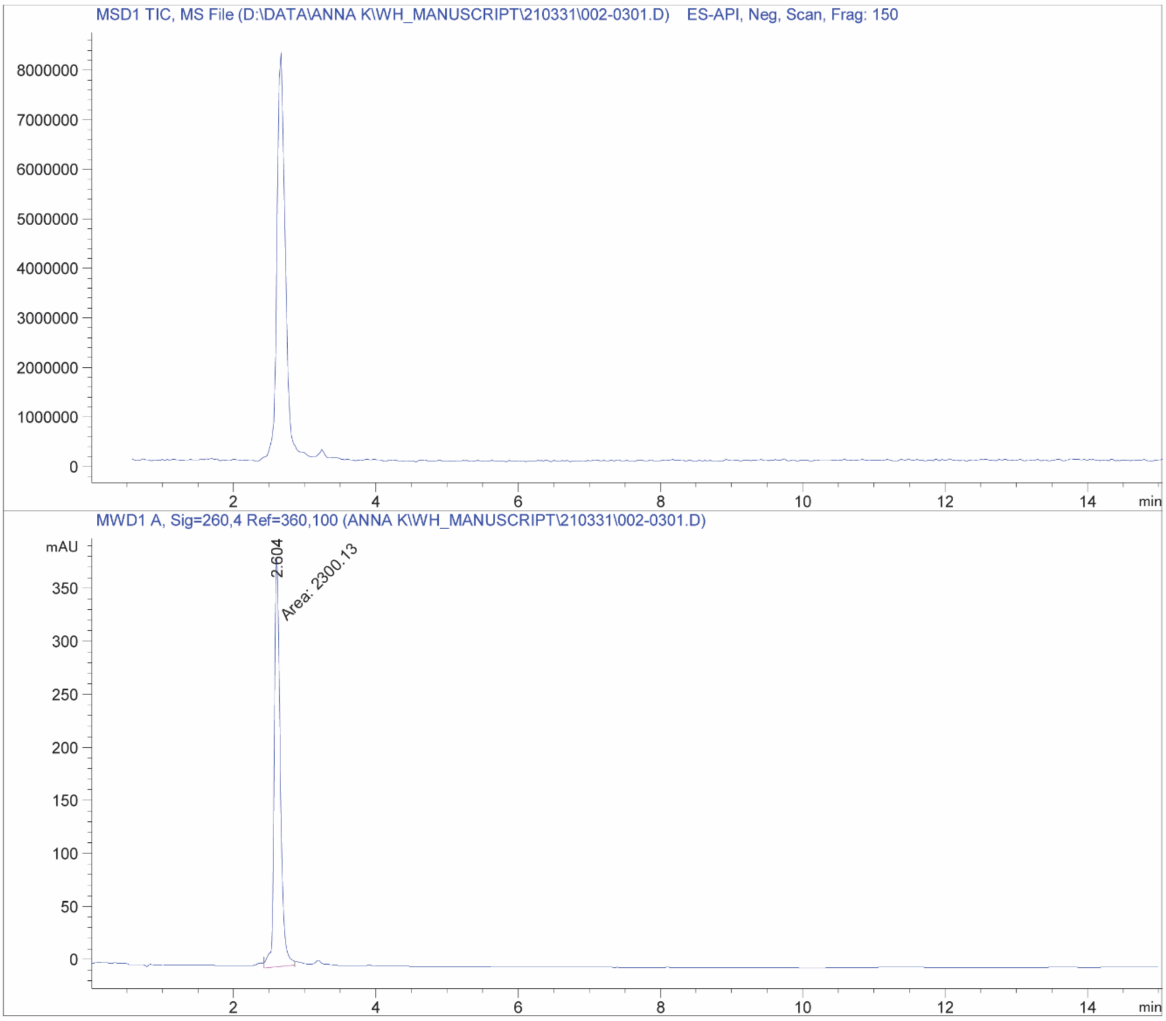

Deconvolution of Spectrum # 1 @ 2.492 - 2.866 min

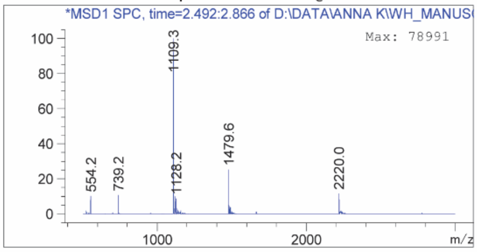

Signal 2: MWD1 A, Sig=260,4 Ref=360,100

Peak #	RetTime [min]	Type	Width [min]	Area [mAU*s]	Height [mAU]	Area %
1	2.604	MM	0.0994	2300.13428	385.80093	100.0000
Totals :				2300.13428	385.80093	

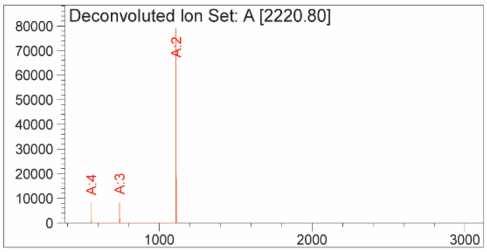

Component	Molecular Weight	Absolute Abundance	Relative Abundance
A	2220.80	95266	100.00

*** End of Report ***

*U*G*G*A*U*G*U

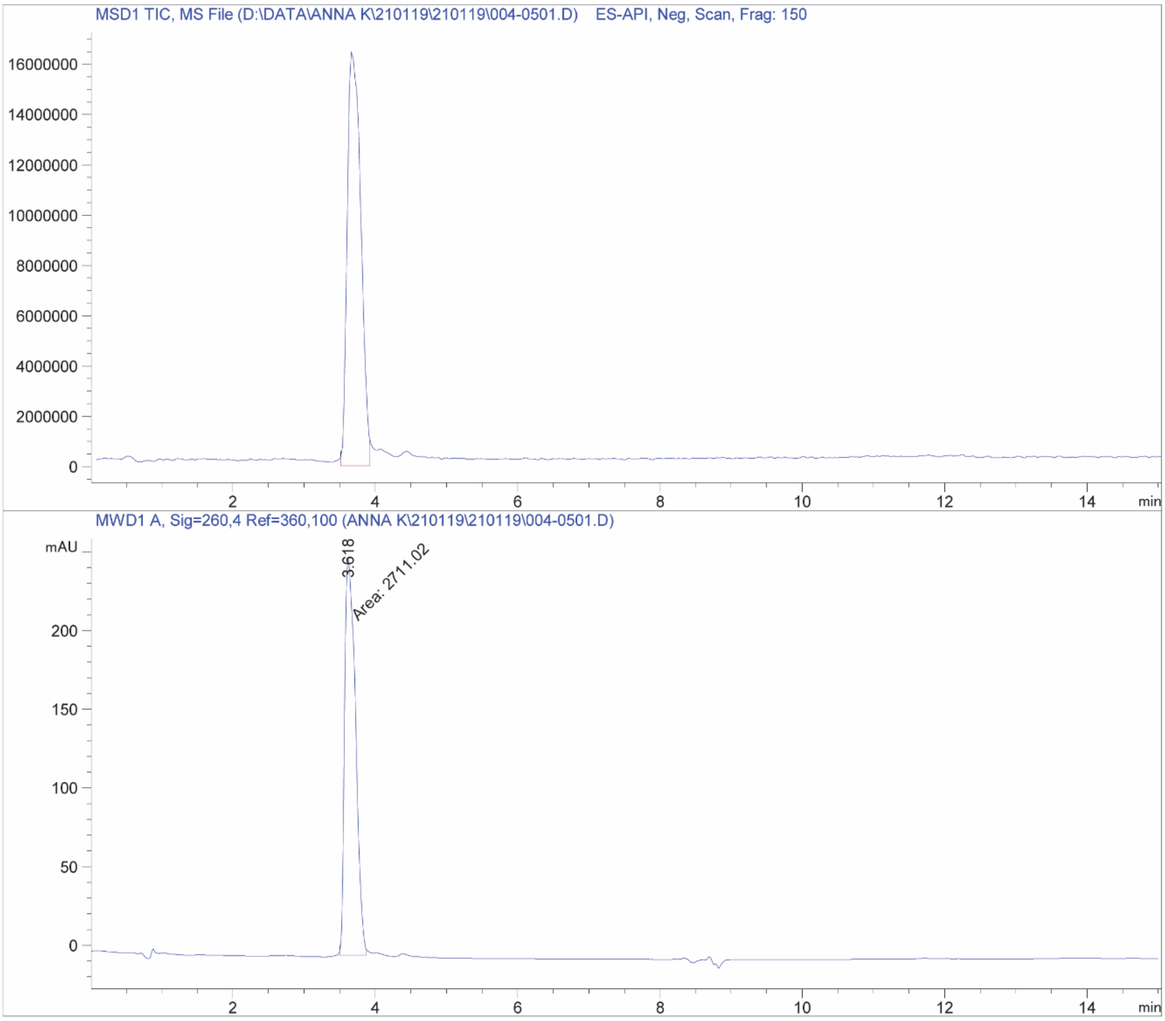

Signal 2: MWD1 A, Sig=260,4 Ref=360,100

Peak #	RetTime [min]	Type	Width [min]	Area [mAU*s]	Height [mAU]	Area %
1	3.618	MM	0.1793	2711.02197	252.06017	100.0000
Totals :				2711.02197	252.06017	

Deconvolution of Spectrum # 1 @ 3.514 - 3.912 min

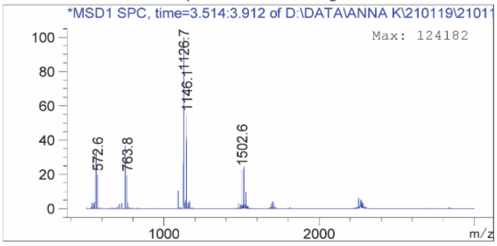

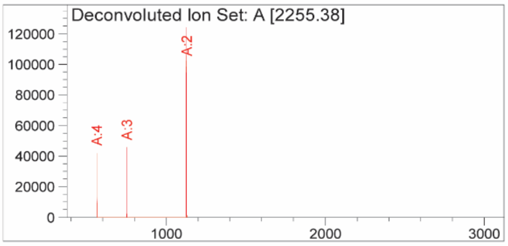

Component	Molecular Weight	Absolute Abundance	Relative Abundance
A	2255.38	211594	100.00
B	2294.35	116062	54.85

*** End of Report ***

UG*GAUGU

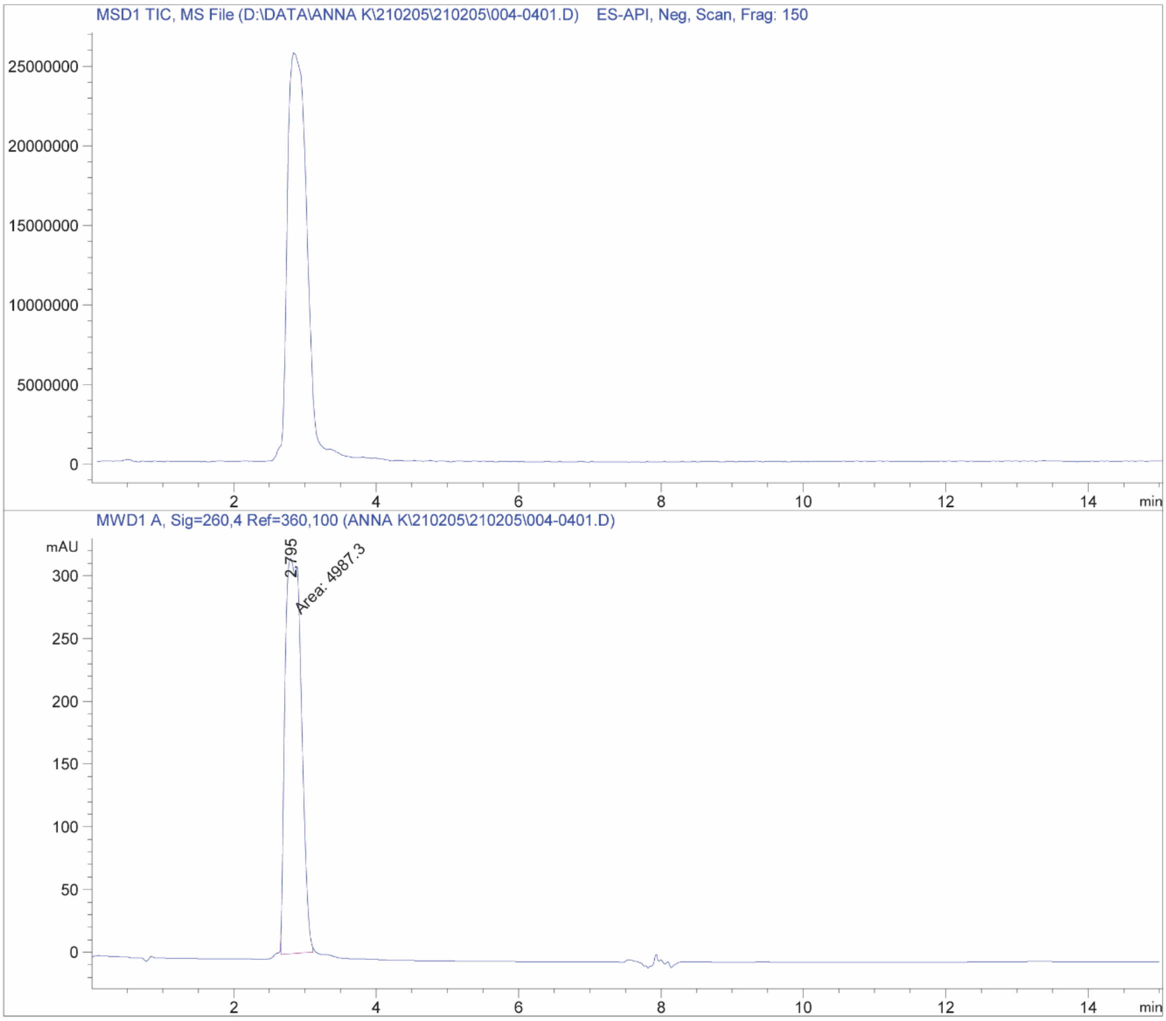

Signal 2: MWD1 A, Sig=260,4 Ref=360,100

Peak #	RetTime [min]	Type	Width [min]	Area [mAU*s]	Height [mAU]	Area %
1	2.795	MM	0.2640	4987.30273	314.87326	100.0000
Totals :				4987.30273	314.87326	

Deconvolution of Spectrum # 1 @ 2.467 - 3.364 min

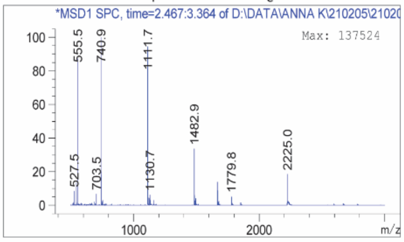

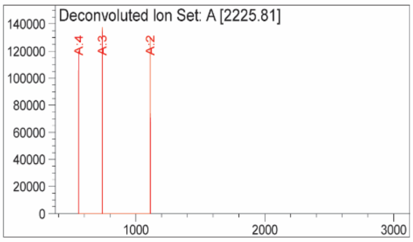

Component	Molecular Weight	Absolute Abundance	Relative Abundance
A	2225.81	382367	100.00

*** End of Report ***

UGCGUGU

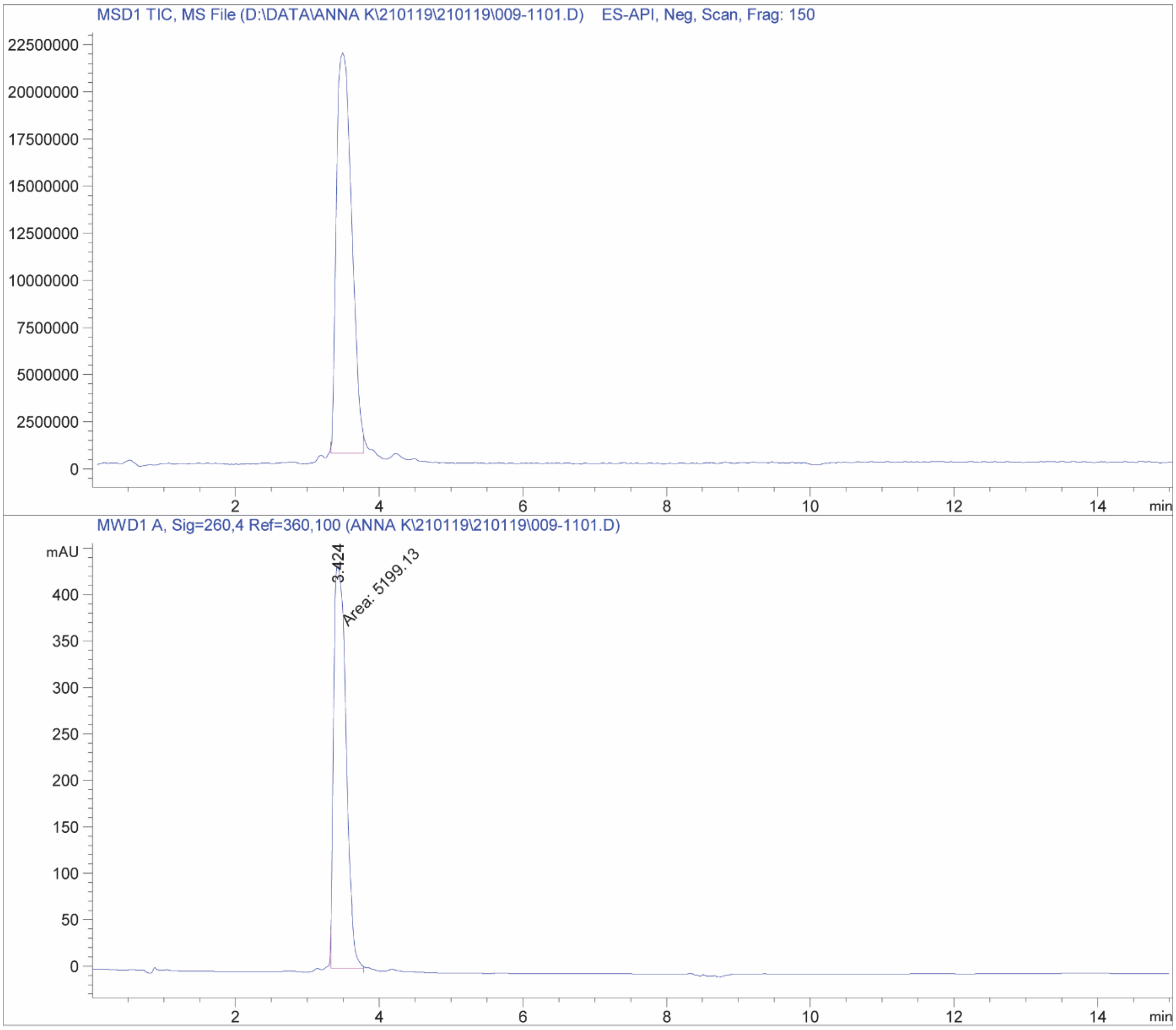

Signal 2: MWD1 A, Sig=260,4 Ref=360,100

Peak #	RetTime [min]	Type	Width [min]	Area [mAU*s]	Height [mAU]	Area %
1	3.424	MM	0.1991	5199.13135	435.29019	100.0000

Totals : 5199.13135 435.29019

Deconvolution of Spectrum # 1 @ 3.339 - 3.688 min

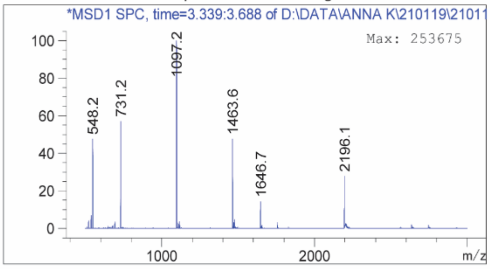

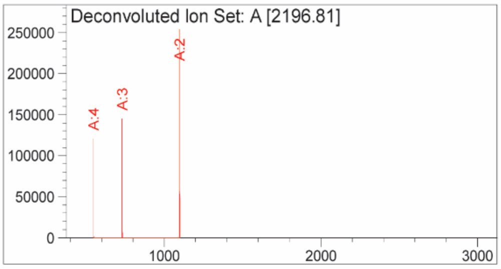

Component	Molecular Weight	Absolute Abundance	Relative Abundance
A	2196.81	515783	100.00

*** End of Report ***

*U*G*C*G*U*G*U

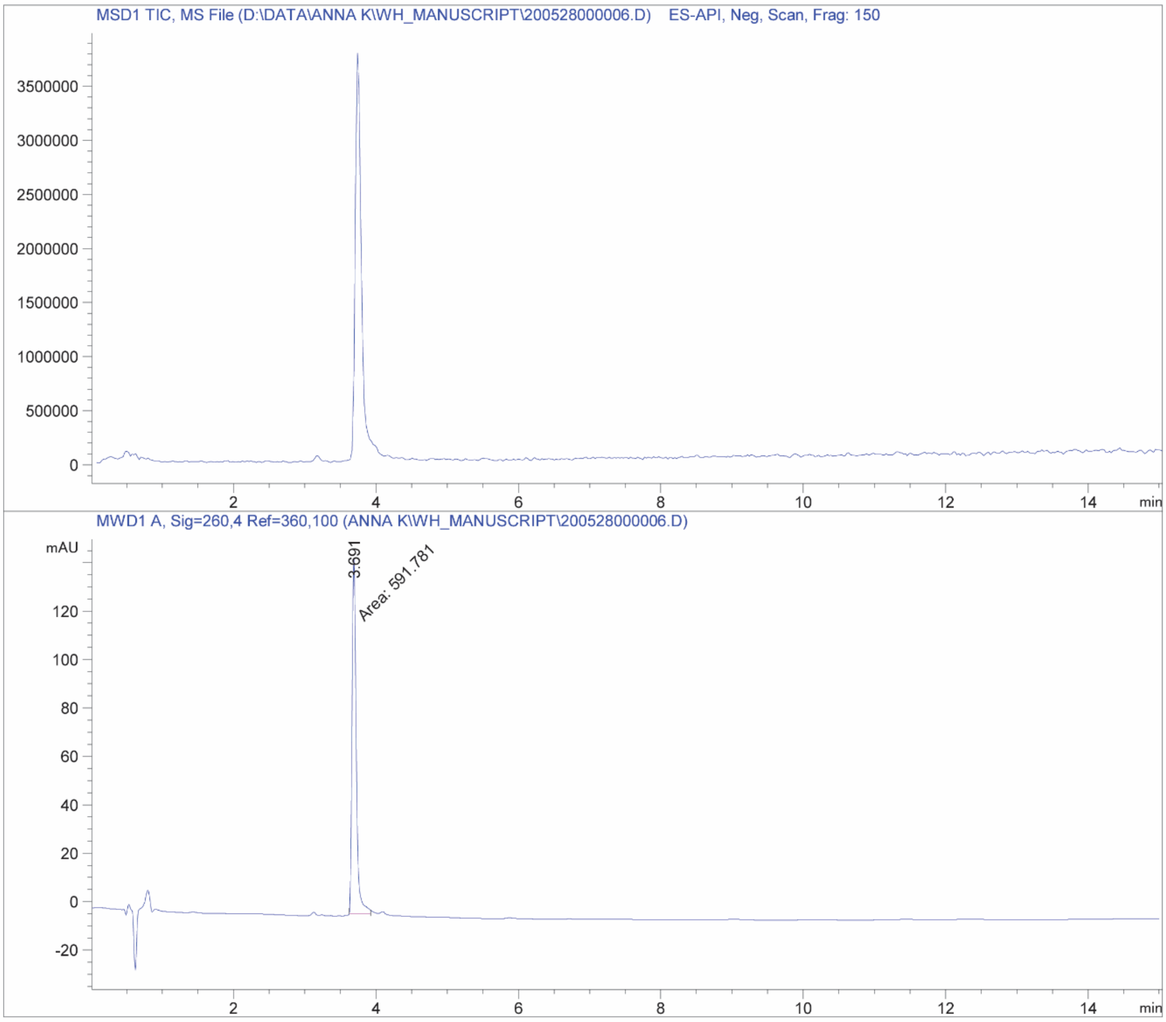

Deconvolution of Spectrum # 1 @ 3.439 - 3.937 min

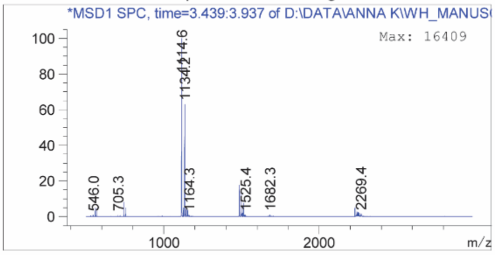

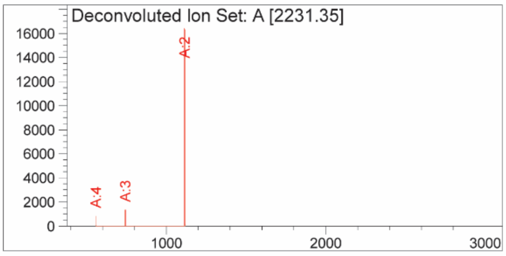

Signal 2: MWD1 A, Sig=260,4 Ref=360,100

Peak #	RetTime [min]	Type	Width [min]	Area [mAU*s]	Height [mAU]	Area %
1	3.691	PM	0.0670	591.78113	147.14174	100.0000
Totals :				591.78113	147.14174	

Component	Molecular Weight	Absolute Abundance	Relative Abundance
A	2231.35	18566	100.00
B	2270.37	11899	64.09

UGC*GUGU

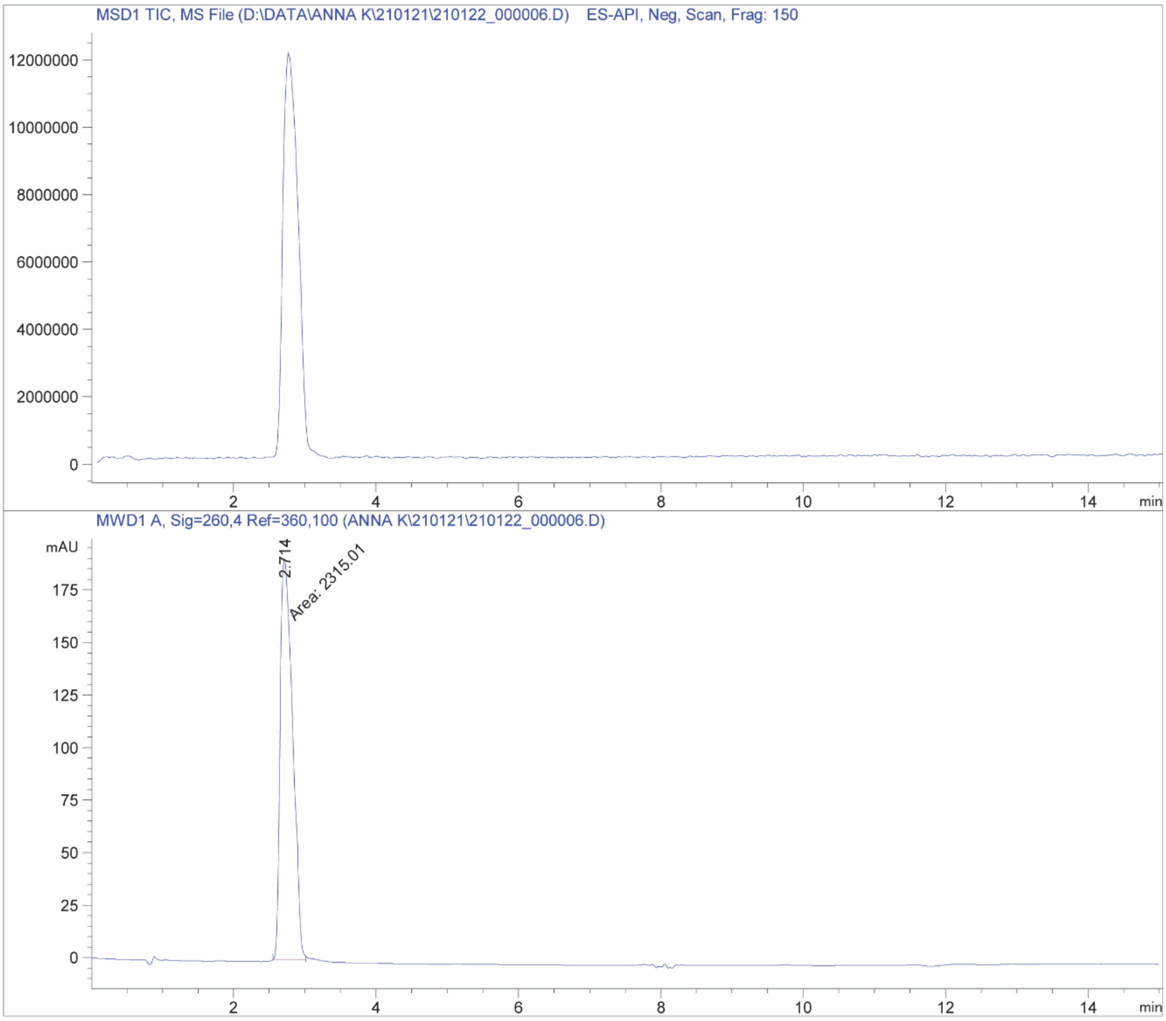

Signal 2: MWD1 A, Sig=260,4 Ref=360,100

Peak #	RetTime [min]	Type	Width [min]	Area [mAU*s]	Height [mAU]	Area %
1	2.714	MM	0.2022	2315.01050	190.84366	100.0000

Totals : 2315.01050 190.84366

Deconvolution of Spectrum # 1 @ 2.617 - 2.990 min

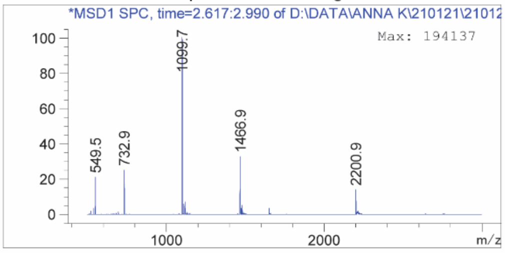

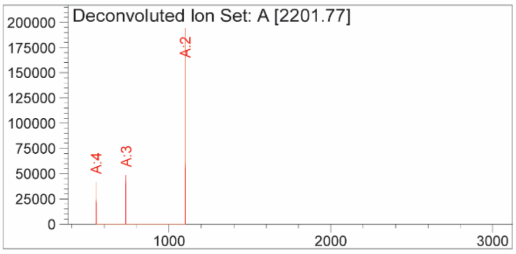

Component	Molecular Weight	Absolute Abundance	Relative Abundance
A	2201.77	281594	100.00

*** End of Report ***

UGCAGGU

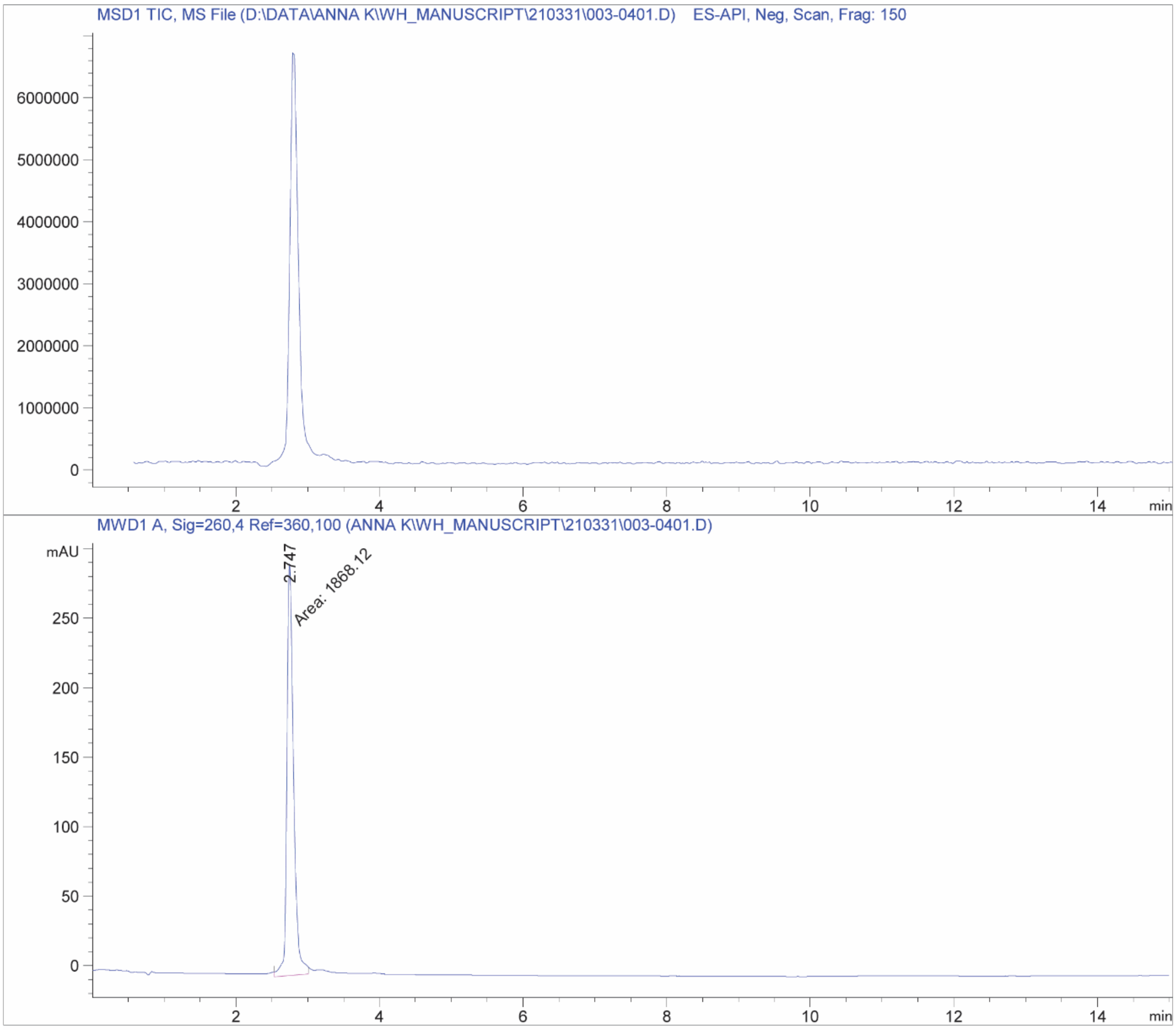

Signal 2: MWD1 A, Sig=260,4 Ref=360,100

Peak #	RetTime [min]	Type	Width [min]	Area [mAU*s]	Height [mAU]	Area %
1	2.747	MM	0.1048	1868.12463	297.00012	100.0000
Totals :				1868.12463	297.00012	

Deconvolution of Spectrum # 1 @ 2.567 - 3.065 min

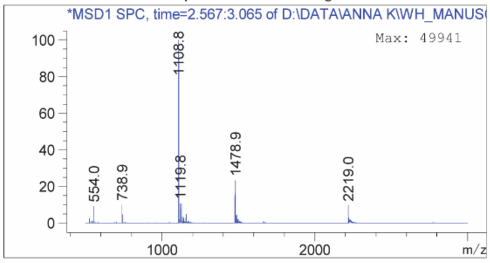

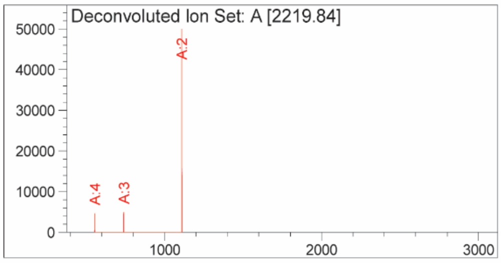

Component	Molecular Weight	Absolute Abundance	Relative Abundance
A	2219.84	59224	100.00

*** End of Report ***

*U*G*C*A*G*G*U

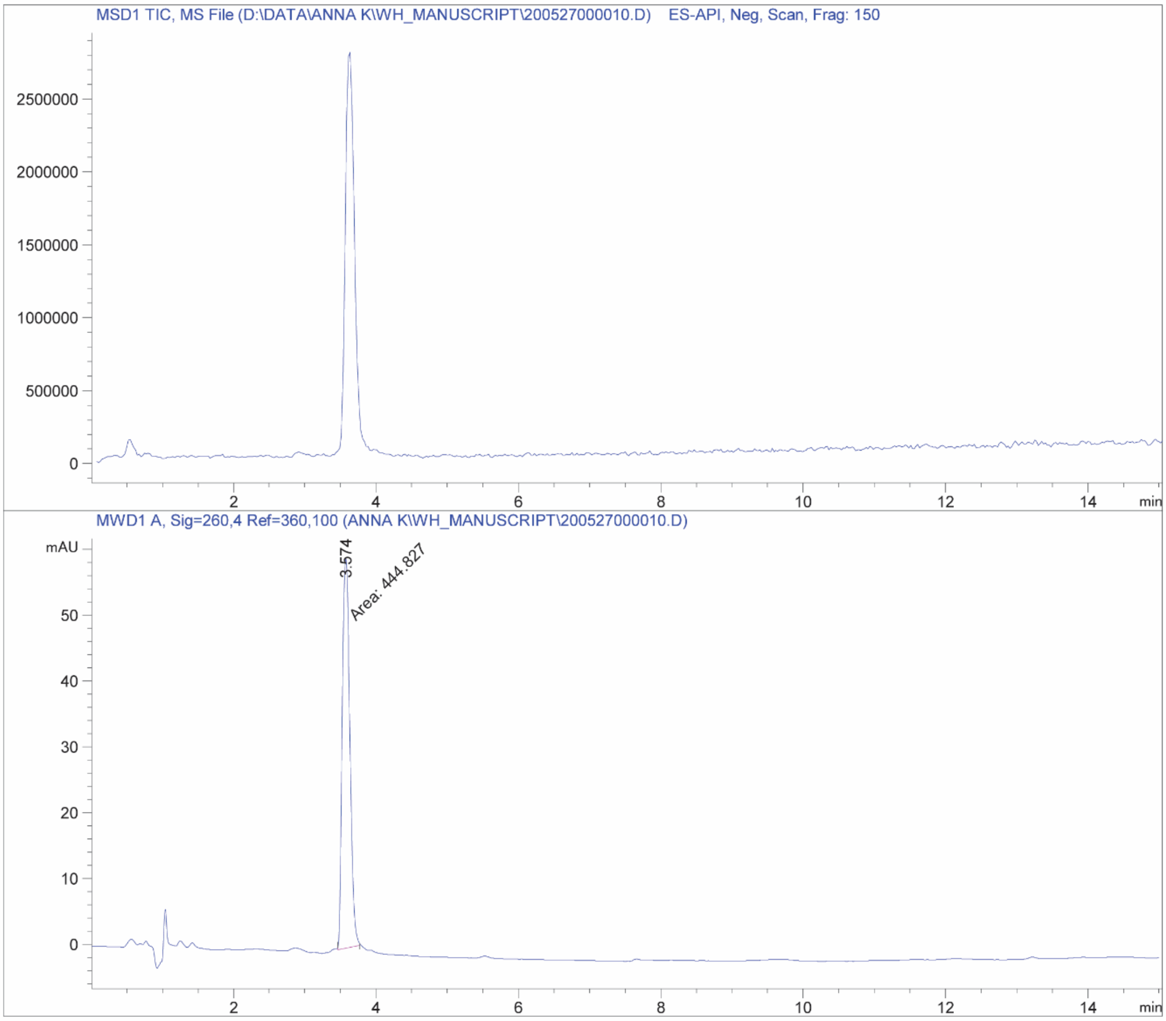

Signal 2: MWD1 A, Sig=260,4 Ref=360,100

Peak #	RetTime [min]	Type	Width [min]	Area [mAU*s]	Height [mAU]	Area %
1	3.574	MM	0.1251	444.82666	59.24973	100.0000

Totals : 444.82666 59.24973

Deconvolution of Spectrum # 1 @ 3.588 - 3.713 min

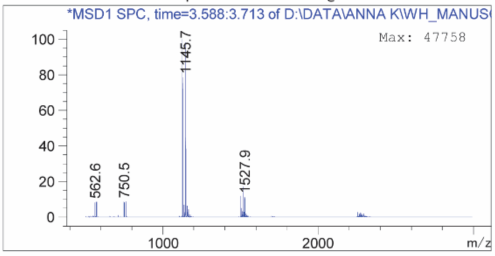

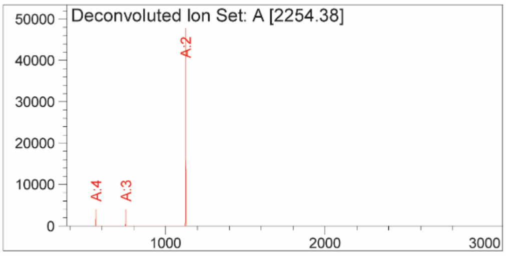

Component	Molecular Weight	Absolute Abundance	Relative Abundance
A	2254.38	55655	100.00
B	2293.37	54977	98.78

UGCA*GGU

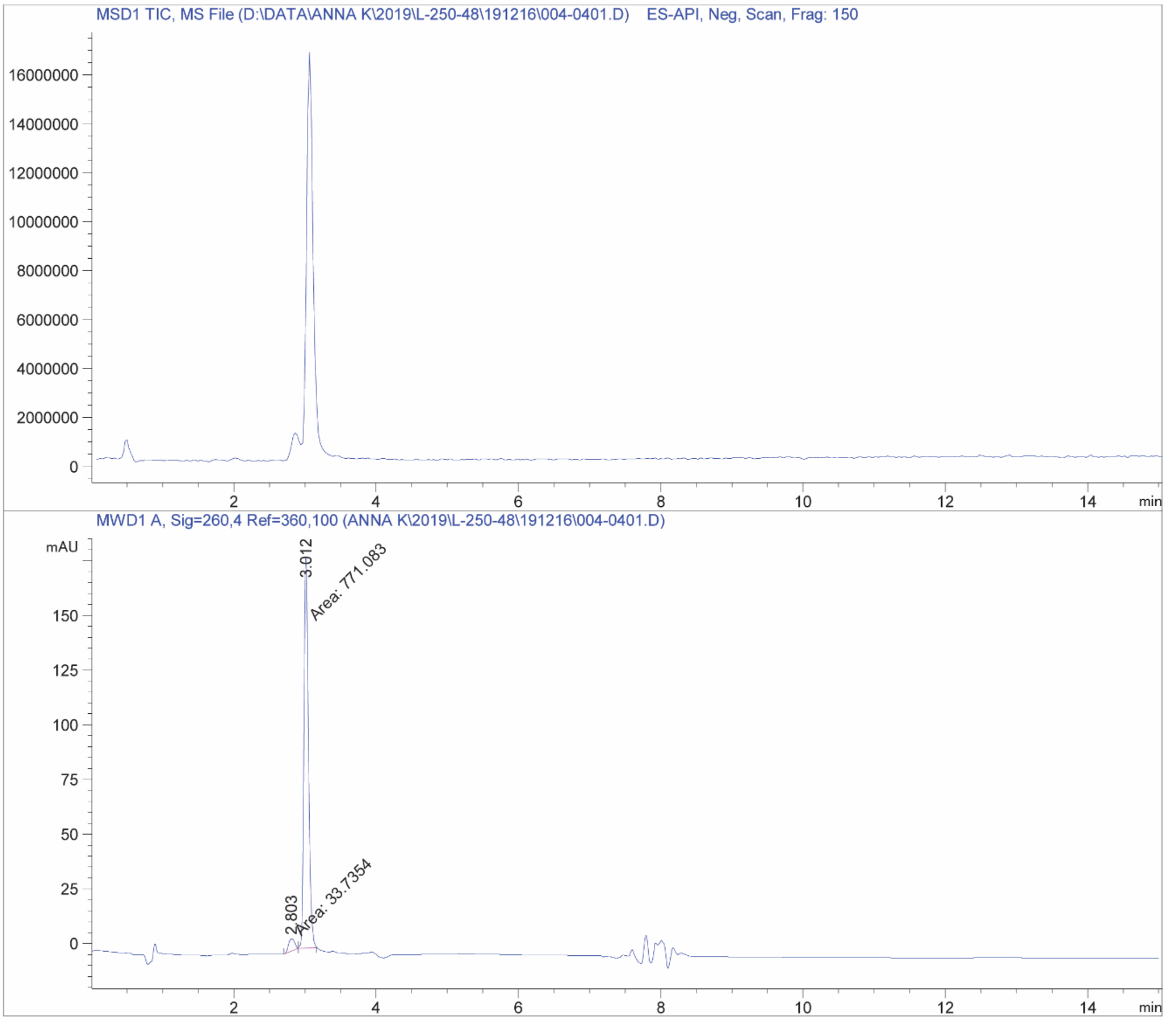

Deconvolution of Spectrum # 1 @ 2.866 - 3.090 min

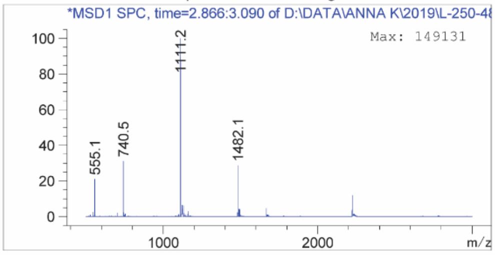

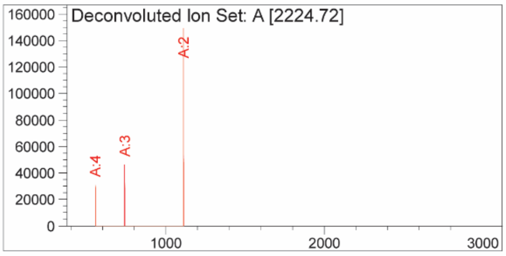

Signal 2: MWD1 A, Sig=260,4 Ref=360,100

Peak #	RetTime [min]	Type	Width [min]	Area [mAU*s]	Height [mAU]	Area %
1	2.803	MM T	0.1164	33.73536	5.55452	4.1917
2	3.012	MM T	0.0715	771.08350	179.77684	95.8083

Component	Molecular Weight	Absolute Abundance	Relative Abundance
A	2224.72	226448	100.00

UGCAUGG

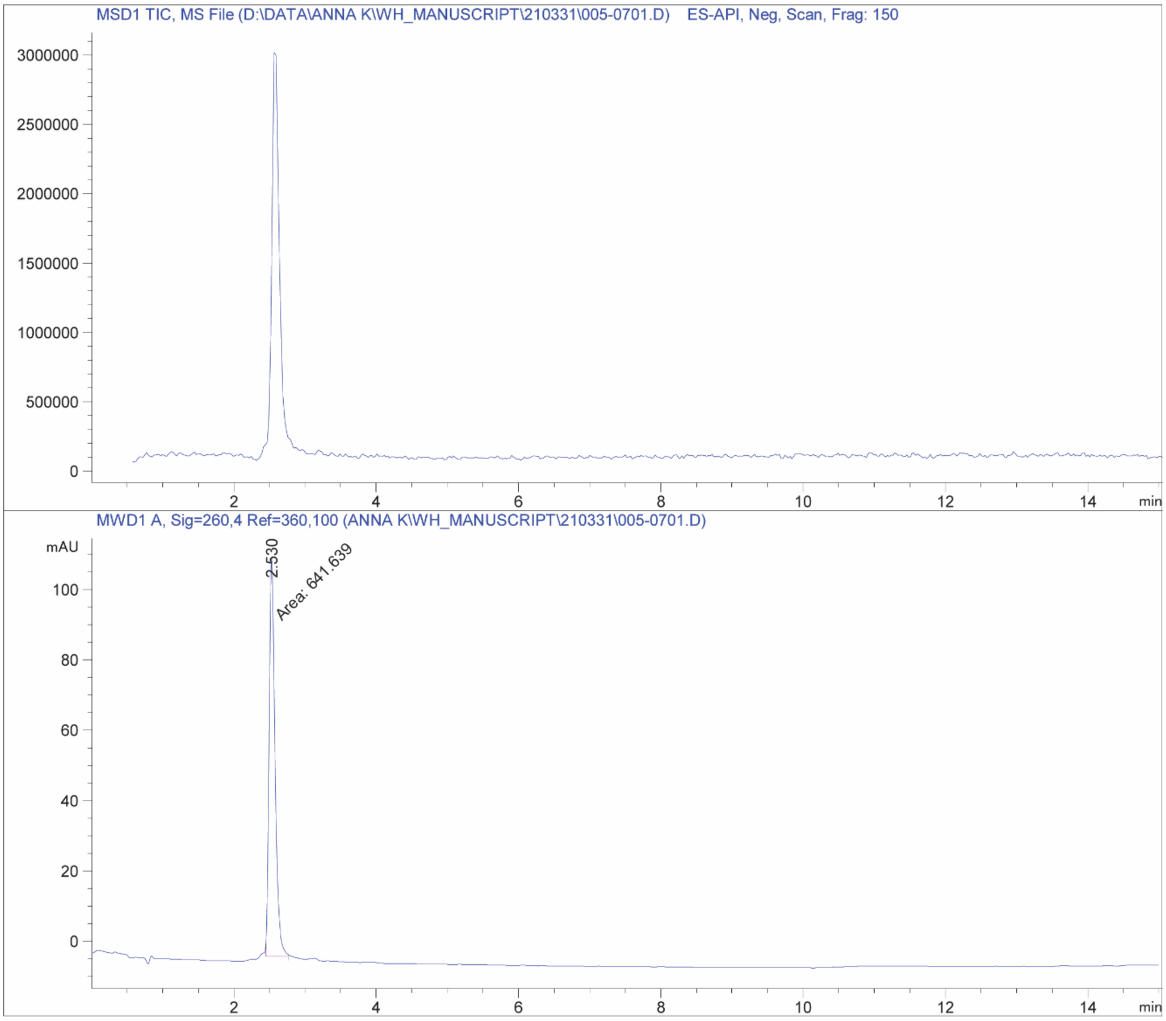

Deconvolution of Spectrum # 1 @ 2.417 - 2.766 min

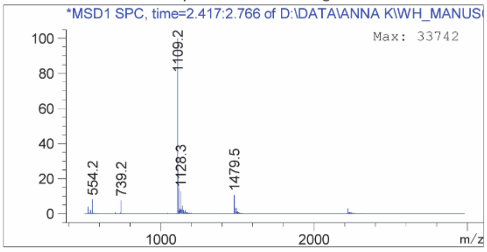

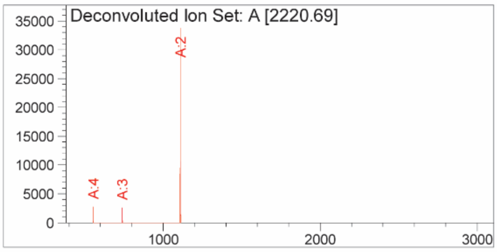

Signal 2: MWD1 A, Sig=260,4 Ref=360,100

Peak #	RetTime [min]	Type	Width [min]	Area [mAU*s]	Height [mAU]	Area %
1	2.530	MM	0.0944	641.63867	113.32418	100.0000
Totals :				641.63867	113.32418	

Component	Molecular Weight	Absolute Abundance	Relative Abundance
A	2220.69	38867	100.00

*** End of Report ***

*U*G*C*A*U*G*G

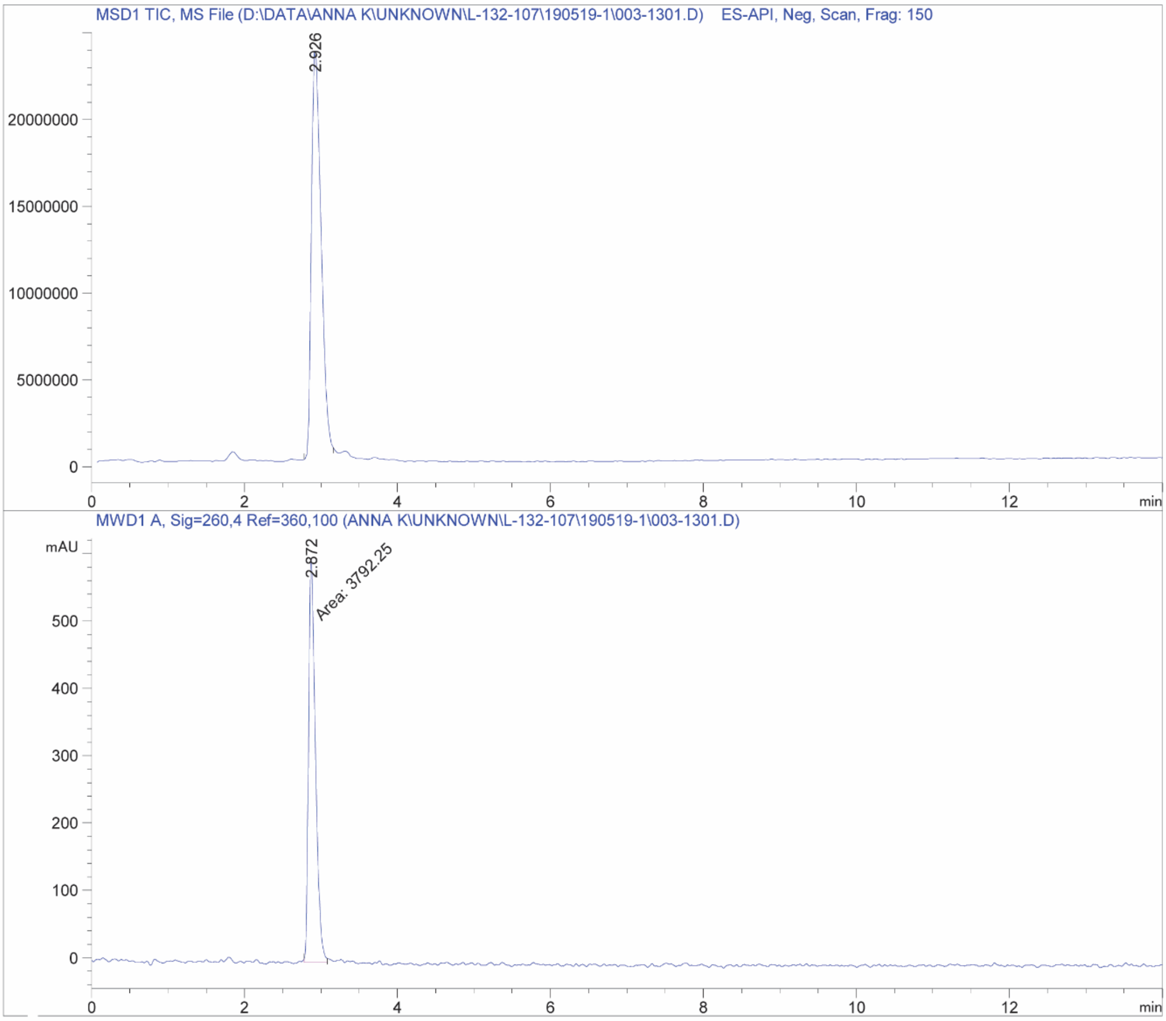

Signal 2: MWD1 A, Sig=260,4 Ref=360,100

Peak #	RetTime [min]	Type	Width [min]	Area [mAU*s]	Height [mAU]	Area %
1	2.872	MM	0.1048	3792.24561	603.00836	100.0000
Totals :				3792.24561	603.00836	

Deconvolution of Spectrum #   1 @ 2.816 - 3.015 min

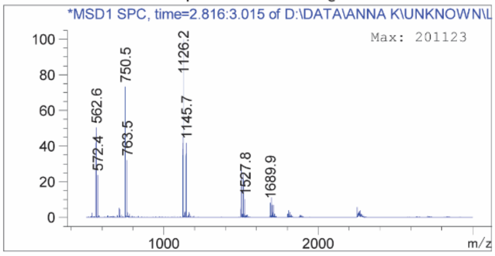

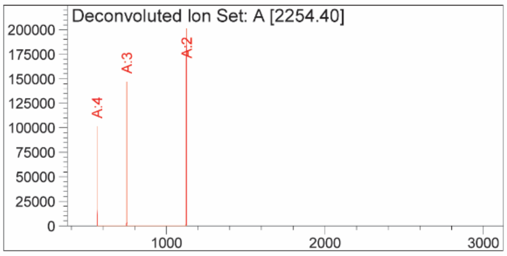

Component	Molecular Weight	Absolute Abundance	Relative Abundance
A	2254.40	447041	100.00
B	2293.38	194438	43.49

UGCAUG*G

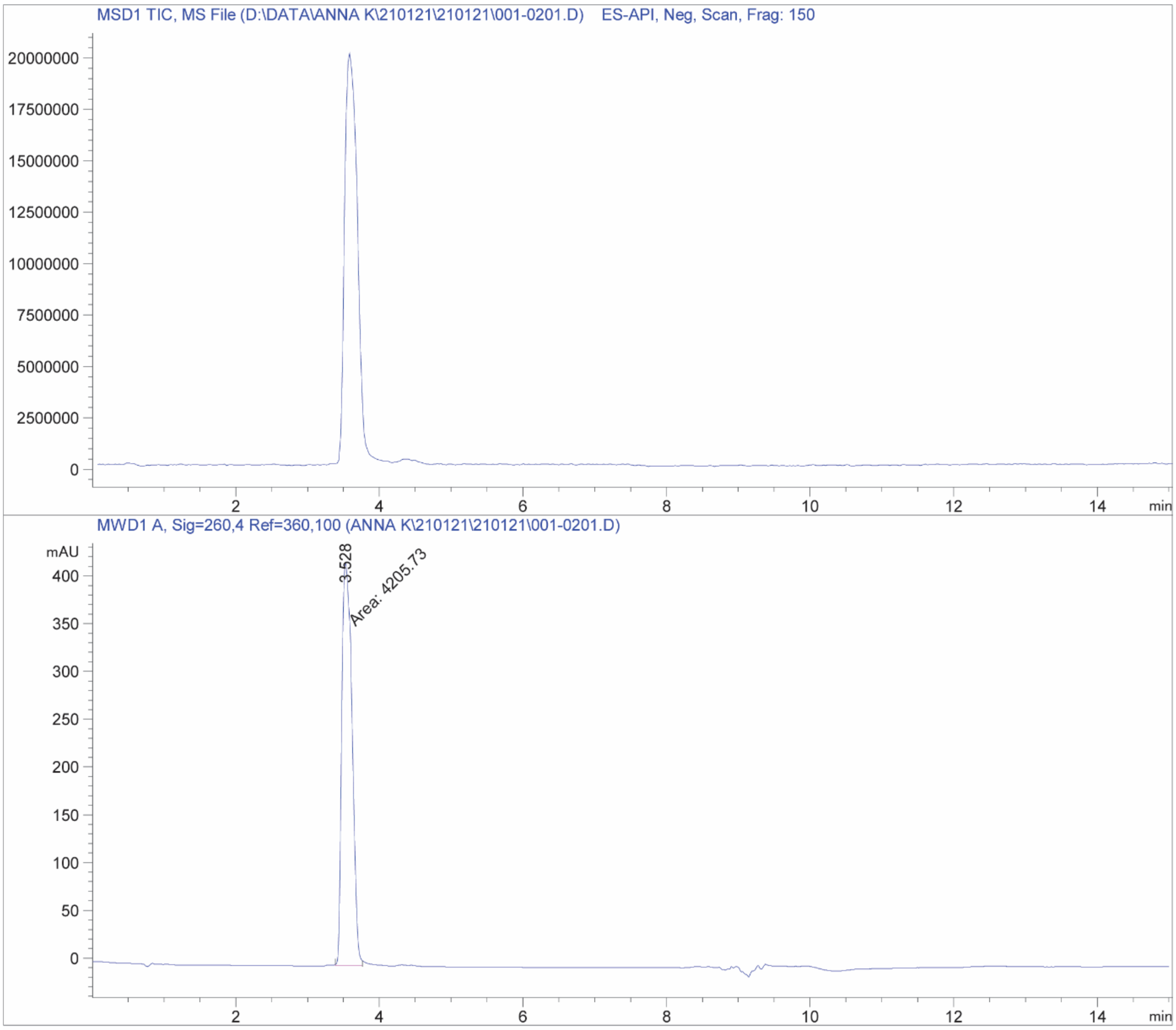

Signal 2: MWD1 A, Sig=260,4 Ref=360,100

Peak #	RetTime [min]	Type	Width [min]	Area [mAU*s]	Height [mAU]	Area %
1	3.528	MM	0.1665	4205.72510	420.95566	100.0000

Totals : 4205.72510 420.95566

Deconvolution of Spectrum # 1 @ 3.414 - 3.788 min

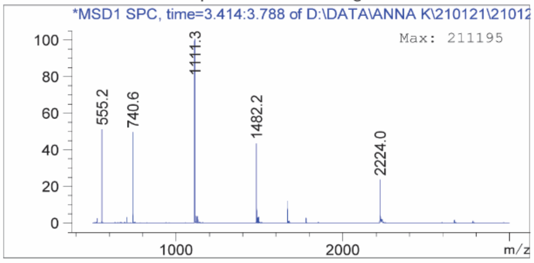

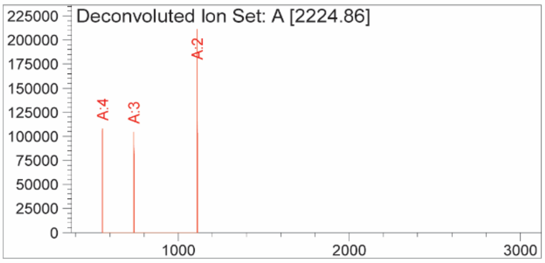

Component	Molecular Weight	Absolute Abundance	Relative Abundance
A	2224.86	419647	100.00

*** End of Report ***

UGUAUGU

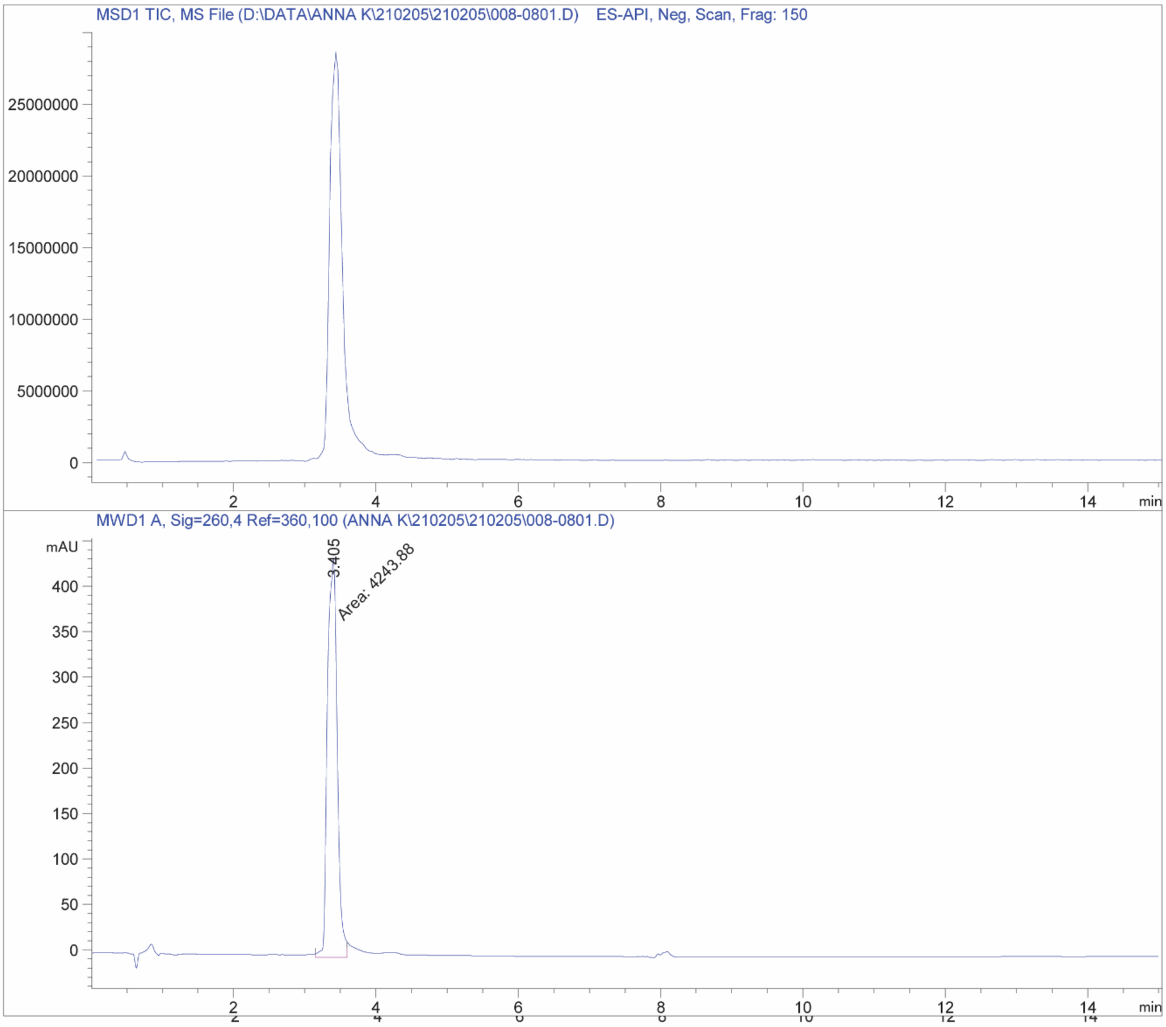

Deconvolution of Spectrum # 1 @ 3.165 - 3.588 min

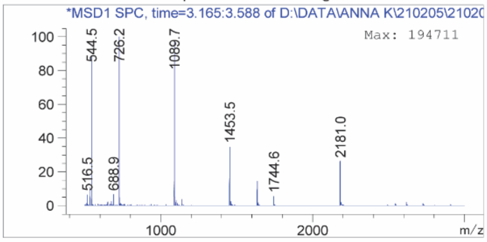

Signal 2: MWD1 A, Sig=260,4 Ref=360,100

Peak #	RetTime [min]	Type	Width [min]	Area [mAU*s]	Height [mAU]	Area %
1	3.405	MM	0.1611	4243.88281	438.92560	100.0000
Totals :				4243.88281	438.92560	

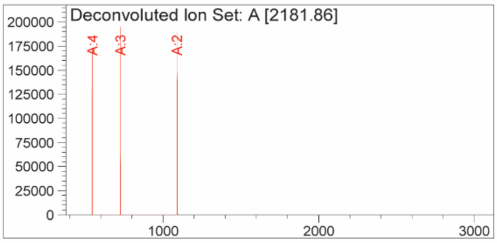

Component	Molecular Weight	Absolute Abundance	Relative Abundance
A	2181.86	552614	100.00

*** End of Report ***

*U*G*U*A*U*G*U

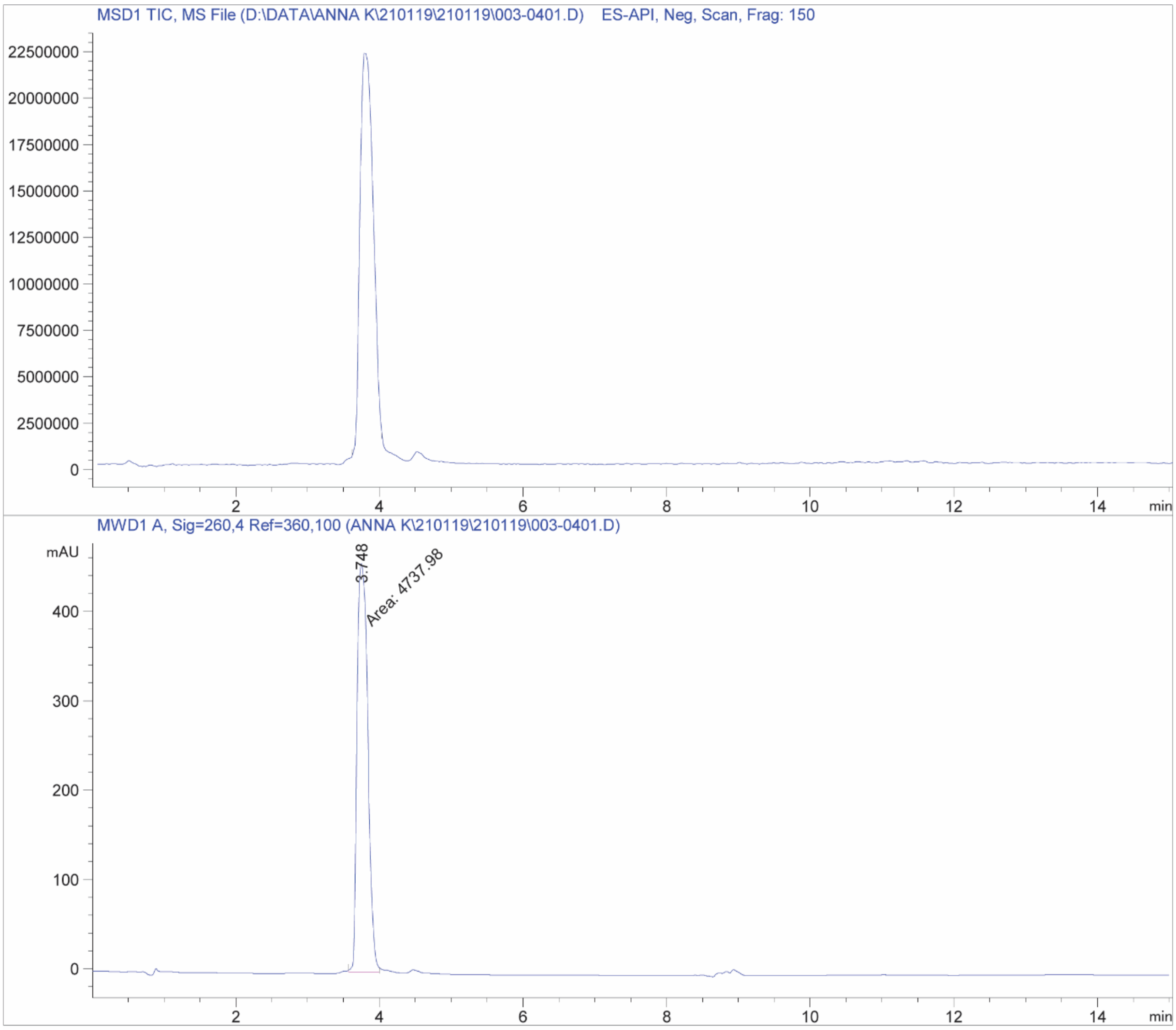

Signal 2: MWD1 A, Sig=260,4 Ref=360,100

Peak #	RetTime [min]	Type	Width [min]	Area [mAU*s]	Height [mAU]	Area %
1	3.748	MM	0.1727	4737.97803	457.37164	100.0000
Totals :				4737.97803	457.37164	

Deconvolution of Spectrum # 1 @ 3.788 min

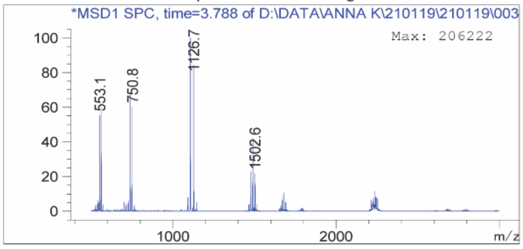

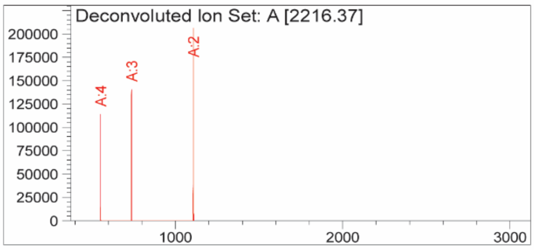

Component	Molecular Weight	Absolute Abundance	Relative Abundance
A	2216.37	461096	100.00
B	2255.42	420848	91.27

*** End of Report ***

UG*UAUGU

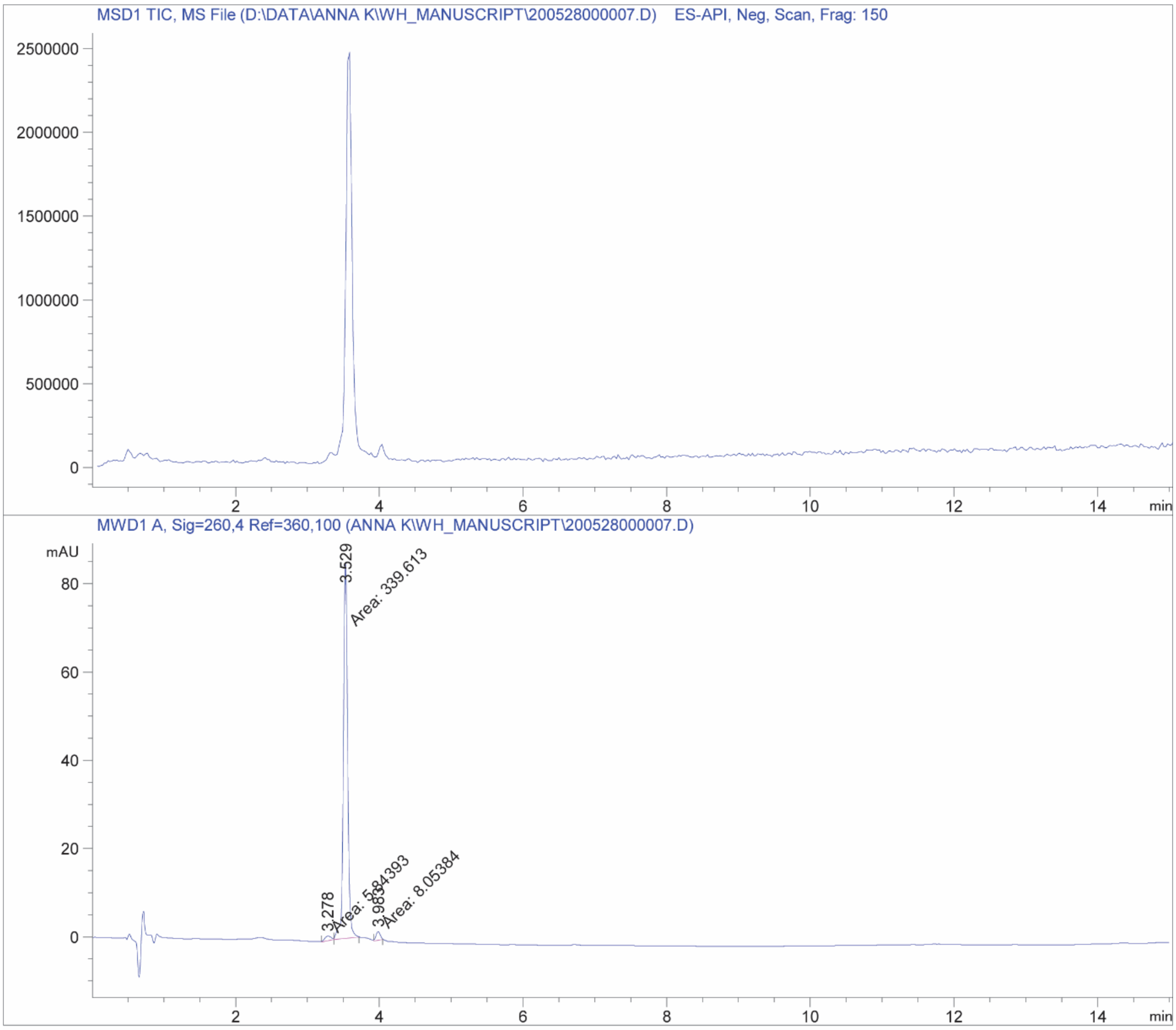

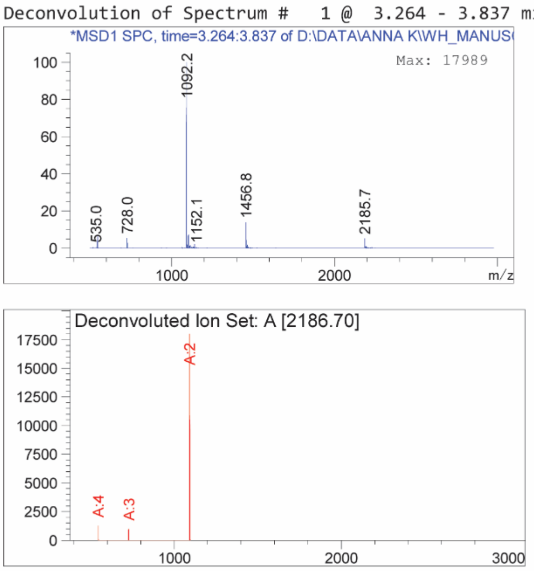

Signal 2: MWD1 A, Sig=260,4 Ref=360,100

Peak #	RetTime [min]	Type	Width [min]	Area [mAU*s]	Height [mAU]	Area %
1	3.278	MM	0.0982	5.84393	9.91948e-1	1.6531
2	3.529	MP	0.0663	339.61255	85.37032	96.0686
3	3.983	MM	0.0664	8.05384	2.02238	2.2782
Totals :				353.51032	88.38464	

Component	Molecular Weight	Absolute Abundance	Relative Abundance
A	2186.70	20069	100.00

*** End of Report ***

UGCUUGU

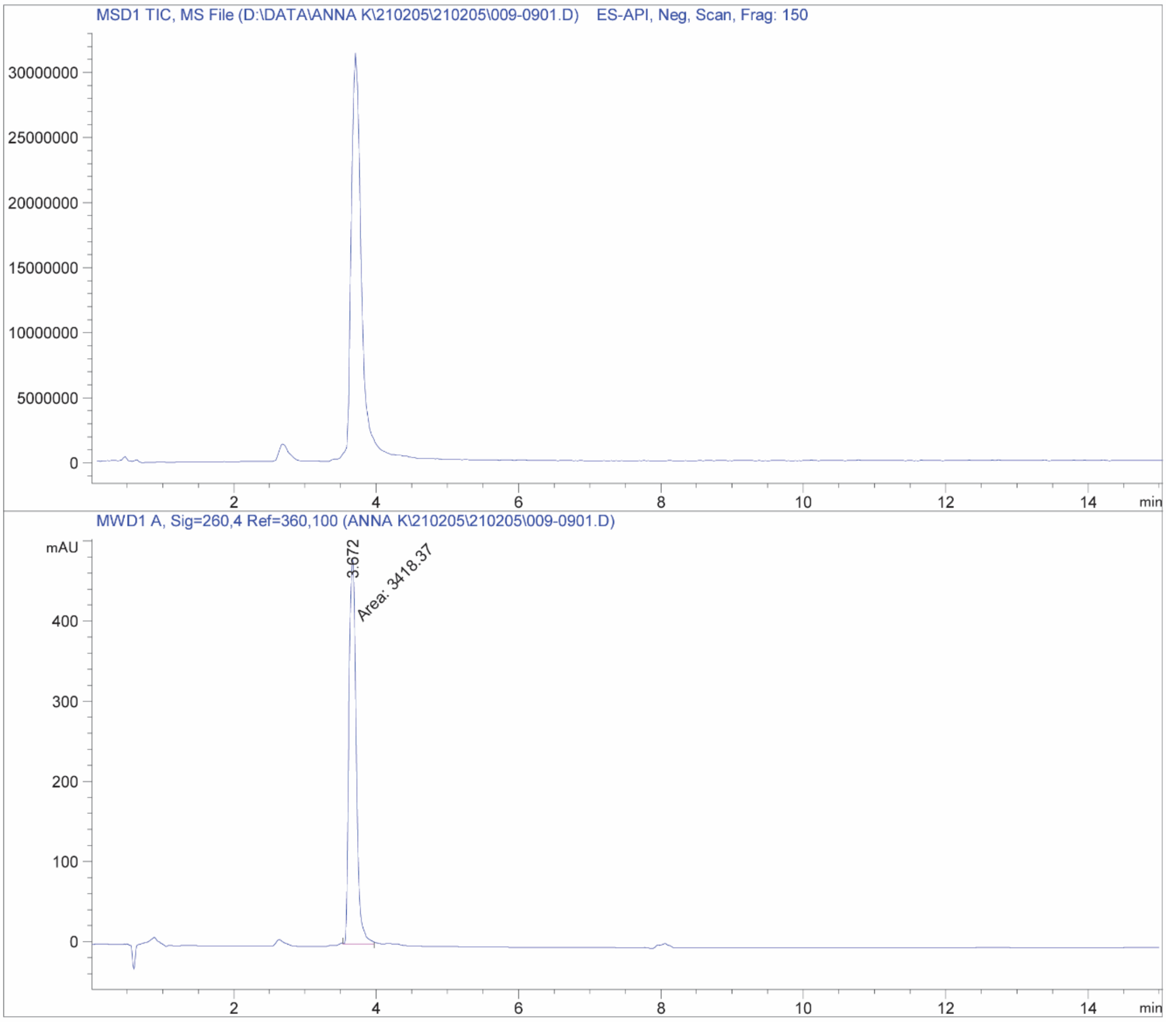

Signal 2: MWD1 A, Sig=260,4 Ref=360,100

Peak #	RetTime [min]	Type	Width [min]	Area [mAU*s]	Height [mAU]	Area %
1	3.672	MM	0.1185	3418.36743	480.75742	100.0000

Totals :	3418.36743	480.75742

Deconvolution of Spectrum # 1 @ 3.414 - 3.937 min

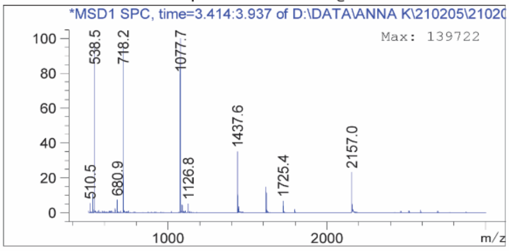

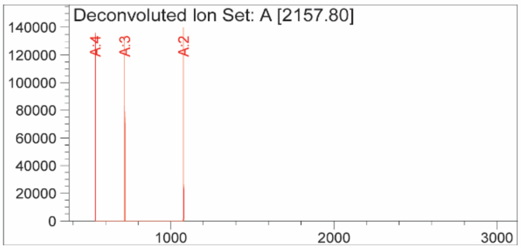

Component	Molecular Weight	Absolute Abundance	Relative Abundance
A	2157.80	405378	100.00

*** End of Report ***

*U*G*C*U*U*G*U

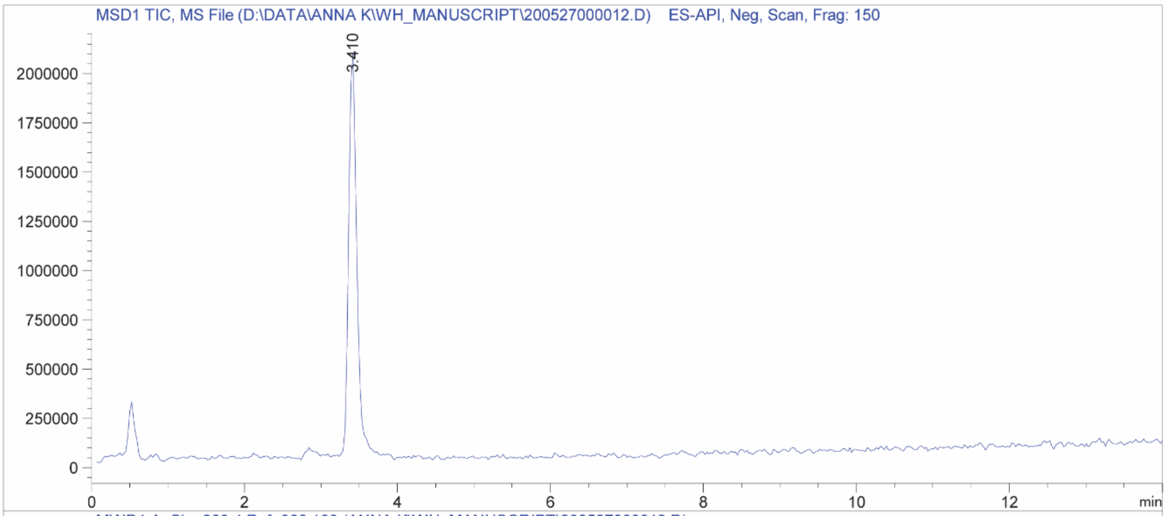

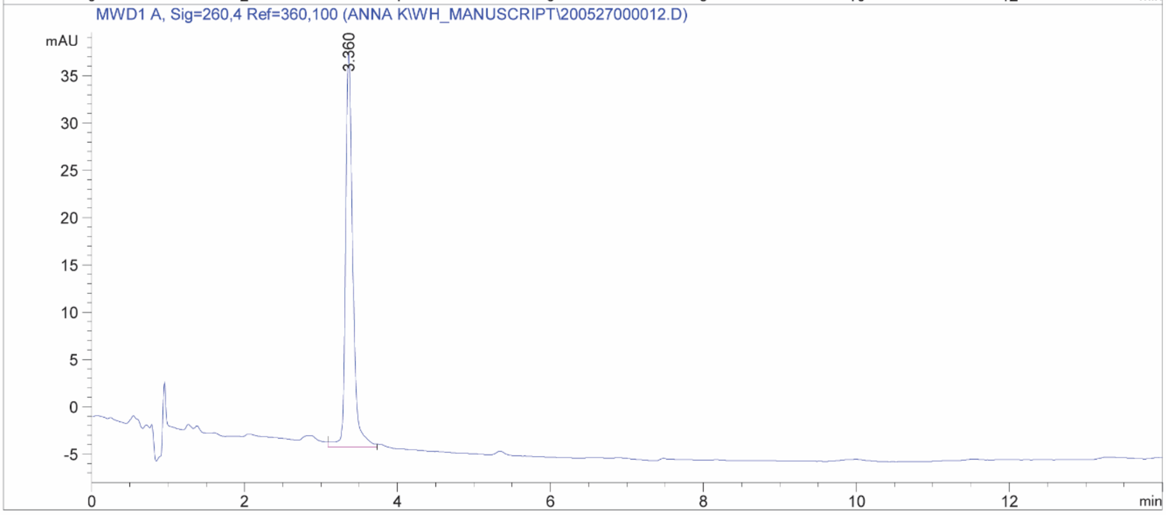

Signal 2: MWD1 A, Sig=260,4 Ref=360,100

Peak #	RetTime [min]	Type	Width [min]	Area [mAU*s]	Height [mAU]	Area %
1	3.360	VB	0.1019	279.36246	41.84702	100.0000
Totals :				279.36246	41.84702	

Deconvolution of Spectrum # 1 @ 3.240 - 3.688 min

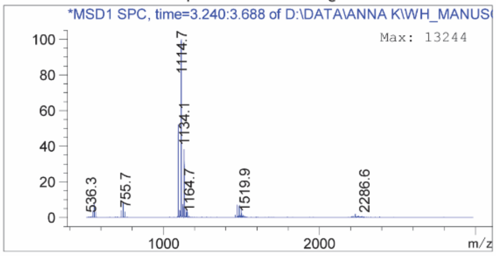

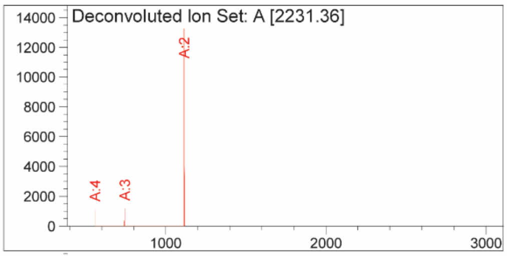

Component	Molecular Weight	Absolute Abundance	Relative Abundance
A	2231.36	15507	100.00
B	2192.27	7898	50.93
C	2270.23	5934	38.27

UGC*UUGU

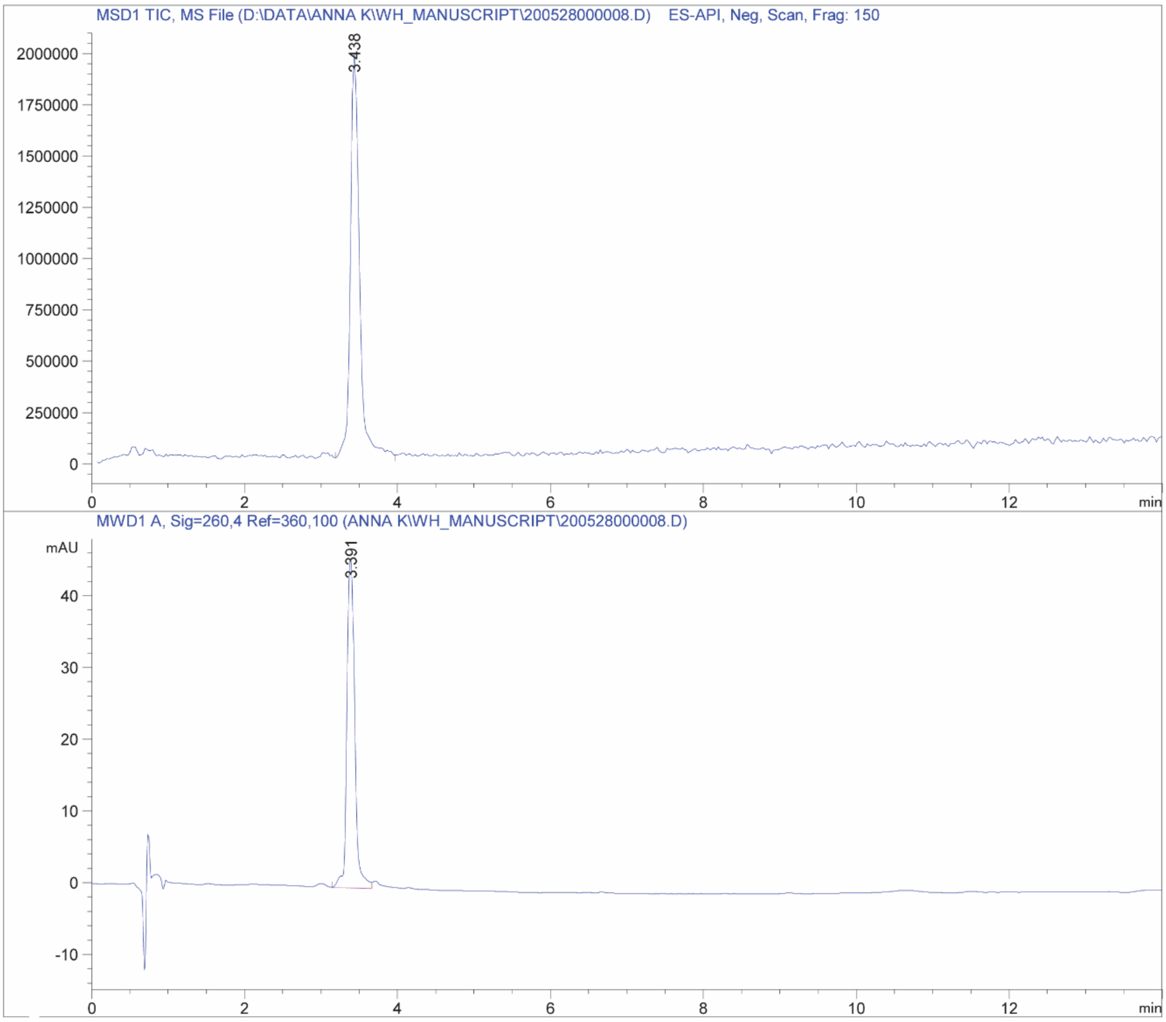

Signal 2: MWD1 A, Sig=260,4 Ref=360,100

Peak #	RetTime [min]	Type	Width [min]	Area [mAU*s]	Height [mAU]	Area %
1	3.391	VV	0.1128	325.19199	45.79882	100.0000

Totals : 325.19199 45.79882

Deconvolution of Spectrum # 1 @ 3.264 - 3.613 min

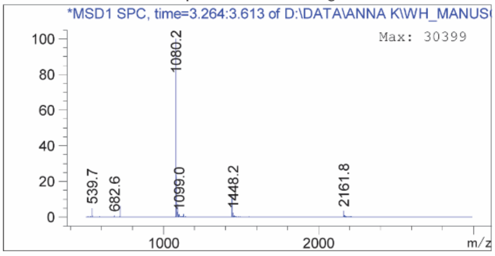

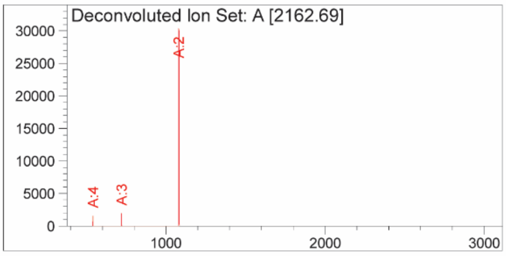

Component	Molecular Weight	Absolute Abundance	Relative Abundance
A	2162.69	33765	100.00

*** End of Report ***

UACAUGU

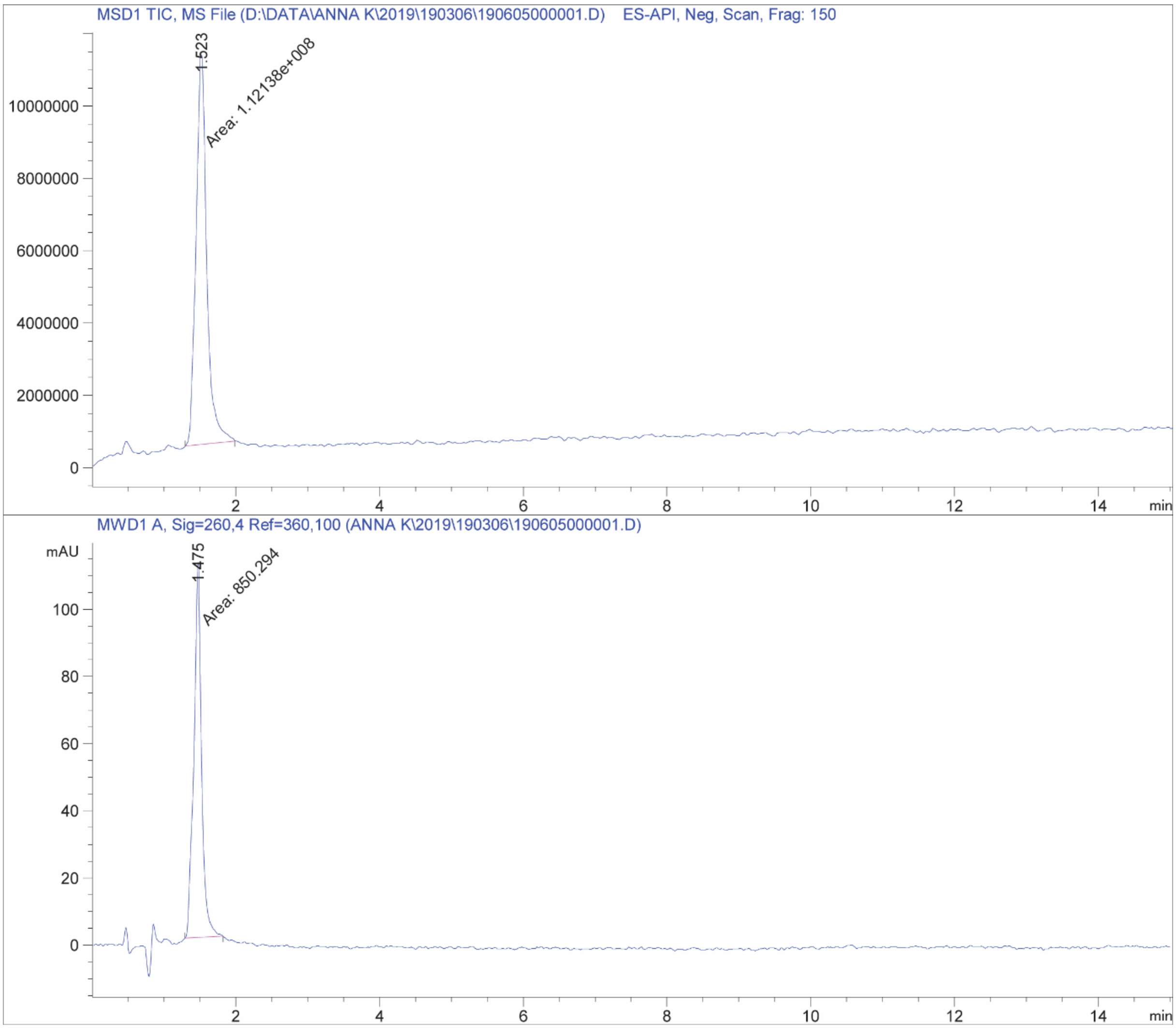

Signal 2: MWD1 A, Sig=260,4 Ref=360,100

Peak #	RetTime [min]	Type	Width [min]	Area [mAU*s]	Height [mAU]	Area %
1	1.475	MM	0.1269	850.29419	111.67923	100.0000
Totals :				850.29419	111.67923	

Deconvolution of Spectrum # 1 @ 1.412 - 1.611 min

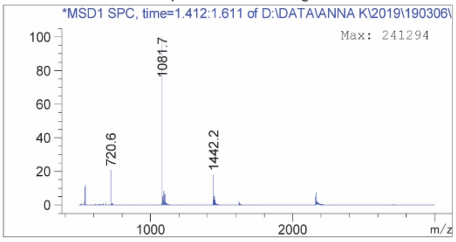

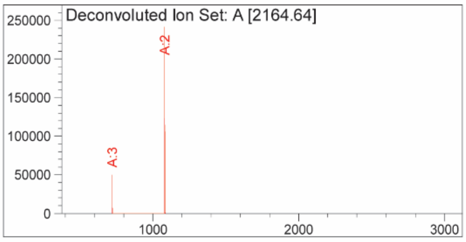

Component	Molecular Weight	Absolute Abundance	Relative Abundance
A	2164.64	285979	100.00

*** End of Report ***

*U*A*C*A*U*G*U

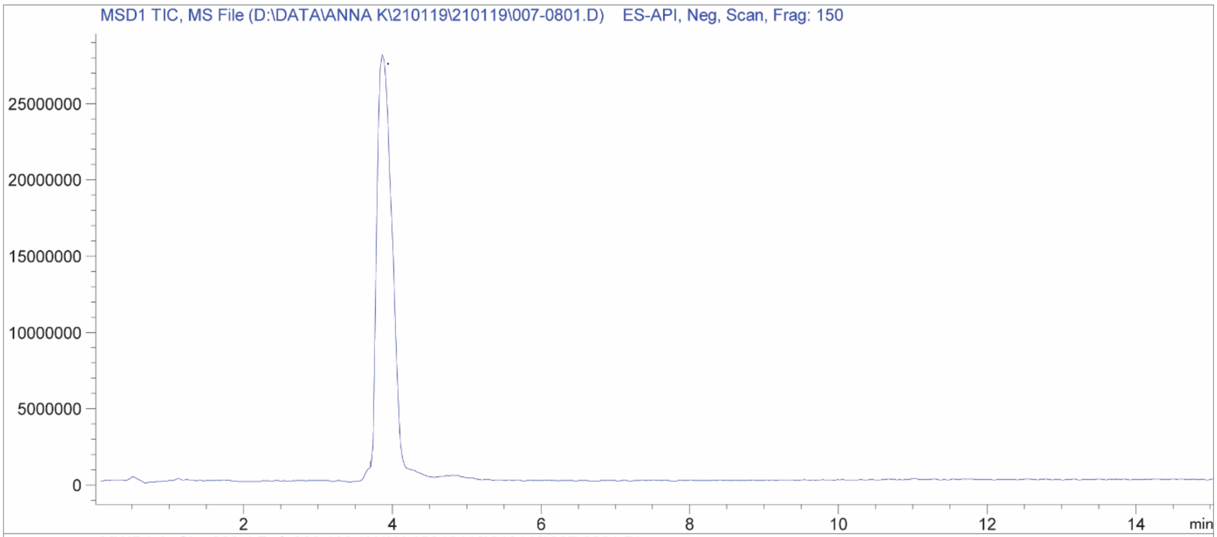

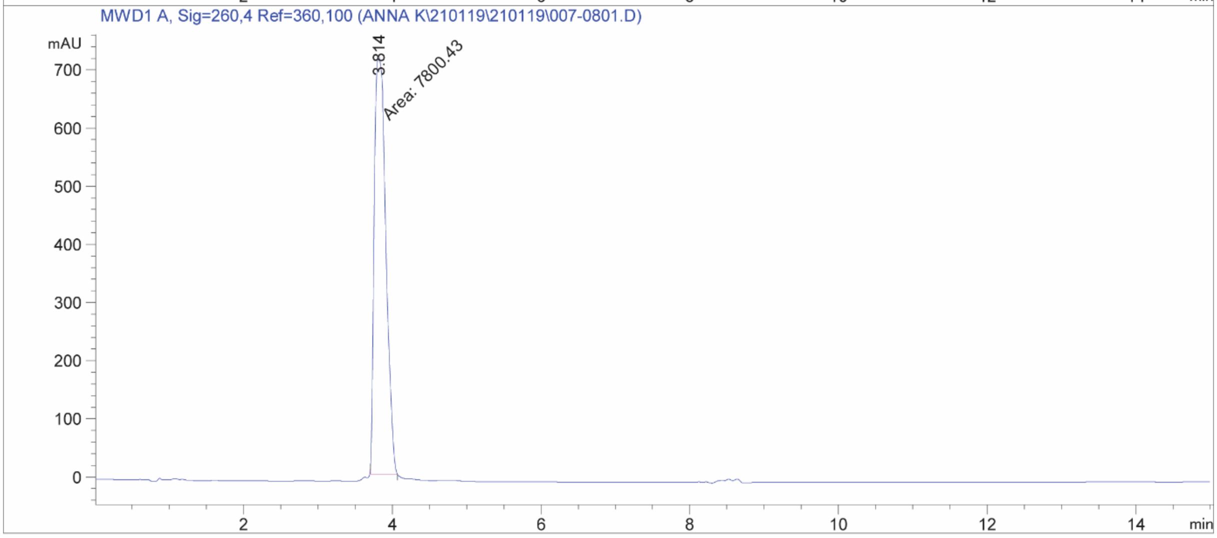

Signal 2: MWD1 A, Sig=260,4 Ref=360,100

Peak #	RetTime [min]	Type	Width [min]	Area [mAU*s]	Height [mAU]	Area %
1	3.814	MM	0.1807	7800.42871	719.45087	100.0000

Totals :	7800.42871	719.45087

Deconvolution of Spectrum # 1 @ 3.763 - 3.987 min

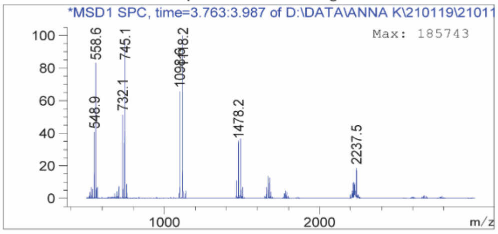

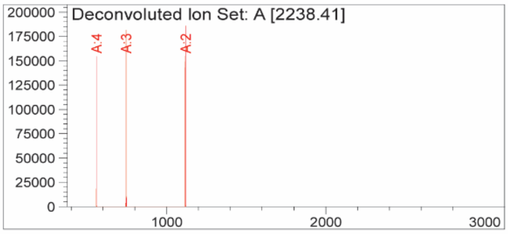

Component	Molecular Weight	Absolute Abundance	Relative Abundance
A	2238.41	520796	100.00
B	2199.35	290828	55.84

*** End of Report ***

UGCAUAU

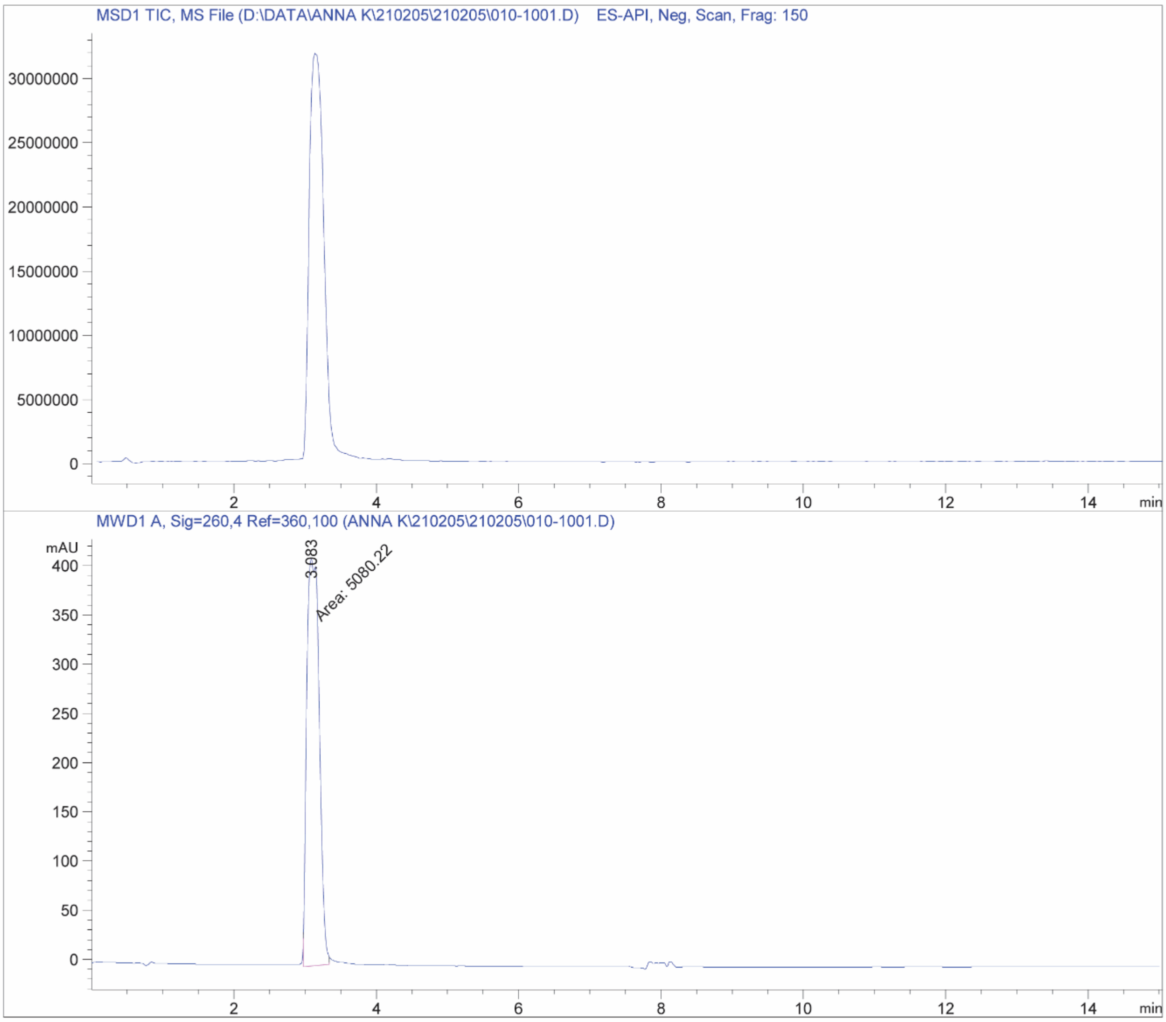

Signal 2: MWD1 A, Sig=260,4 Ref=360,100

Peak #	RetTime [min]	Type	Width [min]	Area [mAU*s]	Height [mAU]	Area %
1	3.083	MM	0.2049	5080.22119	413.28690	100.0000

Totals : 5080.22119 413.28690

Deconvolution of Spectrum # 1 @ 2.916 - 3.414 min

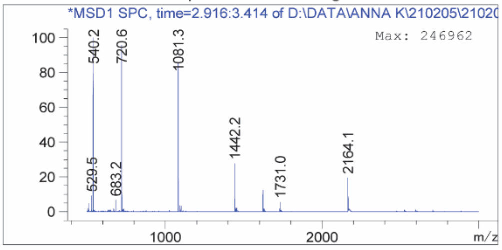

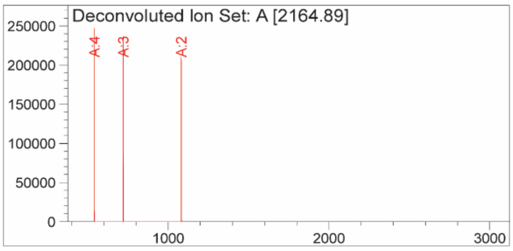

Component	Molecular Weight	Absolute Abundance	Relative Abundance
A	2164.89	687566	100.00

*** End of Report ***

*U*G*C*A*U*A*U

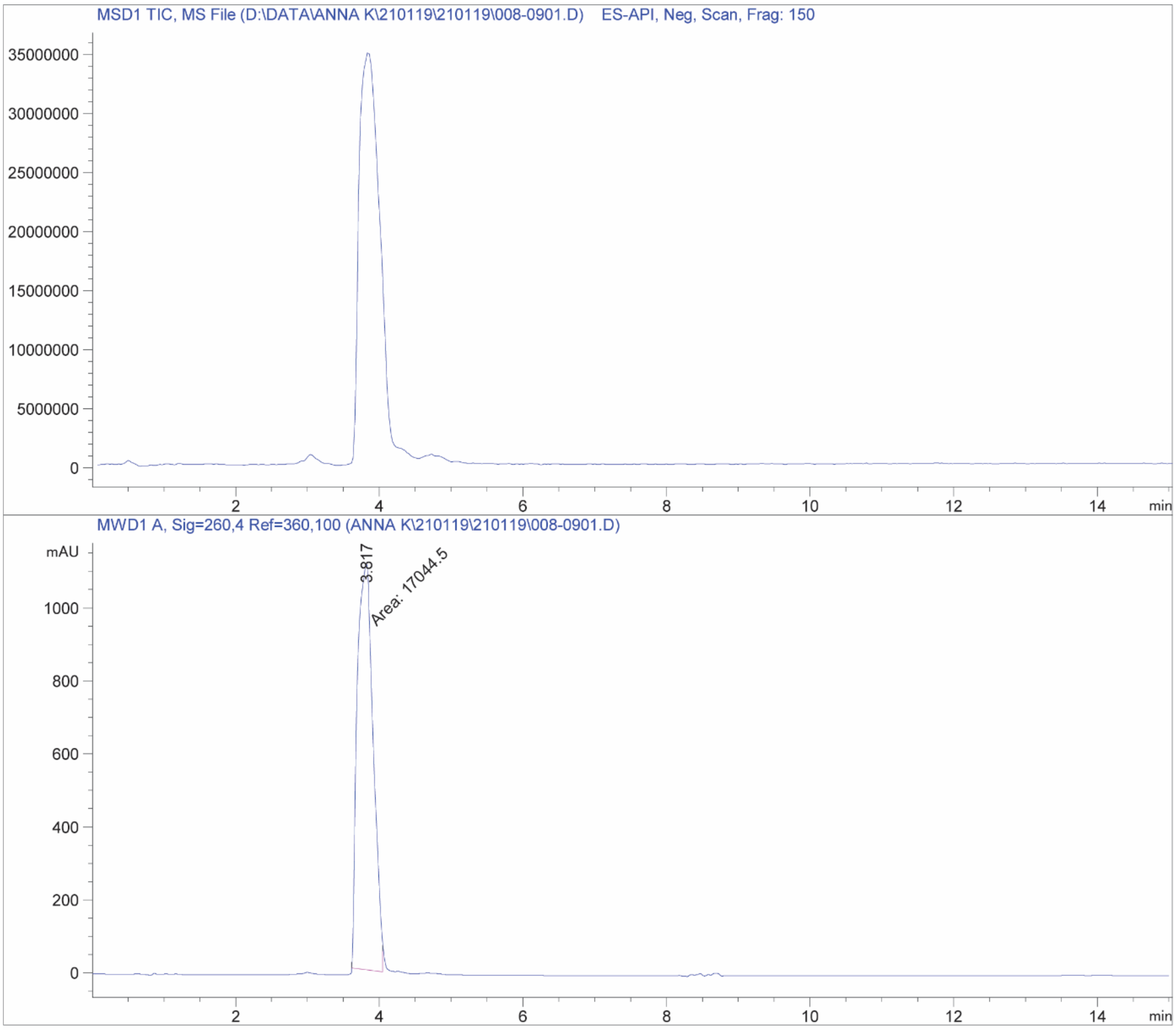

Signal 2: MWD1 A, Sig=260,4 Ref=360,100

Peak #	RetTime [min]	Type	Width [min]	Area [mAU*s]	Height [mAU]	Area %
1	3.817	PM	0.2552	1.70445e4	1113.05896	100.0000

Totals : 1.70445e4 1113.05896

Deconvolution of Spectrum # 1 @ 3.837 min

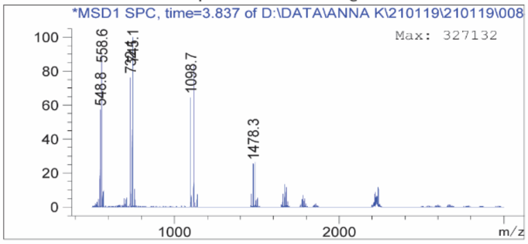

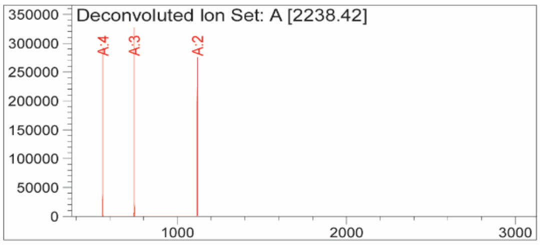

Component	Molecular Weight	Absolute Abundance	Relative Abundance
A	2238.42	890911	100.00
B	2199.38	646850	72.61

*** End of Report ***

UACAUAU

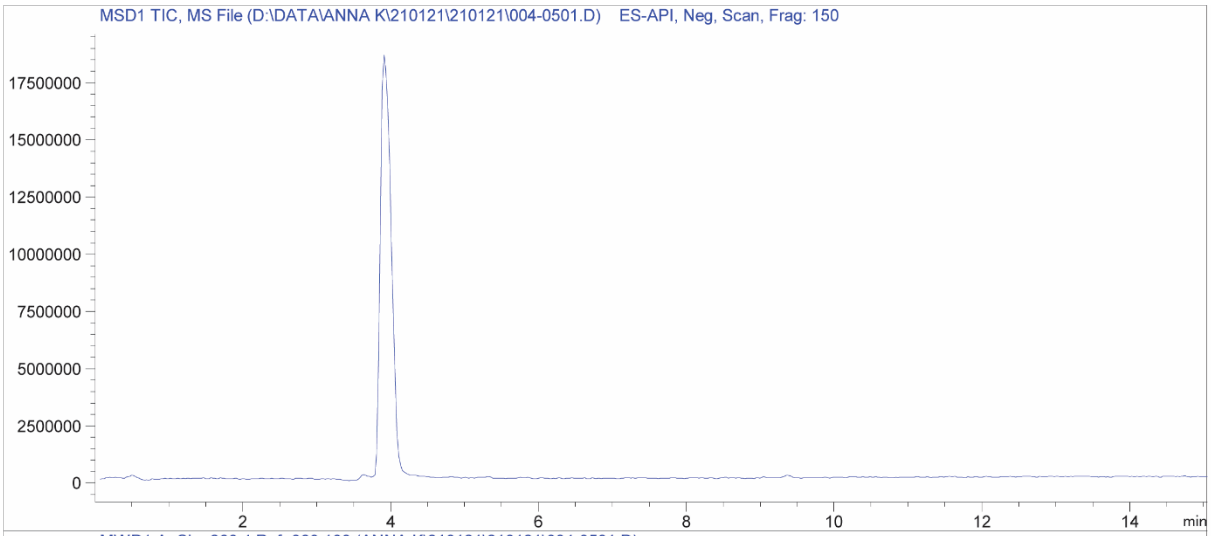

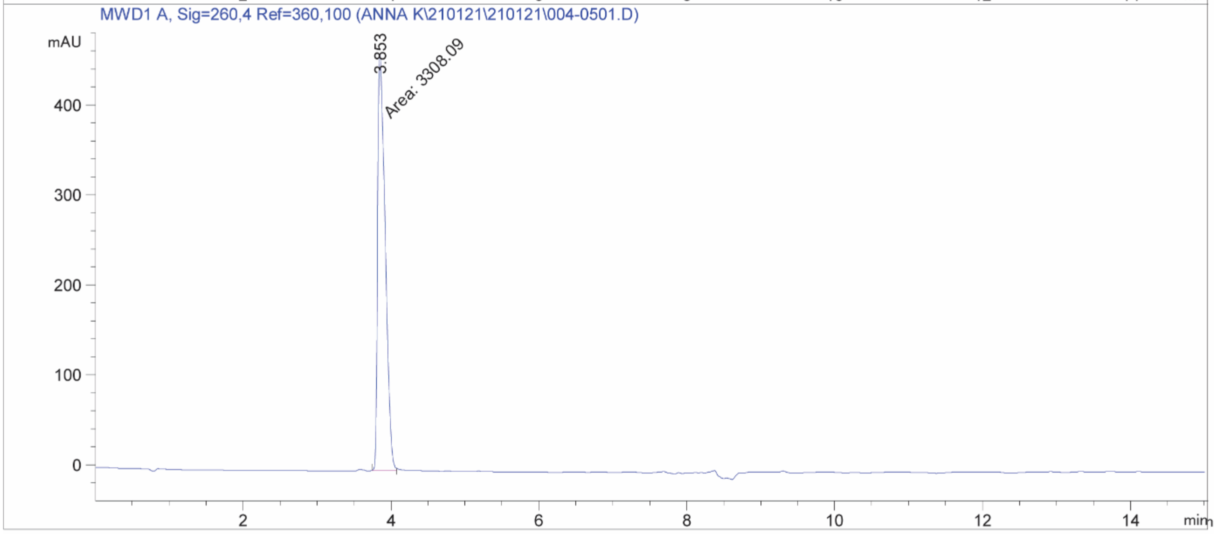

Signal 2: MWD1 A, Sig=260,4 Ref=360,100

Peak #	RetTime [min]	Type	Width [min]	Area [mAU*s]	Height [mAU]	Area %
1	3.853	MM	0.1189	3308.08740	463.67847	100.0000
Totals :				3308.08740	463.67847	

Deconvolution of Spectrum # 1 @ 3.638 - 4.236 min

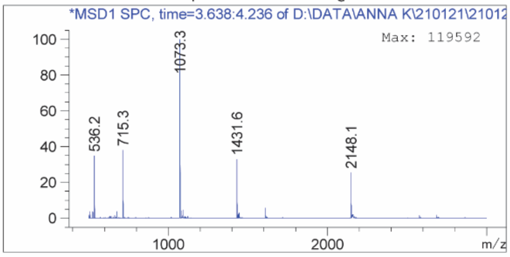

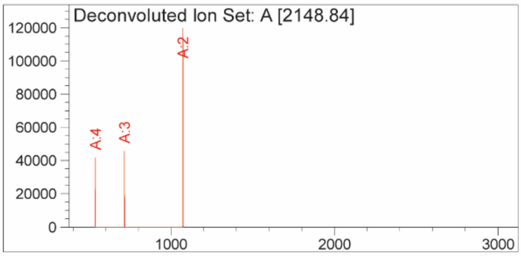

Component	Molecular Weight	Absolute Abundance	Relative Abundance
A	2148.84	206156	100.00

*** End of Report ***

*U*A*C*A*U*A*U

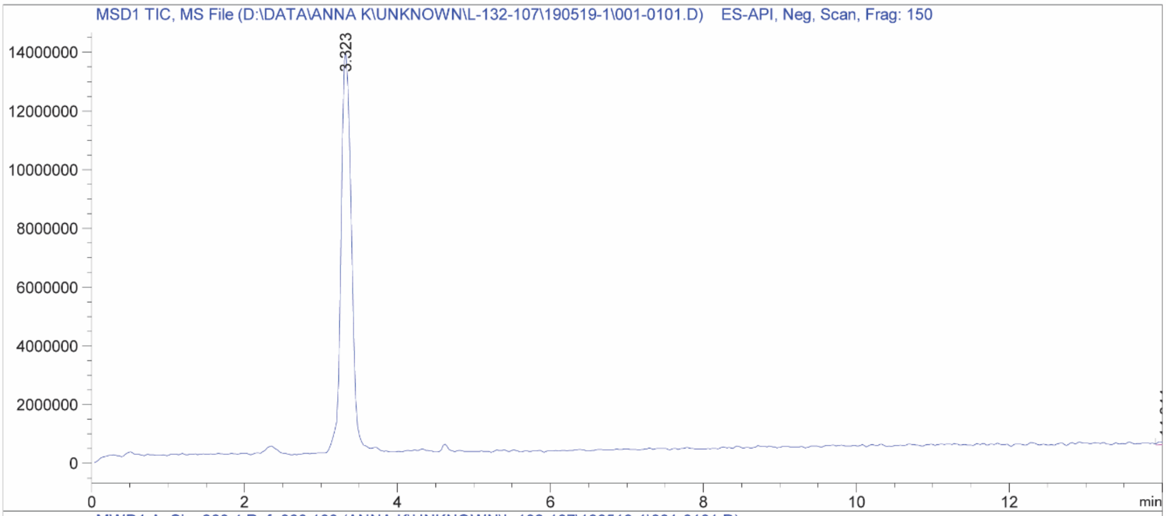

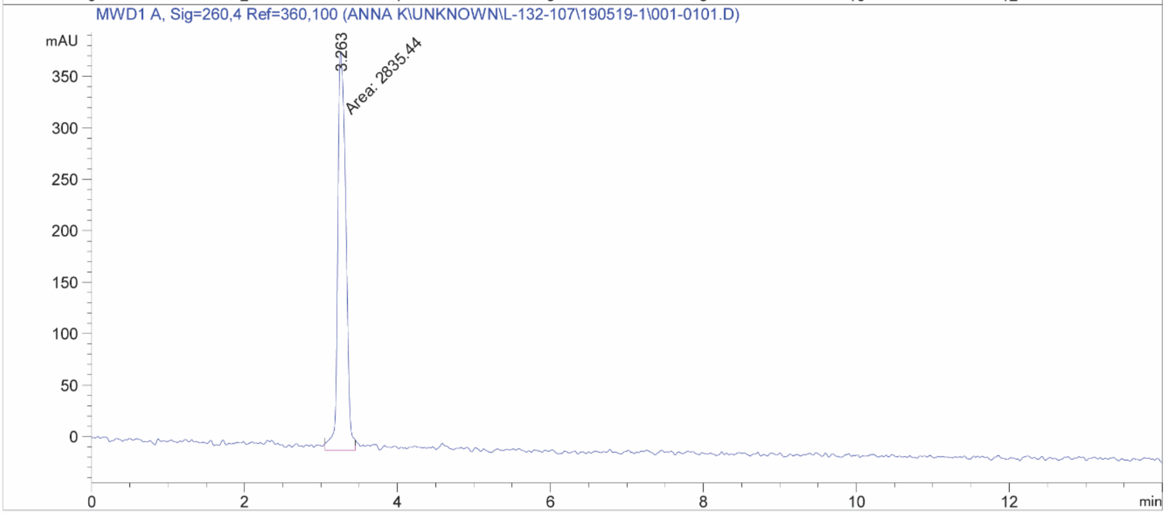

Signal 2: MWD1 A, Sig=260,4 Ref=360,100

Peak #	RetTime [min]	Type	Width [min]	Area [mAU*s]	Height [mAU]	Area %
1	3.263	MM	0.1221	2835.43848	386.98477	99.1949
2	14.297	VV	0.0662	23.01476	5.56820	0.8051

Totals : 2858.45323 392.55297

Deconvolution of Spectrum # 1 @ 3.131 - 3.530 min

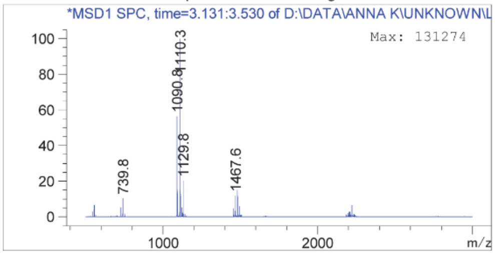

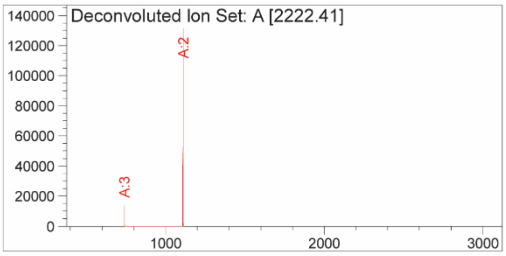

Component	Molecular Weight	Absolute Abundance	Relative Abundance
A	2222.41	144614	100.00

UGCCUGU

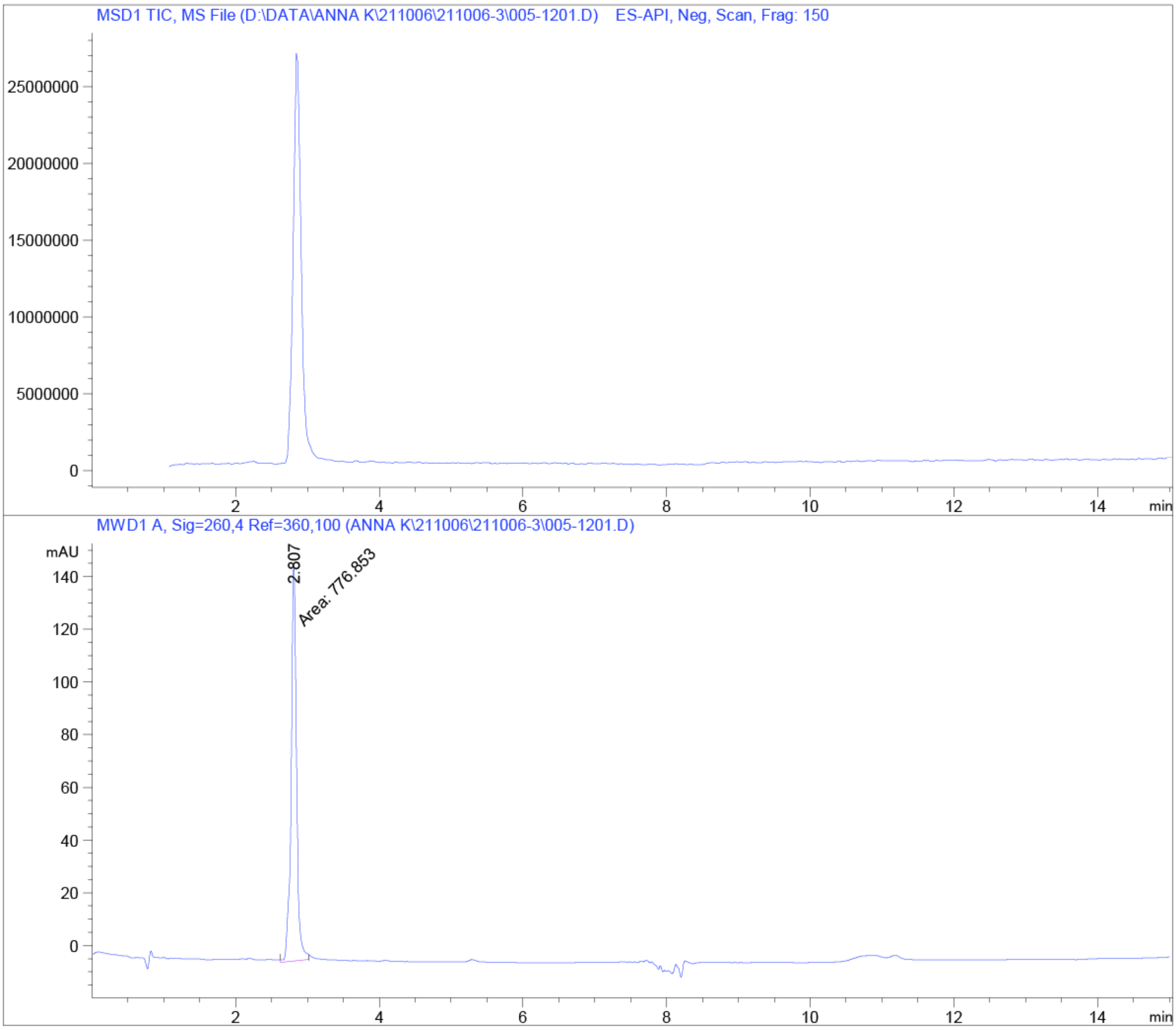

Signal 2: MWD1 A, Sig=260,4 Ref=360,100

Peak #	RetTime [min]	Type	Width [min]	Area [mAU*s]	Height [mAU]	Area %
1	2.807	MM	0.0856	776.85333	151.20306	100.0000
Totals :				776.85333	151.20306	

Deconvolution of Spectrum # 1 @ 2.718 - 3.042 min

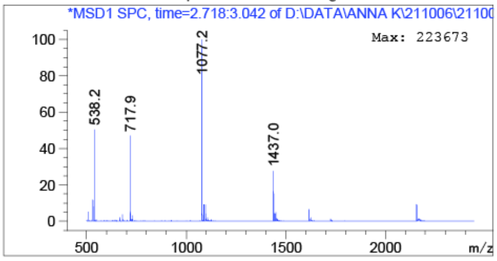

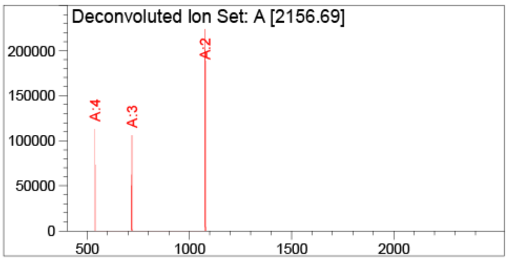

Component	Molecular Weight	Absolute Abundance	Relative Abundance
A	2156.69	439850	100.00

*** End of Report ***

*U*G*C*C*U*G*U

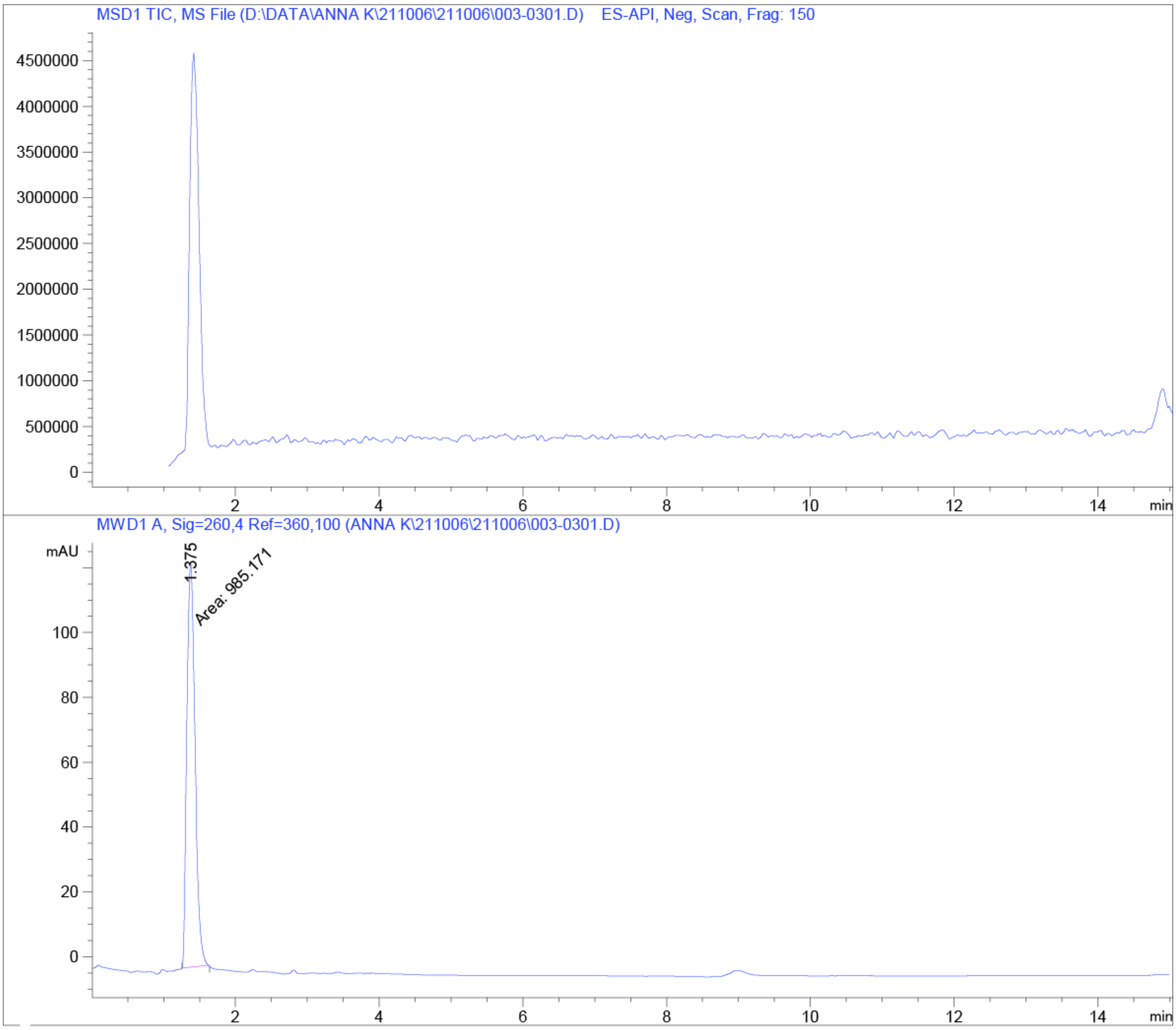

Deconvolution of Spectrum # 1 @ 1.074 - 2.743 min

Signal 2: MWD1 A, Sig=260,4 Ref=360,100

Peak #	RetTime [min]	Type	Width [min]	Area [mAU*s]	Height [mAU]	Area %
1	1.375	MM	0.1315	985.17108	124.83763	100.0000
Totals :				985.17108	124.83763	

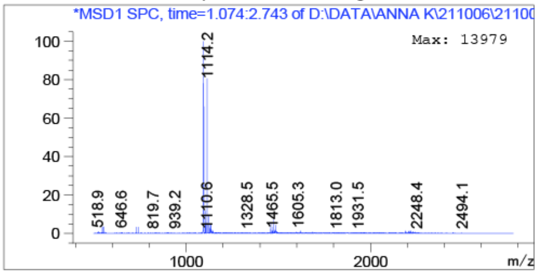

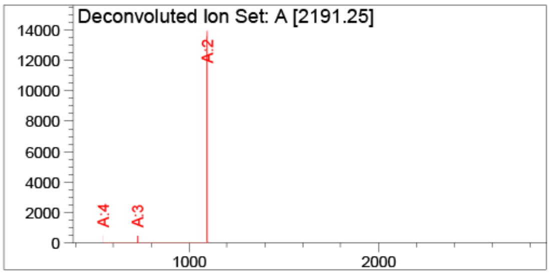

Component	Molecular Weight	Absolute Abundance	Relative Abundance
A	2191.25	14820	100.00
B	2230.30	12025	81.14

*** End of Report ***

UGCACGU

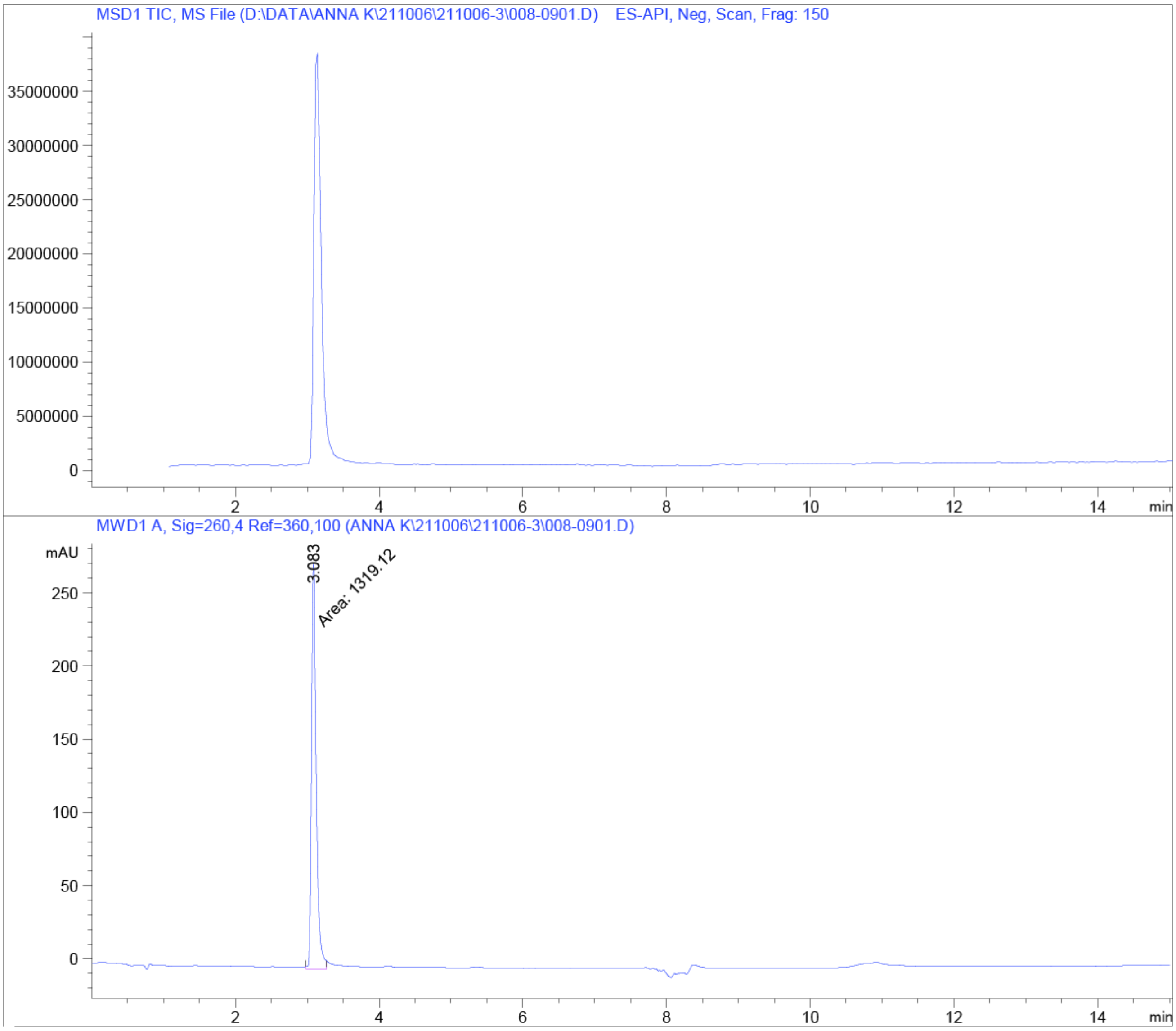

Signal 2: MWD1 A, Sig=260,4 Ref=360,100

Peak #	RetTime [min]	Type	Width [min]	Area [mAU*s]	Height [mAU]	Area %
1	3.083	MM	0.0789	1319.11597	278.59128	100.0000

Totals : 1319.11597 278.59128

Deconvolution of Spectrum # 1 @ 2.942 - 3.366 min

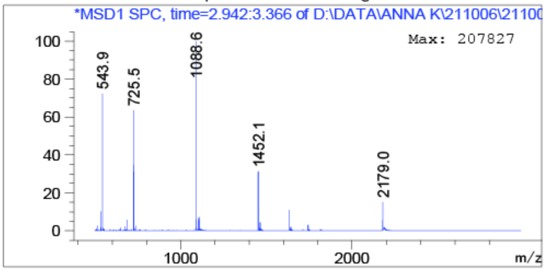

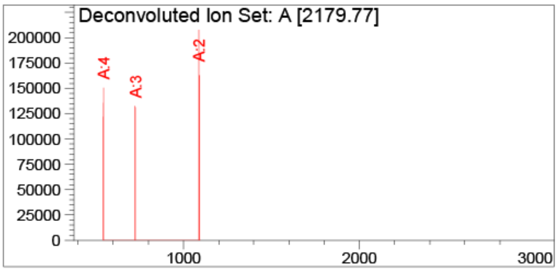

Component	Molecular Weight	Absolute Abundance	Relative Abundance
A	2179.77	478668	100.00

*** End of Report ***

*U*G*C*A*C*G*U

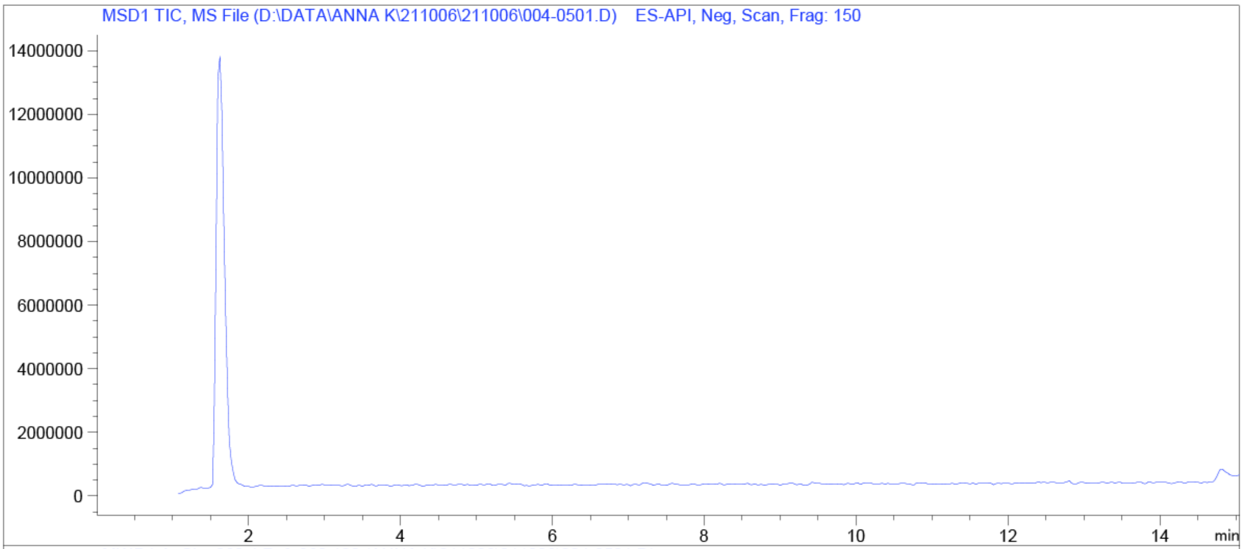

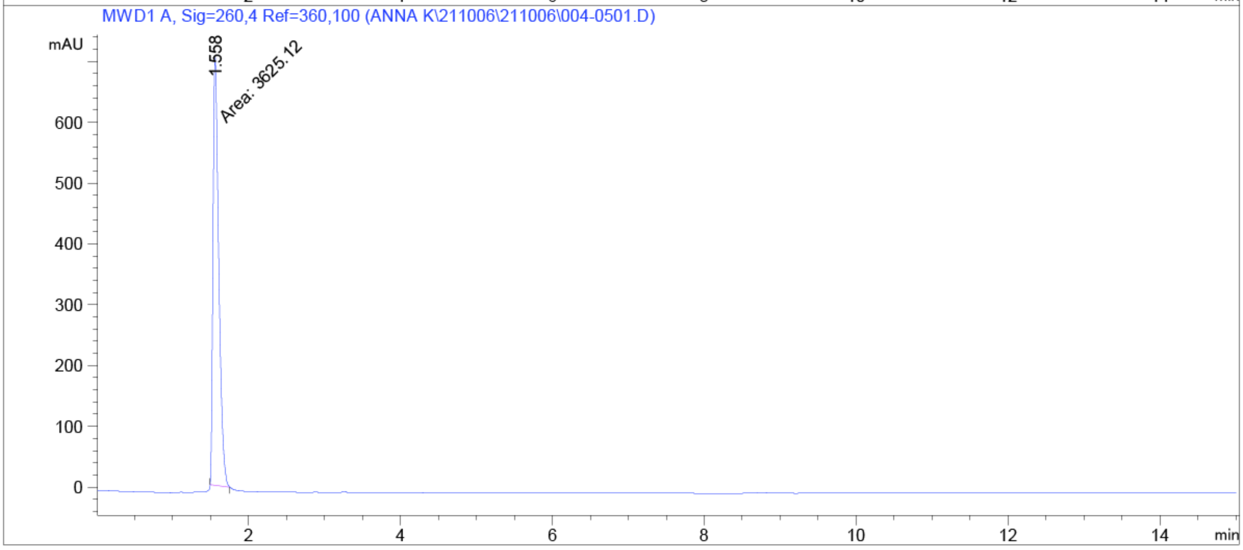

Signal 2: MWD1 A, Sig=260,4 Ref=360,100

Peak #	RetTime [min]	Type	Width [min]	Area [mAU*s]	Height [mAU]	Area %
1	1.558	PM	0.0854	3625.11987	707.70569	100.0000
Totals :				3625.11987	707.70569	

Deconvolution of Spectrum # 1 @ 1.273 - 2.270 min

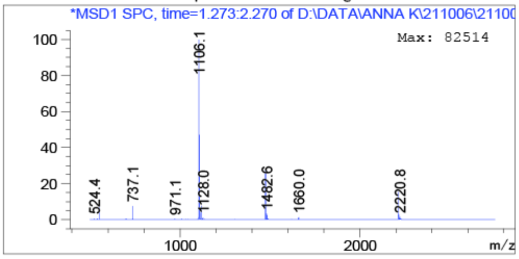

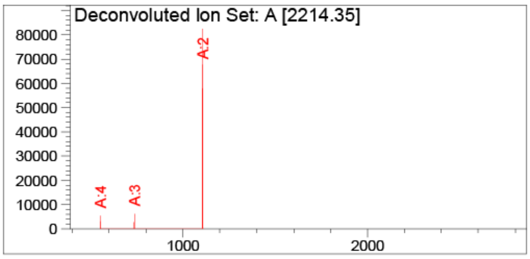

Component	Molecular Weight	Absolute Abundance	Relative Abundance
A	2214.35	93206	100.00

*** End of Report ***

UGCAUCU

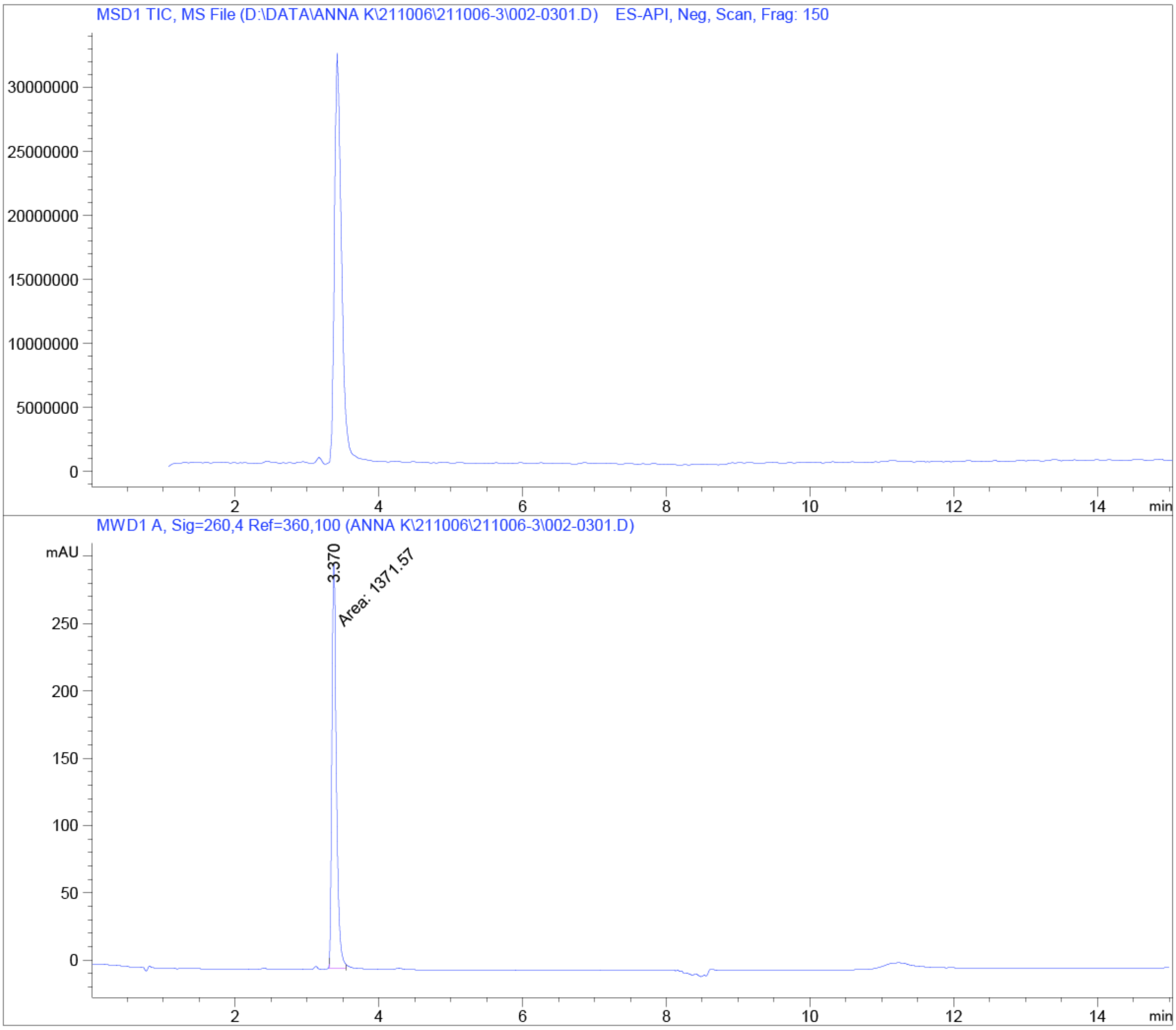

Signal 2: MWD1 A, Sig=260,4 Ref=360,100

Peak #	RetTime [min]	Type	Width [min]	Area [mAU*s]	Height [mAU]	Area %
1	3.370	MM	0.0756	1371.57446	302.46481	100.0000

Totals : 1371.57446 302.46481

Deconvolution of Spectrum # 1 @ 2.942 - 3.714 min

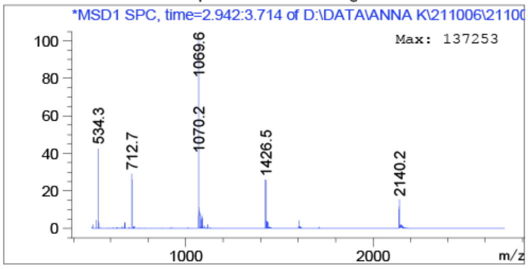

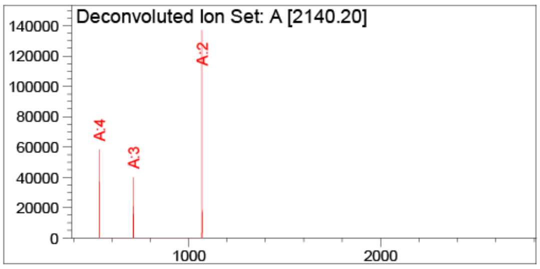

Component	Molecular Weight	Absolute Abundance	Relative Abundance
A	2140.20	210950	100.00

*** End of Report ***

*U*G*C*A*U*C*U

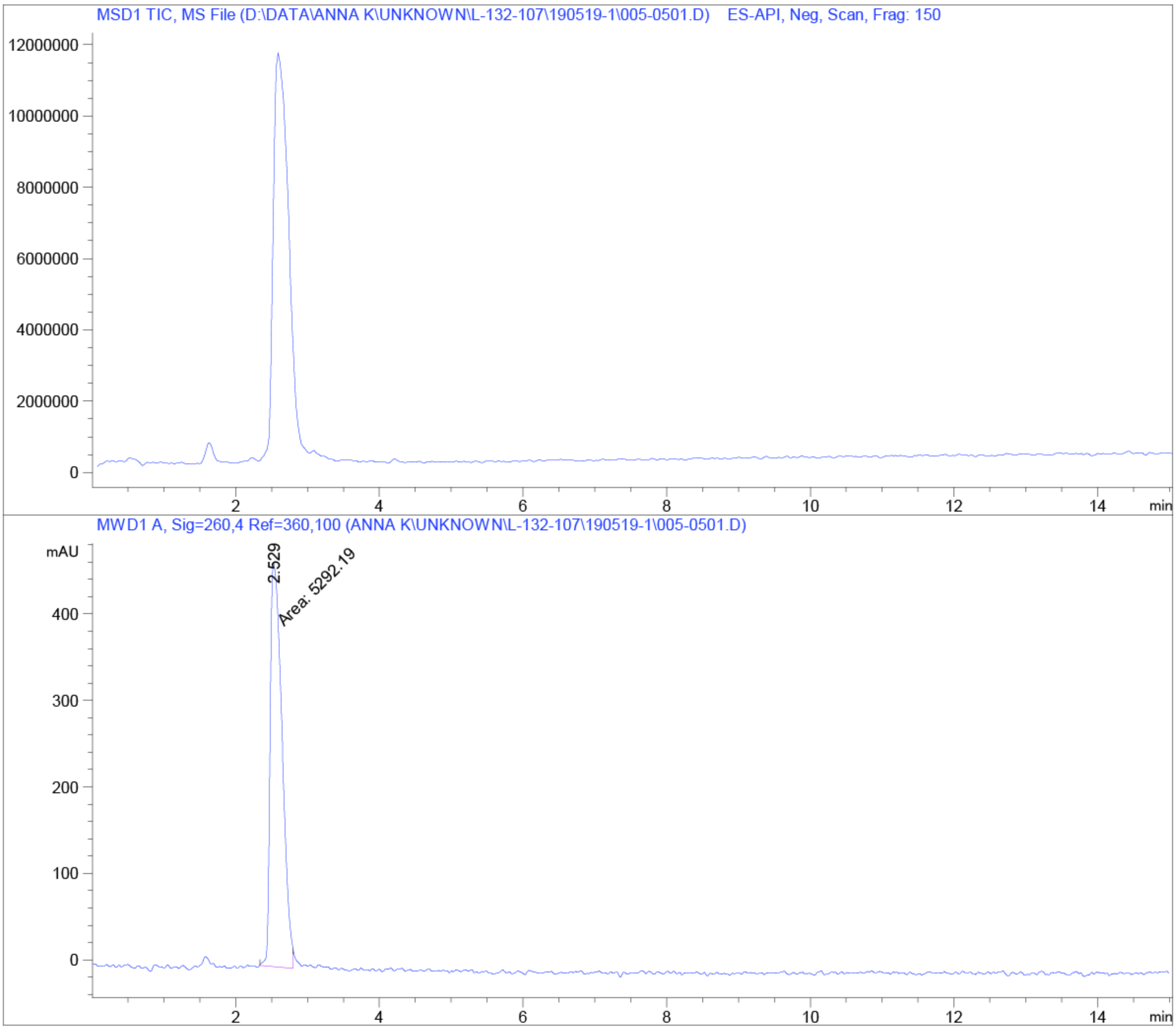

Signal 2: MWD1 A, Sig=260,4 Ref=360,100

Peak #	RetTime [min]	Type	Width [min]	Area [mAU*s]	Height [mAU]	Area %
1	2.529	PM	0.1893	5292.18604	465.92816	100.0000

Totals : 5292.18604 465.92816

Deconvolution of Spectrum # 1 @ 2.616 min

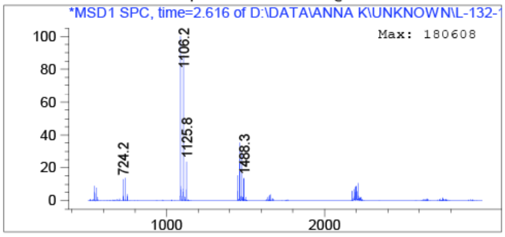

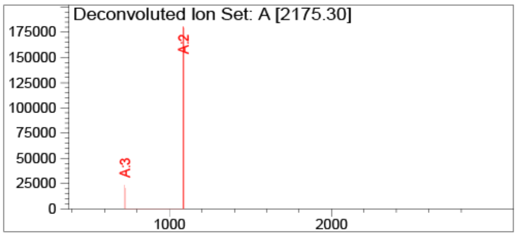

Component	Molecular Weight	Absolute Abundance	Relative Abundance
A	2175.30	203667	100.00
B	2214.29	184646	90.66

*** End of Report ***

UGCAUGC

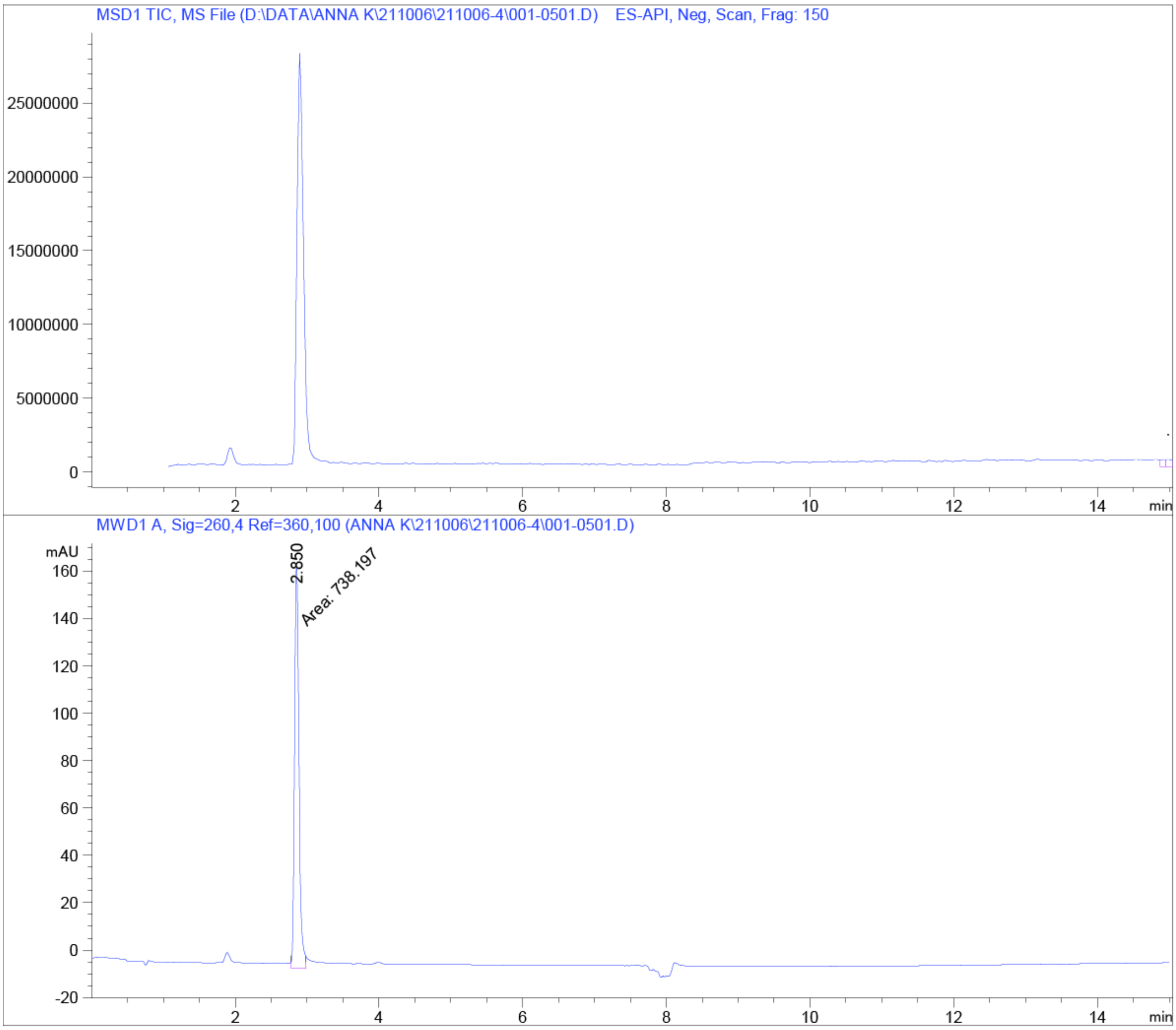

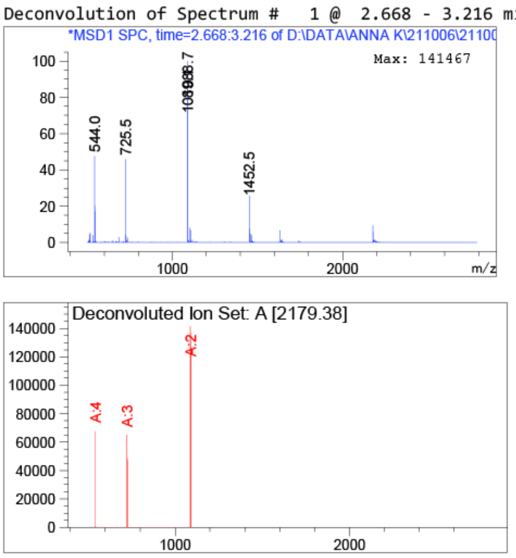

Signal 2: MWD1 A, Sig=260,4 Ref=360,100

Peak #	RetTime [min]	Type	Width [min]	Area [mAU*s]	Height [mAU]	Area %
1	2.850	MM	0.0718	738.19702	171.43054	100.0000
Totals :				738.19702	171.43054	

Component	Molecular Weight	Absolute Abundance	Relative Abundance
A	2179.38	263903	100.00
B	4359.31	174358	66.07

*** End of Report ***

*U*G*C*A*U*G*C

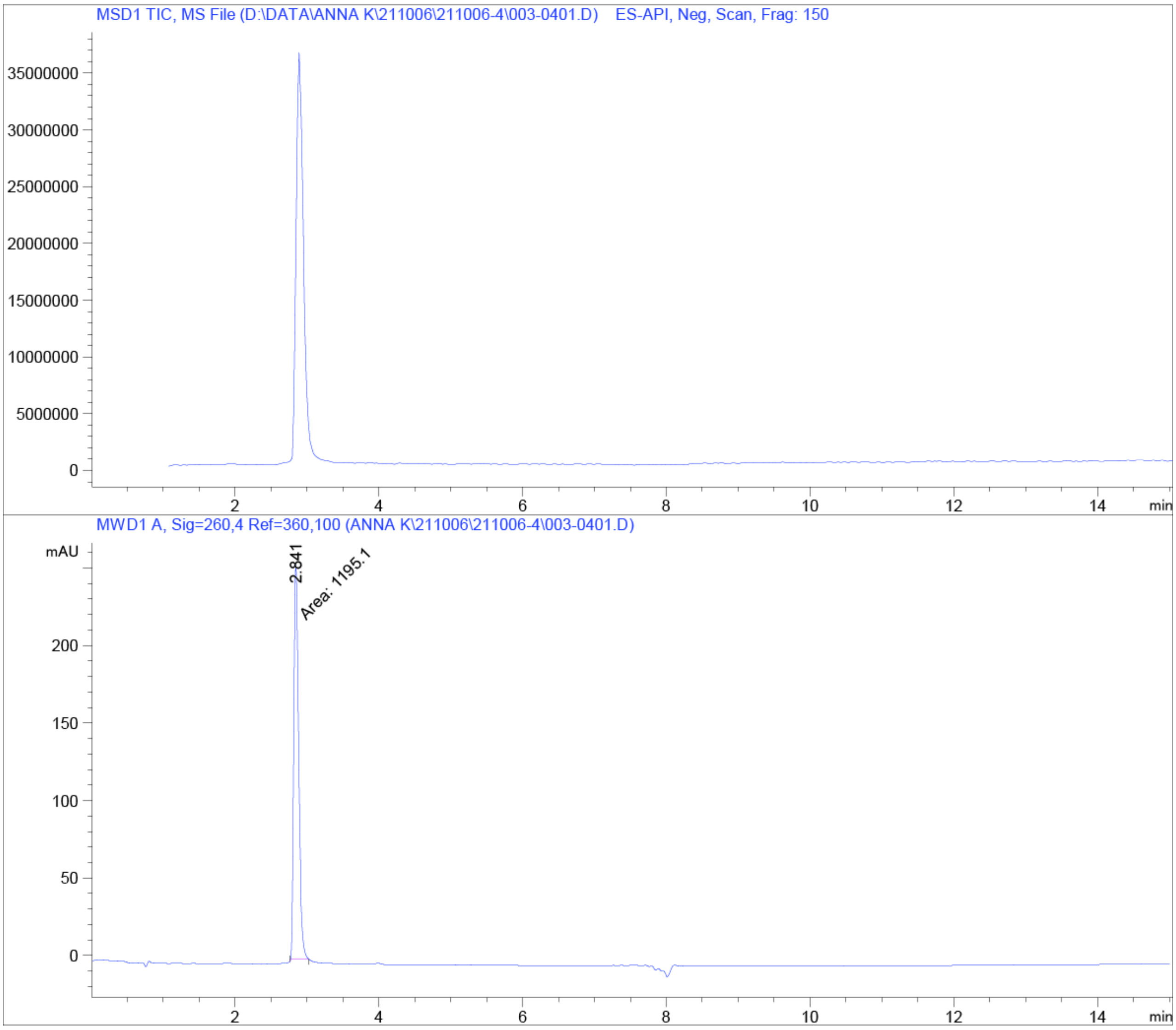

Signal 2: MWD1 A, Sig=260,4 Ref=360,100

Peak #	RetTime [min]	Type	Width [min]	Area [mAU*s]	Height [mAU]	Area %
1	2.841	MM	0.0777	1195.09753	256.33649	100.0000
Totals :				1195.09753	256.33649	

Deconvolution of Spectrum # 1 @ 2.768 - 3.067 min

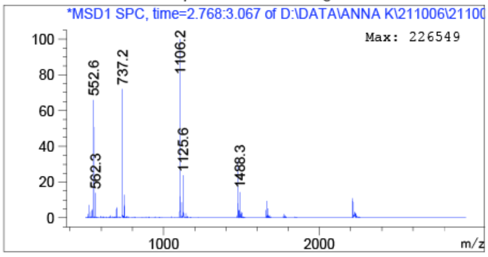

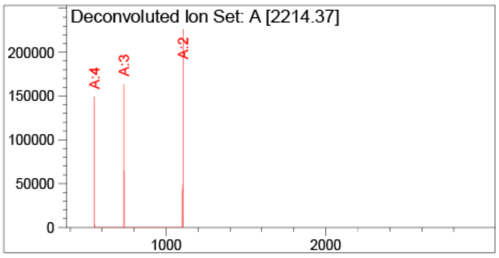

Component	Molecular Weight	Absolute Abundance	Relative Abundance
A	2214.37	532902	100.00
B	4428.62	297743	55.87

*** End of Report ***

Supplementary Figure 10: LC-MS traces of synthesised oligonucleotide sequences.

Supplementary Table 1. Variants of the FOX_{RBE} used in this study.

Sequence	$Mass_{found}$	$Mass_{calc}$	UV Purity
*U*G*C*A*U*G*U	2215.4	2216.4	>99%
AGCAUGU	2203.8	2204.0	97%
*A*G*C*A*U*G*U	2277.5	2278.0 $[M+K]^+$	>99%
*AGCAUGU	2208.7	2209.0	>99%
CGCAUGU	2179.9	2180.0	>99%
*C*G*C*A*U*G*U	2214.4	2215.0	>99%
*CGCAUGU	2184.8	2185.0	>99%
GGCAUGU	2219.8	2219.8	>99%
*G*G*C*A*U*G*U	2254.4	2254.8	>99%
*GGCAUGU	2224.9	2225.0	>99%
UGGAUGU	2220.8	2221.5	>99%
*U*G*G*A*U*G*U	2255.4	2256.5	>99%
UG*GAUGU	2225.8	2226.5	>99%
UGCGUGU	2196.8	2197.4	>99%
*U*G*C*G*U*G*U	2231.4	2232.4	>99%
UGC*GUGU	2201.8	2202.4	>99%
UGCAGGU	2219.8	2220.4	>99%
*U*G*C*A*G*G*U	2254.4	2255.4	>99%
UGCA*GGU	2224.7	2225.4	96%
UGCAUGG	2220.7	2220.4	>99%
*U*G*C*A*U*G*G	2254.4	2255.4	>99%
UGCAUG*G	2224.9	2225.4	>99%
UGUAUGU	2181.9	2182.4	>99%
*U*G*U*A*U*G*U	2216.4	2217.4	>99%
UG*UAUGU	2186.7	2187.4	96%
UGCUUGU	2157.8	2158.4	>99%
*U*G*C*U*U*G*U	2192.3	2193.4	>99%
UGC*UUGU	2162.7	2163.4	>99%
UACAUGU	2164.6	2165.4	>99%
*U*A*C*A*U*G*U	2199.4	2200.4	>99%
UGCAUAU	2164.9	2165.4	>99%
*U*G*C*A*U*A*U	2199.4	2200.4	>99%
UACAUAU	2148.9	2149.4	>99%
*U*A*C*A*U*A*U	2222.4	2223.4 $[M+K]^+$	>99%
UGCCUGU	2156.7	2157.3	>99%
*U*G*C*C*U*G*U	2191.3	2192.3	>99%
UGCACGU	2179.8	2180.4	>99%
*U*G*C*A*C*G*U	2214.4	2215.4	>99%
UGCAUCU	2140.2	2141.3	>99%
*U*G*C*A*U*C*U	2175.3	2176.3	>99%
UGCAUGC	2179.4	2180.4	>99%
*U*G*C*A*U*G*C	2214.4	2215.4	>99%

Supplementary Data 1: List of plotted protein-RNA cross-links (separate excel file)

Supplementary Data 2: List of identified protein-RNA cross-links (separate excel file)

Supplementary Data 3: Table of RNA seqs and neutral losses used for xQuest analyses (separate excel file)

Supplementary Data 4: Analysis of large-scale datasets of Kramer et al and Bae et al. (separate excel file)

Supplementary References

1 Dorn, G. *et al*. Structural modeling of protein-RNA complexes using crosslinking of segmentally isotope-labeled RNA and MS/MS. *Nat Methods* **14**, 487-490, (2017).

2 Walzthoeni, T. *et al.* False discovery rate estimation for cross-linked peptides identified by mass spectrometry. *Nature Methods* **9**, 901-903, (2012).

3 Perez-Riverol, Y. *et al.* The PRIDE database and related tools and resources in 2019: improving support for quantification data. *Nucleic Acids Research* **47**, D442-D450, (2018).

4 Kramer, K. *et al.* Photo-cross-linking and high-resolution mass spectrometry for assignment of RNA-binding sites in RNA-binding proteins. *Nat Methods* **11**, 1064-1070, (2014).

5 Bae, J. W., Kwon, S. C., Na, Y., Kim, V. N. & Kim, J.-S. Chemical RNA digestion enables robust RNA-binding site mapping at single amino acid resolution. *Nature Structural & Molecular Biology* **27**, 678–682, (2020).

6 Auweter, S. D. *et al.* Molecular basis of RNA recognition by the human alternative splicing factor Fox-1. *The EMBO journal* **25**, 163-173, (2006).

7 Frederico, L. A., Kunkel, T. A. & Shaw, B. R. A sensitive genetic assay for the detection of cytosine deamination: determination of rate constants and the activation energy. *Biochemistry* **29**, 2532-2537, (1990).

8 Green, M. & Cohen, S. S. Studies on the biosynthesis of bacterial and viral pyrimidines III. Derivatives of dihydrocytosine. *Journal of Biological Chemistry* **228**, 601-609, (1957).

9 Oberstrass, F. C. *et al.* Structure of PTB Bound to RNA: Specific Binding and Implications for Splicing Regulation. *Science* **309**, 2054-2057, (2005).
