# Supplementary material for: Nucleotide-amino acid π-stacking interactions initiate photo cross-linking in RNA-protein complexes
Source: Nat Commun. 2022 May 17;13:2719. doi: 10.1038/s41467-022-30284-w (PMC9114321; doi:10.1038/s41467-022-30284-w)
Supplement: Supplementary file 2 — Description of Additional Supplementary Files [file 41467_2022_30284_MOESM2_ESM.pdf]

File name: Supplementary Data 1

Description: List of protein-RNA cross-links plotted in Figs. 1B., 1C, 2C, 2F, and 3B.

File name: Supplementary Data 2

Description: Identified protein-RNA cross-links presented in Figs. 1,2 and 3 and Supplementary Figs. 1, 3, 4, 5, and 6.

File name: Supplementary Data 3

Description: Table of all RNA sequences and neutral losses that were used for xQuest analyses:

File name: Supplementary Data 4

Description: Tables and analyses of localized protein-RNA cross-links taken from the supporting data from Kramer et al1] and Bae et al. [2].
