# Supplementary material for: Nucleotide-amino acid π-stacking interactions initiate photo cross-linking in RNA-protein complexes
Source: Nat Commun. 2022 May 17;13:2719. doi: 10.1038/s41467-022-30284-w (PMC9114321; doi:10.1038/s41467-022-30284-w)
Supplement: Supplementary file 7 — Reporting Summary [file 41467_2022_30284_MOESM7_ESM.pdf]

nature portfolio

Corresponding author(s): Hall

Last updated by author(s): Mar 21, 2022

Reporting Summary

Nature Portfolio wishes to improve the reproducibility of the work that we publish. This form provides structure for consistency and transparency in reporting. For further information on Nature Portfolio policies, see our Editorial Policies and the Editorial Policy Checklist.

Statistics

For all statistical analyses, confirm that the following items are present in the figure legend, table legend, main text, or Methods section.

n/a	Confirmed	
☐	☒	The exact sample size (n) for each experimental group/condition, given as a discrete number and unit of measurement
☐	☒	A statement on whether measurements were taken from distinct samples or whether the same sample was measured repeatedly
☒	☐	The statistical test(s) used AND whether they are one- or two-sided<br>*Only common tests should be described solely by name; describe more complex techniques in the Methods section.*
☒	☐	A description of all covariates tested
☒	☐	A description of any assumptions or corrections, such as tests of normality and adjustment for multiple comparisons
☒	☐	A full description of the statistical parameters including central tendency (e.g. means) or other basic estimates (e.g. regression coefficient) AND variation (e.g. standard deviation) or associated estimates of uncertainty (e.g. confidence intervals)
☒	☐	For null hypothesis testing, the test statistic (e.g. F, t, r) with confidence intervals, effect sizes, degrees of freedom and P value noted<br>*Give P values as exact values whenever suitable.*
☒	☐	For Bayesian analysis, information on the choice of priors and Markov chain Monte Carlo settings
☒	☐	For hierarchical and complex designs, identification of the appropriate level for tests and full reporting of outcomes
☒	☐	Estimates of effect sizes (e.g. Cohen's d, Pearson's r), indicating how they were calculated

Our web collection on statistics for biologists contains articles on many of the points above.

Software and code

Policy information about availability of computer code

Data collection	Data were acquired using Thermo Fisher Scientific mass spectrometers, using Thermo Fisher XCalibur for data collection (4.2.47 on Orbitrap Lumos, 3.0.63 on Orbitrap Elite). Data were recorded in proprietary Thermo Fisher RAW format.
Data analysis	.RAW files were converted to mzXML files, using msconvert.exe (ProteoWizard msConvert v.3.0.9393c). The .mzXML files were then searched using xQuest V2.1.5, available from https://gitlab.ethz.ch/leitner_lab/xquest_xprophet . Parameters for data search are included in the methods sections of the main text. Outputs from xQuest analysis were plotted using the Plotly 4.6.0 package in Python 3.7.<br>The structures were visualized in PyMOL Molecular Graphics System, Version 2.5 Schrödinger, LLC.

For manuscripts utilizing custom algorithms or software that are central to the research but not yet described in published literature, software must be made available to editors and reviewers. We strongly encourage code deposition in a community repository (e.g. GitHub). See the Nature Portfolio guidelines for submitting code & software for further information.

Data

Policy information about availability of data

All manuscripts must include a data availability statement. This statement should provide the following information, where applicable:

- Accession codes, unique identifiers, or web links for publicly available datasets
- A description of any restrictions on data availability
- For clinical datasets or third party data, please ensure that the statement adheres to our policy

The mass spectrometry proteomics data have been deposited at the ProteomeXchange Consortium via the PRIDE partner repository with the dataset identifier PXD031381.
The referenced accession codes for the structures in the Protein Data Bank are 2ERR (http://doi.org/10.2210/pdb2ERR/pdb), 2AD9 (http://doi.org/10.2210/

pdb2AD9/pdb), 2ADB (http://doi.org/10.2210/pdb2ADB/pdb), 2ADC (http://doi.org/10.2210/pdb2ADC/pdb) , 3TS0 (http://doi.org/10.2210/pdb3TS0/pdb), 2MXY (http://doi.org/10.2210/pdb2MXY/pdb), 4V88 (http://doi.org/10.2210/pdb4V88/pdb), 2YH1 (http://doi.org/10.2210/pdb2YH1/pdb), 4ZT0 (http://doi.org/10.2210/pdb4ZT0/pdb).

Field-specific reporting

Please select the one below that is the best fit for your research. If you are not sure, read the appropriate sections before making your selection.

☒ Life sciences ☐ Behavioural & social sciences ☐ Ecological, evolutionary & environmental sciences

For a reference copy of the document with all sections, see nature.com/documents/nr-reporting-summary-flat.pdf

Life sciences study design

All studies must disclose on these points even when the disclosure is negative.

Sample size	Experimental observations are qualitative in nature, and no statistical testing was performed on results.
Data exclusions	No data were excluded.
Replication	Each SDS-page was repeated successfully three times. Each reconstituted protein-RNA complex sample was split into two, and prepared for CLIR-MS analysis for duplicate experiments. The obtained results were aggregated as is conventional for cross-linking and mass spectrometry experiments.
Randomization	There is no experimental grouping that is relevant to the study design.
Blinding	Blinding was not relevant as there is no grouping which impacts the findings.

Reporting for specific materials, systems and methods

We require information from authors about some types of materials, experimental systems and methods used in many studies. Here, indicate whether each material, system or method listed is relevant to your study. If you are not sure if a list item applies to your research, read the appropriate section before selecting a response.

Materials & experimental systems

n/a	Involved in the study	
☒	☐	Antibodies
☒	☐	Eukaryotic cell lines
☒	☐	Palaeontology and archaeology
☐	☒	Animals and other organisms
☒	☐	Human research participants
☒	☐	Clinical data
☒	☐	Dual use research of concern

Methods

n/a	Involved in the study	
☒	☐	ChIP-seq
☒	☐	Flow cytometry
☒	☐	MRI-based neuroimaging

Animals and other organisms

Policy information about studies involving animals; ARRIVE guidelines recommended for reporting animal research

Laboratory animals	E. coli BL21 (DE3) codon+ (RIL) (Agilent Technologies) were used for protein expression.
Wild animals	No wild animals were used.
Field-collected samples	No wild animals were used.
Ethics oversight	Ethical approval was not required for E. coli culture in which samples were produced.

Note that full information on the approval of the study protocol must also be provided in the manuscript.
